# How Is Substrate Halogenation Triggered by the Vanadium Haloperoxidase from *Curvularia inaequalis*?

**DOI:** 10.1021/acscatal.3c00761

**Published:** 2023-06-06

**Authors:** Emilie F. Gérard, Thirakorn Mokkawes, Linus O. Johannissen, Jim Warwicker, Reynard R. Spiess, Christopher F. Blanford, Sam Hay, Derren J. Heyes, Sam P. de Visser

**Affiliations:** † Manchester Institute of Biotechnology, The University of Manchester, 131 Princess Street, Manchester M1 7DN, United Kingdom; ‡ Department of Chemical Engineering, The University of Manchester, Oxford Road, Manchester M13 9PL, United Kingdom; § Department of Chemistry, The University of Manchester, Oxford Road, Manchester M13 9PL, United Kingdom; ∥ School of Biological Sciences, Faculty of Biology, Medicine and Health, The University of Manchester, Oxford Road, Manchester 13 9PL, United Kingdom; ⊥ Department of Materials, The University of Manchester, Oxford Road, Manchester M13 9PL, United Kingdom

**Keywords:** halogen transfer, enzyme mechanism, enzyme catalysis, inorganic reaction mechanism, modeling, stopped-flow

## Abstract

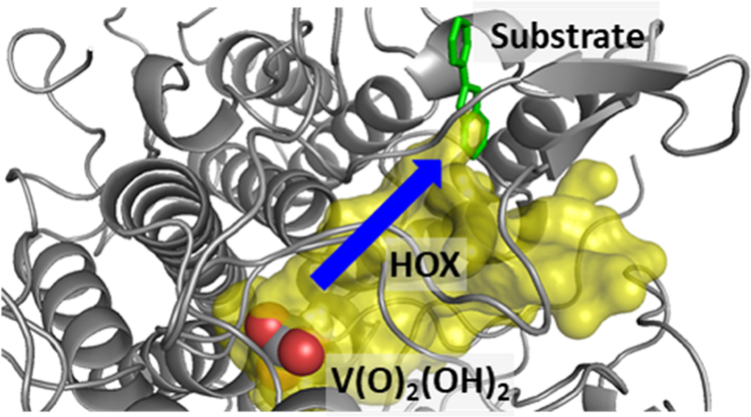

Vanadium haloperoxidases (VHPOs) are unique enzymes in biology that catalyze a challenging halogen transfer reaction and convert a strong aromatic C–H bond into C–X (X = Cl, Br, I) with the use of a vanadium cofactor and H_2_O_2_. The VHPO catalytic cycle starts with the conversion of hydrogen peroxide and halide (X = Cl, Br, I) into hypohalide on the vanadate cofactor, and the hypohalide subsequently reacts with a substrate. However, it is unclear whether the hypohalide is released from the enzyme or otherwise trapped within the enzyme structure for the halogenation of organic substrates. A substrate-binding pocket has never been identified for the VHPO enzyme, which questions the role of the protein in the overall reaction mechanism. Probing its role in the halogenation of small molecules will enable further engineering of the enzyme and expand its substrate scope and selectivity further for use in biotechnological applications as an environmentally benign alternative to current organic chemistry synthesis. Using a combined experimental and computational approach, we elucidate the role of the vanadium haloperoxidase protein in substrate halogenation. Activity studies show that binding of the substrate to the enzyme is essential for the reaction of the hypohalide with substrate. Stopped-flow measurements demonstrate that the rate-determining step is not dependent on substrate binding but partially on hypohalide formation. Using a combination of molecular mechanics (MM) and molecular dynamics (MD) simulations, the substrate binding area in the protein is identified and even though the selected substrates (methylphenylindole and 2-phenylindole) have limited hydrogen-bonding abilities, they are found to bind relatively strongly and remain stable in a binding tunnel. A subsequent analysis of the MD snapshots characterizes two small tunnels leading from the vanadate active site to the surface that could fit small molecules such as hypohalide, halide, and hydrogen peroxide. Density functional theory studies using electric field effects show that a polarized environment in a specific direction can substantially lower barriers for halogen transfer. A further analysis of the protein structure indeed shows a large dipole orientation in the substrate-binding pocket that could enable halogen transfer through an applied local electric field. These findings highlight the importance of the enzyme in catalyzing substrate halogenation by providing an optimal environment to lower the energy barrier for this challenging aromatic halide insertion reaction.

## Introduction

Natural halogenated metabolites are of scientific and industrial interest due to their anti-inflammatory, antiviral, anticancer, antibacterial, and antifungal properties.^1^ Halogenated compounds are also important components in the chemical industry and are widely used in solvents, pesticides, antifouling, and drugs.^2,3^ In general, the regio- and chemoselectivity of synthesizing C–X bonds in substrates remains a scientific challenge. Currently, most processes in the industry require heavy metals as catalysts for the conversion of aromatic C–H bonds into C–X (X = Cl, Br, I).

Coincidentally, nature has evolved enzymatic systems with first-row transition metals that can perform a halide insertion reaction into aromatic and aliphatic C–H bonds under more benign conditions. Halogenases and haloperoxidases use various mechanisms to transfer a halide (Cl^–^, Br^–^, I^–^) to a substrate, namely, through electrophilic, nucleophilic, or radical-type pathways.^4−10^ Understanding the enzymatic process of inserting a halide atom into an aliphatic or aromatic C–H bond is a crucial step in the biocatalytic application of such enzymes in the biotechnology industry and would make an environmentally benign alternative to the use of toxic metals and solvents.^11^ Bioengineering approaches to produce novel halogenated scaffolds would enable an alternative and sustainable synthesis route for fine chemicals and drugs, and hence extensive research has aimed to understand the reaction mechanisms and substrate scope of halogenases and haloperoxidases.^5,7,10^ The frequent stereo- and regioselectivities of halogenases and haloperoxidases offer the possibility of high product yields at fast turnover rates in more environmentally sustainable conditions.^12−14^

Halogenases can be divided into three categories, which vary according to the mechanism that is used for the halogenation chemistry. These include radical halogenation enzymes (e.g., the α-ketoglutarate-dependent non-heme iron halogenases), nucleophilic halogenation enzymes (e.g., *S*-adenosyl-l-methionine-dependent halogenases), and electrophilic halogenation enzymes (e.g., flavin-dependent halogenases and heme- or vanadium haloperoxidases).^4−10^ The third group represents the electrophilic halogenation enzymes that utilize hydrogen peroxide at a metal cofactor to catalyze a haloperoxidase reaction.^15−18^ The heme haloperoxidases were originally discovered in the 1960s, and their catalytic mechanism has been well established.^19^ Their catalytic cycle starts with hydrogen peroxide binding to the heme and proceeds via a number of proton-relay reactions to form a high-valent iron(IV)-oxo heme cation intermediate, known as Compound I. Compound I then reacts with a halide to form a hypohalide (HOX).^19,20^ The Compound I intermediate has been trapped and characterized with UV–visible, electron paramagnetic resonance, and Mössbauer spectroscopy methods and is known to be a highly active oxidant.^21−24^ The position of the hypohalide once generated in heme haloperoxidases has given rise to much discussion. It is proposed to either bind the iron(III) center in the distal position, to be released from the enzyme active site or to be trapped in the active site but not ligated to iron(III). Kinetic and spectroscopic experiments suggest that oxidized chlorine is not released during turnover.^25^

In contrast to the heme haloperoxidases, much less is known about the catalytic cycle of vanadium haloperoxidases (VHPOs). VHPOs have been widely used in biocatalytic reactions for the halogenation of a range of electron-rich substrates and have therefore become highly attractive targets for industrial applications.^12,26−28^ Unlike the heme-dependent enzymes, there is no oxidation of the vanadium cofactor during enzymatic turnover and the vanadium ion is expected to remain in the +5, vanadium(V), oxidation state throughout the catalytic cycle. The VHPOs, like heme haloperoxidases, are classified by the most electrophilic halogen atom they insert and are found not only in various marine organisms, such as red and brown algae (vanadium bromoperoxidases), but also in fungi and bacteria (vanadium chloroperoxidases).^29−32^ It is believed the halogenated natural products that are formed upon catalysis act as a defense mechanism.^33,34^

**Figure 1:**
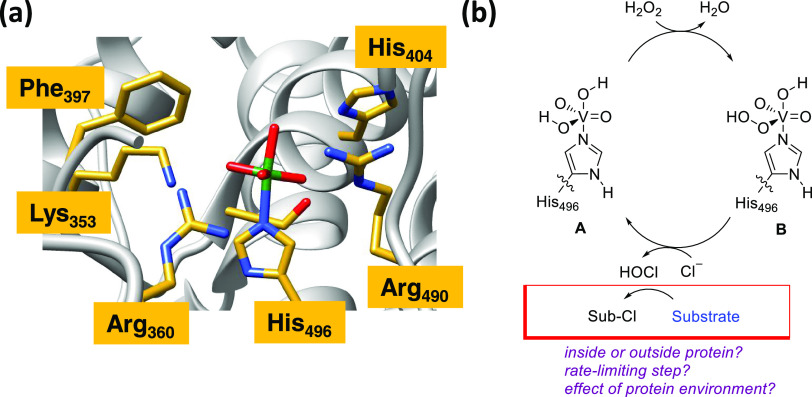
Structure and mechanism of vanadium haloperoxidases (VHPOs). (a) Active site structure of VHPO as taken from the 1IDQ pdb file with the vanadate shown in gray. (b) Proposed catalytic mechanism for the formation of HOCl by VHPOs based on DFT calculations.

*Curvularia inaequalis* vanadium chloroperoxidase (*Ci*VCPO) is a fungal VHPO that is known to halogenate aromatic lignin fragments in wood.^35^ The structure of a number of *Ci*VCPO isozymes has been solved and reveals that the active site contains a vanadate anion bound to a histidine residue of the protein.^36−40^ The active site structure is highly conserved across different species in which the vanadate ligand is in a trigonal bipyramidal coordination geometry with the histidine ligand His_496_ in the axial position, and is further held in position by a number of hydrogen bonds (Figure 1a), e.g., from the polar active site residues Lys_353_, Arg_360_, Ser_402_, His_404_, and Arg_490_.^36,41^ It has also been shown that the coordination geometry of the vanadate center varies between a trigonal bipyramidal native form and a distorted tetragonal pyramid in the peroxo-bound orientation.^41−43^ Experimental and computational studies propose a catalytic cycle that starts with the vanadium(V)-dioxo-dihydroxo resting state (structure **A** in Figure 1b).^44^ This complex reacts with hydrogen peroxide to form the vanadium(V)-dioxo-peroxo complex by the addition of one proton and the reaction is followed by halide attack, hypohalide (HOX) formation, and its release from the active site. Evidence for this version of the catalytic cycle derives from the trapping of the vanadium(V)-dioxo-peroxo complex by crystallography,^41^ spectroscopic studies of long-lived intermediates, and kinetic studies on biomimetic model complexes.^45−49^ However, recent density functional theory (DFT) investigations concluded the vanadium(V)-dioxo-peroxo intermediate to be a dead-end side-product from an alternative proton transfer channel. Instead, a mechanism was suggested whereby the halide reacts to form hypohalide by direct OH^+^ transfer from a vanadium(V)-dioxo(hydroxo)hydroperoxo intermediate (structure **B** in Figure 1b), formed after hydrogen peroxide insertion on the vanadium center, resulting in a shorter-lived intermediate. This mechanism would bypass the formation of a vanadium(V)-dioxo-peroxo complex.^50^ Earlier steady-state kinetic studies of VHPOs agree with the mechanism and proposed a reaction scheme where hydrogen peroxide reacts first, followed by halide and a proton to form an enzyme-bound HOX complex which is then released.^51,52^

Although the mechanism of HOX formation by VHPO enzymes has been studied in detail, the subsequent reaction of the HOX with substrate remains unknown.^53,54^ Indirect evidence suggests the reaction occurs in the enzyme based on chemoselectivity experiments and a lack of oxygen evolution in the presence of electron-rich substrates.^55,56^ However, direct evidence is missing, and no stopped-flow kinetics have been reported. In this work, we will unequivocally show that HOX is not released into solution prior to reacting with substrates. Hence, one of the outstanding questions, in terms of VHPO catalysis, remains whether the substrate binds to the enzyme, and if so, where?

Substrate binding adds complexity to the catalytic cycle and means that several potential steps in the enzymatic reaction could be rate-limiting. Hence, to define the full catalytic cycle of VHPOs and determine the rate-limiting step of the reaction, we have now used a combination of detailed spectroscopic and computational approaches to study the halogenation of indole substrates by *Ci*VHPO. We demonstrate here that substrates bind to VHPOs, and hydrogen peroxide reacts with VHPO first. We identify highly charged regions in the protein that induce a local electric field in the system and guide the formed hypohalide to substrate for a low-energy substrate halogenation step in a cleft on the surface of the protein.

## Methodology

### Protein Expression and Purification

Our approaches follow previous studies from our groups,^57−59^ but details of the procedures are given below. The pET21b plasmid containing the gene for VHPO from *C. inaequalis* (obtained from Invitrogen) was transformed into BL21 DE3 competent *E. coli* cells. The starting culture comprising LB media (5 mL), ampicillin (100 μg mL^–1^), and a single colony of *Ci*VCPO from the LB agar plate was incubated at 37 °C overnight at 190 rpm. The starting cultures were added to 48 × 500 mL of auto induction terrific broth media. These were incubated for 3 h at 30 °C prior to incubation at 20 °C overnight at 180 rpm. The cells were centrifuged for 15 min at 4000 rpm at 4 °C.

A SigmaFAST Protease Inhibitor cocktail tablet (EDTA-free) was added to the resuspended cell culture in buffer A (50 mM Tris base, 1 mM VO_4_^3–^, pH 7.5) and DNAse (10 μg mL^–1^, bovine pancreas, Sigma-Aldrich). A cell disruptor (25 kpsi, Constant Cell Disruption Systems) was used to break the cells open. The cells were centrifuged at 20,000 rpm for 45 min at 4 °C to pellet the cell debris and the resulting supernatant was applied to a His-trap column (5 mL × 2, GE Healthcare) at 4 °C. The resin was washed with 10 column volumes of buffer (50 mM Tris base, 1 mM VO_4_^3–^, pH 7.5, 10 mM imidazole). The protein was eluted stepwise: first, samples were collected in buffer A containing 50 mM imidazole, and second, in buffer A containing 250 mM imidazole. Fractions containing *Ci*VCPO were purified further using a gel filtration column (HiLoad 16/600 Superdex 200 pg). SDS PAGE was used to identify fractions containing *Ci*VCPO (Figure S1, Supporting Information, molecular weight is 67 kDa), and a total of 37.5 mg was recovered.

### Product Isolation and Characterization

2-Phenylindole (3.865 mg, 50 μM) was dissolved in methanol and added to a solution containing 10 mM H_2_O_2_, 200 mM KBr, 50 μM Na_3_VO_4_, and 0.01 μM *Ci*VCPO. The reaction was left for 5 min, and the mixture was extracted three times with ethylacetate (1:1 ratio). The organic fraction was washed with water, then with brine (high concentration of NaCl), and dried using MgSO_4_. The MgSO_4_ was filtered off and the EtOAc was subsequently vacuumed off. The product was a yellow and oily compound and was identified as bromo-2-phenylindole (3.5 mg, 91%). ^1^H NMR (400 MHz, MeOD) δ 7.12 (ddd, *J* = 8.0, 7.1, 1.0 Hz, 1H), 7.19 (ddd, *J* = 8.2, 7.1, 1.3 Hz, 1H), 7.39 (m, 2H), 7.48 (tt, *J* = 6.4, 1.2 Hz, 3H), 7.79 (m, 2H); GCMS *m*/*z* 271, 192.

### Steady-State Measurements

Kinetic studies were carried out inside a quartz cell (Starna Scientific, 4 mm × 10 mm) using a Cary Eclipse fluorescence spectrophotometer. Stock solutions of methylphenylindole or 2-phenylindole (1 mM) were prepared in 100% methanol. *Ci*VCPO (0.01 μM, 5 μL), was added to the reaction mixture made up of methylphenylindole or 2-phenylindole (0–18 μM), MES buffer (50 mM, pH 5.5), potassium bromide or potassium chloride (20 mM), hydrogen peroxide (10 mM), Na_3_VO_4_ (50 μM) and 10% DMSO. The decrease in emission at 380 nm (methylphenylindole) and 376 nm (2-phenylindole) after excitation at 316 nm was followed at room temperature. Standard curves were prepared to follow the decrease in concentrations of the substrates (see Figure S2, Supporting Information). The kinetic constants were calculated by fitting the data to the Michaelis–Menten equation
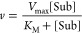
1

### HOX Activity Measurements

*Ci*VCPO (0.01 μM, 50 μL) was added to the reaction mixture made up of MES buffer (50 mM, pH 5.5), potassium bromide (10 mM), hydrogen peroxide (10 mM), Na_3_VO_4_ (50 μM), and 10% DMSO. This solution was pre-mixed for 30 min, and the reaction was then initiated by adding 2-phenylindole (10 μM). The decrease in emission at 376 nm (2-phenylindole) after excitation at 316 nm was followed at room temperature. Enzyme removal of the pre-mixed solution before starting the reaction was carried out by filtering it out via a concentrator (10,000 MWD) or by denaturing by heat shock (90 °C for 5 min). *Ci*VCPO (0.01 μM, 5 μL) was “re-added” to the “enzyme removed” samples and the decrease in fluorescence emission was followed as previously described.

### Stopped-Flow Measurements

Single-turnover fluorescence measurements were acquired using an Applied Photophysics SX20 stopped-flow spectrophotometer. Various mixing strategies were used (indicated in the figure legends) to follow reactions involving 0.5 μM (final concentration) methylphenylindole (or 2-phenylindole), 200 mM potassium bromide or potassium chloride, 250 μM Na_3_VO_4_, 10 mM H_2_O_2_, and 25 μM *Ci*VCPO and 10% DMSO in MES buffer (50 mM, pH 5.5, 10% glycerol at 20 °C). An excitation wavelength of 316 nm and a 350 nm high-pass cutoff filter on the emission PMT detector were used. Data were analyzed using Applied Photophysics SX20 software and five transients were acquired to calculate shot-to-shot error.

### Fluorescence Binding Assay

Binding titrations were carried out on an Edinburgh Instruments F900 fluorescence spectrophotometer. Increasing concentrations of 0.5 μM, *Ci*VCPO was titrated into a solution containing 0.5 μM methylphenylindole in Tris-buffer (50 mM Tris, pH 7.0). The emission was measured at 390 nm after excitation of the sample at 316 nm. The excitation and emission slit parameters were 2.5 nm. The dissociation constant was determined by fitting the experimental data to the tight-binding equation
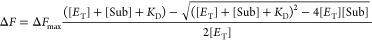
2In eq 2, Δ*F*_max_ represents the maximum change in fluorescence, [*E*_T_] is the enzyme concentration at time T, and [Sub] is the substrate concentration.

### Mass Spectrometry

A 1290 infinity series Agilent LC was used to inject 5 μL of sample into 5% ACN (0.1% FA) and desalted inline using Agilent PLRP-S Guard Cartridges (5 mm × 3 mm) fitted into an appropriate holder. This was eluted over 1 min by 95% ACN. The resulting multiply charged spectrum was analyzed by an Agilent QTOF 6560 ESI QTOF only positive mode and de-convoluted using Agilent MassHunter Bioconfirm software.

### Molecular Docking

The pdb file (1IDQ) of the crystal structure of VCPO from *C. inaequalis* was used, which is an enzymatic monomer with vanadate-bound.^35,41^ Chain A of the enzyme was selected with all crystal water molecules removed and substrate docking was performed using the AutoDock Vina software package for 2-phenylindole, methylphenylindole, erythromycin, styrene, monochlorodimedone, geraniol, and cytosine.^60^ The search volume was set for a cube with 20 Å sides and exhaustiveness of 8 to generate 9 conformations. The highest-scoring structure was selected for the 2-phenylindole- and methylphenylindole-bound structures for further modeling.

### Molecular Dynamics (MD) Simulations

MD simulations were carried out in Gromacs 2020.3^61^ using the Amber14 force field^62^ with a solvation box of water molecules of at least 10 Å from the protein and with counterions generated in AmberTools.^63^ Our *C*iVCPO enzyme models had a total of 94,471 and 94,474 atoms for the 2-phenylindole- and methylphenylindole-bound structures, respectively. The parameters used were at constant pressure (1 bar), 10 Å van der Waals and electrostatic cutoffs, and the particle mesh Ewald approach to describe long-range electrostatics. LINCS bond restraints and periodic boundary conditions were used and a 2 fs time step during the simulation. Bonding parameters for 2-phenylindole and methylphenylindole were generated with the Antechamber module as implemented in AmberTools.^64^ Charges were parameterized by RESP fitting to Gaussian-09 HF/6-31G* optimized structures.^65^ Force constants parameters for vanadate were generated and minimized by fitting to Gaussian-09 distance, angle, and dihedral geometry scans in a B3LYP model with the LANL2DZ basis set on the vanadium atom and 6-31G* on all other atoms (Table 1, Supporting Information).^66−70^ Unrestrained MD simulations with 2-phenylindole or methylphenylindole were performed to confirm the stability of the substrates bound to the enzyme. The MD simulations were run for 110 and 500 ns.

In addition, MD simulations for 500 ns were run for halogenated products by docking 2-phenyl-3-chloroindole and 2-phenyl-3-bromoindole in the structure described above.

### Umbrella Sampling

The potential of mean force (PMF) for HOCl binding in the substrate channel was calculated as a function of the distance between the center of mass of the vanadate and the center of mass of HOCl. Sampling was carried out in bins 2 Å apart with a force constant of 10 kJ mol^–1^ Å^–2^, for a total of 10 ns per bin, starting in the vanadate active site.

### Density Functional Theory (DFT) Calculations

DFT calculations were conducted using Gaussian-09 at the UB3LYP level of theory.^65−67^ The model setup represents 2-phenylindole or methylphenylindole and a hypohalide (HOBr/HOCl). Initial geometry optimizations, frequencies, and geometry scans utilized the 6-31G* basis set on all atoms (basis set BS1). To improve the accuracy of the energies, single-point calculations were performed using the 6-311+G* basis set on all atoms (basis set BS2). Calculations were performed in the gas phase as well as with a continuum-polarized conductor model (CPCM) included with a dielectric constant of 32.7.^71^ Local minima and transition states were characterized by a frequency calculation. The transition states reported a single imaginary mode for the correct transition. Free energies were calculated at 298 K and 1 bar, and these include relative enthalpies with zero-point (ZPE), solvent (*E*_solv_), and entropic corrections. In general, free energies follow the same trends as Δ*E* + ZPE + *E*_solv_ as seen in the Supporting information, and hence, we will use the latter in the main text. These methods have been used previously to calculate free energies of activation of inorganic reaction mechanisms and although they generally give a systematic error, it is expected to be of the order of 3–5 kcal mol^–1^.^72−74^

Electric field calculations were performed to mimic the presence of a protein on the halogenation of the indole derivatives using approaches reported previously.^75,76^ External electric fields were applied along the molecular *x-*, *y-* and *z-*axes of the optimized reactant complex and transition state structures using single-point calculations in Gaussian-09,^65^ with the 6-311+G* basis set on all atoms on the optimized geometries and transition states. The field direction and magnitude are defined as in Gaussian. In addition to quantum mechanical electric field calculations, we also estimated the electric field gradient using the Poisson–Boltzmann equation on the vanadate-bound protein pdb file with a charge of −1 placed in the position of the vanadium ion representing the V(O)_2_(OH)_2_ reactant state. All protein charges assume pH 7.5 conditions, while His_404_ is doubly protonated and His_496_ fully neutral. In particular, the charges from only the protein atoms were used to estimate the electric field magnitude and direction as the negative gradient of the electrostatic potential calculated using the Finite Difference Poisson–Boltzmann equation.^77,78^

### Quantum Mechanics/Molecular Mechanics (QM/MM) Calculations

Using the last snapshot from the MD simulation on 2-phenylindole a QM/MM calculation was performed using the ONIOM approach as implemented in Gaussian-09.^65^ Hypohalide was added to the structure by modifying a water molecule near the carbon acceptor on the 2-phenylindole. The QM region was described by the substrate, HOCl, and four water molecules as well as an active site glutamine Gln_220_ side-chain hydrogen-bonded to the HOCl group (Supporting Information Figure S29) and calculated with density functional theory using UB3LYP with a 6-31G* basis set.^66,67,70^ The MM region was described as above with the Amber force field, while the link-atom approach applied to the borders between the QM and MM regions and electronic embedding was used for the QM/MM electrostatic interactions.

## Results and Discussion

### Role of Substrate Binding in the *Ci*VCPO Enzymatic Reaction

As vanadium chloroperoxidase enzymes are known to halogenate aromatic rings of lignin structures in nature,^35^ we chose indole and indole derivatives as substrates for our work. Furthermore, the indole analogues, i.e., 2-phenylindole and methylphenylindole, have fluorescent properties that, unlike indole, do not overlap with the signal derived from tryptophan residues. In addition, previous studies have suggested fluorescence quenching upon the addition of 2-phenylindole to *Ci*VCPO.^54^ Selectivity studies have also shown that indoles and terpenes are brominated preferentially over monochlorodimedone, when present in equimolar concentrations,^12^ providing further alignment with the possibility of an enzyme–substrate complex that reacts with the HOX intermediate. To investigate the hypothesis that an enzyme–substrate complex is essential for catalysis and to understand whether substrate binding occurs prior to or after the generation of HOX and how this affects the catalytic cycle, we performed a joint experimental and computational approach.

**Figure 2:**
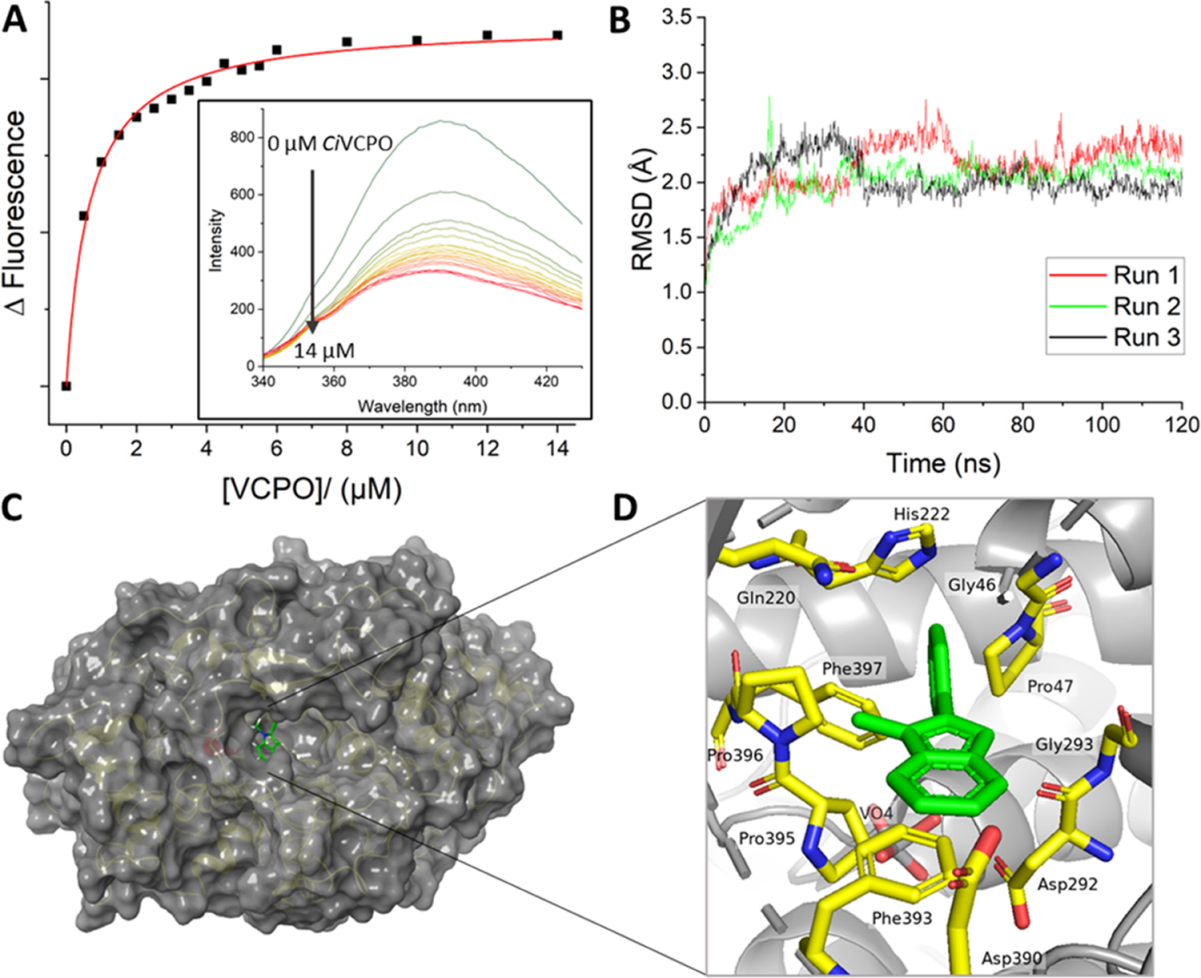
Binding of methylphenylindole to *Ci*VCPO. (A) Fluorescence binding titration of methylphenylindole (0.5 μM) to *Ci*VCPO at pH 7.0. The difference in fluorescence emission between 380 and 400 nm has been fitted to a tight-binding equation to give a dissociation constant, *K*_D_, of 0.66 ± 0.046 μM. The fluorescence emission spectra of methylphenylindole at different *Ci*VCPO concentrations are shown in the inset upon excitation at 316 nm. (B) Molecular dynamics simulations showing the RMSD (root-mean-square deviation) of methylphenylindole in the suggested binding pocket of *Ci*VCPO. (C) Binding site of methylphenylindole at the entrance of the protein tunnel in *Ci*VCPO after docking. The close-up (D) shows how the methylphenylindole (green) interacts with residues in the binding pocket (carbon in yellow, oxygen in red, and nitrogen in blue).

Initial fluorescence binding titrations with methylphenylindole show a decrease in intensity of the fluorescence emission spectra as the *Ci*VCPO concentration is increased (Figure 2A inset), in agreement with the fluorescence quenching observed by Butler et al using an *Ascophyllum nodosum* VBPO in a reaction with 2-phenylindole.^54^ Our obtained fluorescence changes were plotted as a function of enzyme concentration and fitted to a tight-binding model to give a dissociation constant, *K*_D_, of 0.66 ± 0.05 μM, for methylphenylindole binding to *Ci*VCPO enzyme. This *K*_D_ value gives evidence of the fact that the substrate binds to the enzyme strongly and most likely into a specific binding site inside the protein structure.

To characterize the substrate-binding pocket, we ran a series of molecular mechanics (MM) and molecular dynamics (MD) simulations of the protein and substrate interactions. Initially, molecular docking studies were performed to identify a putative substrate-binding site of indoles into the vanadium haloperoxidase protein by using the vanadate-bound crystal structure coordinates of *Ci*VCPO (PDB entry 1IDQ). Previous work using the Deepsite approach substrate binding pockets were predicted in the VCPO enzyme structure,^50^ which matches the location of the substrate binding predicted by the docking excellently. The highest-scoring structure for methylphenylindole bound to *Ci*VCPO was subsequently subjected to a series of molecular dynamics simulations of 110 ns each to test the stability and flexibility of the substrate-bound orientation to the enzyme. The root-mean-square deviation plot shows that the methylphenylindole substrate remains bound during each of the three molecular dynamics runs over 110 ns (Figure 2B). In particular, the methylphenylindole is bound to *Ci*VCPO in a small but open pocket of the protein at a distance of 11.2 Å from the vanadate cofactor active site (Figure 2C). The short distance between the vanadium cofactor and the substrate implies that the HOX intermediate formed at the vanadate cofactor will not have to travel far to react with the substrate. This will reduce the likelihood of the HOX reacting with alternative groups such as the Trp residues of the protein. A similar mode of binding was also observed for 2-phenylindole and indole with the same protein residues involved in the binding of all three substrates (Figures S3 and S4, Supporting Information). Furthermore, longer molecular dynamics runs of 500 ns were performed for 2-phenylindole and methylphenylindole (Supporting Information Figure S5) showing some movement of the substrates within the binding pocket close to the surface. These simulations highlight that despite some degree of substrate mobility, they remain close to the end of the tunnel, where the vanadate cofactor is placed; thereby enabling a reaction with HOX released from that tunnel.

Molecular dynamics simulations for 500 ns each were performed to test the stability of the halogenated products, namely, 2-phenyl-3-chloroindole and 2-phenyl-3-bromoindole, in the substrate binding pocket (see Supporting Information Figure S6). Despite prolonged stability in some simulations where the products remain in the substrate binding pocket, the 2-phenyl-3-bromoindole is also observed dissociating from the binding pocket entirely.

The protein residues in the proximity of the 2-phenylindole substrate in the substrate–enzyme complex are shown in Figure 2D and include the amino acids Pro_47_, His_222_, Gln_220_, Asp_292_, Gly_293_, Asp_390_, Phe_393_ Pro_396_, and Phe_397_. These results suggest that VHPO enzymes have a distinct substrate-binding pocket near the protein surface for favorable substrate binding. Alternative substrates, including erythromycin, styrene, monochlorodimedone, geraniol, and cytosine were also investigated for binding the same protein (Figures S7–S11, Supporting Information). Interestingly, these substrates are bound in the same area as that located for methylphenylindole. As such, VHPO enzymes have a distinct substrate-binding pocket that can accommodate a range of substrates with various shapes and sizes.

Previous work on vanadium haloperoxidases has shown that the first stage of the catalytic cycle involves its reaction with H_2_O_2_ and halogen to form the HOX intermediate at the vanadate active site.^50^ In the absence of bound substrate, the HOX is then expected to travel through a protein tunnel that connects the vanadate pocket with the surface to be released into solution to react with potential substrates.^79^ However, this would contradict the selectivity previously observed for VCPO enzymes.^26,54^ Moreover, the presence of a distinct binding site at the entrance of a small tunnel would suggest that the enzyme may actually assist with the second step of the reaction by providing a reaction site for the reaction of HOX with substrate. As such HOX is not released into the solution but stays in the protein tunnel, where it reacts with substrate.

**Figure 3:**
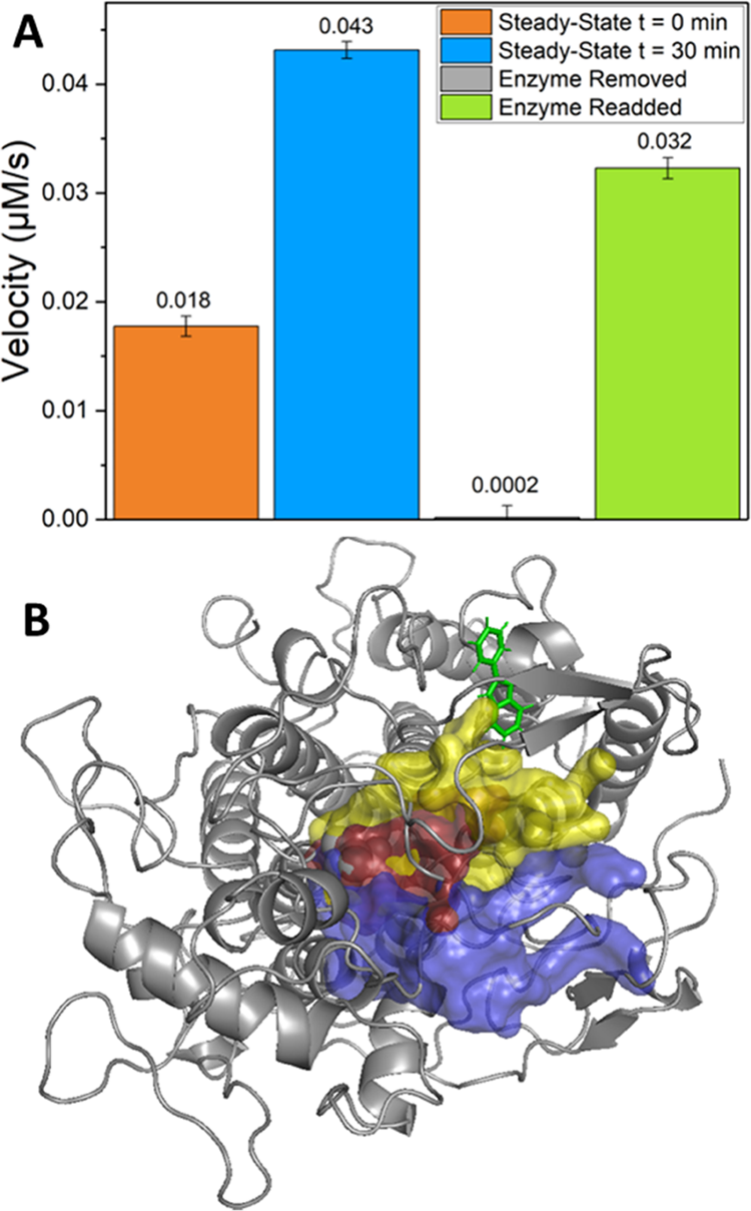
Activity of *Ci*VCPO toward 2-phenylindole with different halogens. (A) Bar chart showing the rate of bromination of 2-phenylindole under steady-state conditions upon either: (i) initiating catalysis with the addition of *Ci*VCPO (orange bar); (ii) pre-mixing *Ci*VCPO with H_2_O_2_ and KBr for 30 min prior to addition of 2-phenylindole (blue bar); (iii) removing the enzyme from (ii) by filtration or denatured by heat shock prior to the addition of 2-phenylindole (gray bar); (iv) re-adding *Ci*VCPO to the sample from (iii) (green bar). (B) Tunnel analysis of 2-phenylindole (in green) MD simulation using the Caver software package showing the tunnels (red, yellow, and blue) linking the vanadate active site to the substrate binding pocket.

To test this hypothesis, we performed several activity measurements with and without protein by measuring the decrease in the fluorescence of 2-phenylindole and methylphenylindole as a probe for the halogenation chemistry (Figure 3A). Four specific experiments were performed. First, H_2_O_2_, KBr, and substrate were mixed, and the reaction was triggered by adding enzyme, designated “Steady-State *t* = 0 min” in Figure 3A. In a second experiment, designated “Steady-state *t* = 30 min,” the enzyme, H_2_O_2_, and KBr were pre-mixed for 30 min and afterward substrate was added. In a third experiment, designated “enzyme removed,” the enzyme, H_2_O_2_, and KBr were pre-mixed for 30 min; however, thereafter enzyme was removed through filtration before substrate was added. In a final experiment, designated “enzyme re-added” in Figure 3A, the mixture obtained from the third experiment, i.e., the one labeled as “enzyme removed,” was subjected to a new enzyme again. All rates have been compared to those obtained under standard steady-state conditions, where assays included 2-phenylindole, KBr, and H_2_O_2_ and were initiated with the addition of enzyme (steady state at *t* = 0 min, orange column in Figure 3A). As can be seen from Figure 3A dramatic differences in reaction rates are observed for the four individual experiments. When the enzyme is first allowed to react with KBr and H_2_O_2_ prior to initiating the reaction with the addition of 2-phenylindole (Steady-state *t* = 30 min, blue column in Figure 3A) the rate of substrate bromination is significantly faster (*k*_obs_ = 0.043 μM s^–1^) than when there is no pre-mixing, despite the known formation of dioxygen that occurs in the system in the absence of substrate.^80,81^ These studies imply that the accumulation of the HOBr intermediate in the protein speeds up catalysis. Furthermore, it implicates that HOBr formation will be partially rate-limiting in the overall process. Conversely, if the enzyme is removed following HOBr accumulation (“enzyme removed” column in Figure 3A) no reaction is observed, which is also the case at different pH values ranging from 5.5 to 10. These experiments, therefore, confirm that HOBr is not able to react directly with the substrate in solution. This is further supported by the lack of substrate halogenation when NaOCl was mixed directly with 2-phenylindole or methylphenylindole in the absence of *Ci*VCPO.

We then added fresh enzyme back to the sample labeled under “enzyme removed” where HOBr had been allowed to accumulate (i.e., the initial enzyme had been removed). Quickly after the addition of enzyme, an increase in the rate of conversion of 2-phenylindole to brominated 2-phenylindole is observed (green column, Figure 3A). Based on these findings, it is therefore clear that the presence of the enzyme (and thus presumably substrate binding) is essential for the halogenation reaction to take place. However, substrate binding is not likely to be rate-limiting for catalysis.

As the experimental work highlights the importance of substrate binding to the protein, we investigated the substrate-binding pocket in detail to search for any tunnels or pathways that may connect the vanadate cofactor to the substrate-bound region. We followed up the docking results with additional MD simulations on the enzyme–substrate complex. All MD frames from the MD simulation were made available to the Caver software package^82^ to identify tunnels linking the vanadate active site with the substrate-binding pocket close to the surface of the protein (Figures 3B and S12, Supporting Information). Three relatively small and narrow tunnels (highlighted in red, yellow, and blue), which may accommodate small molecules, like H_2_O_2_, halide, hypohalide, water molecules, and protons, were identified. However, none of the tunnels are large enough to fit indole or substituted indoles. Therefore, substrate entrance into the vanadate-bound active site is ruled out from the calculations. Interestingly, the yellow tunnel connects the vanadate cofactor of VHPO with the substrate-binding site and may provide a route for the HOX to travel from the vanadate active site to the substrate and trigger a chemical reaction for halide transfer. Taken together with the experimental data we conclude that vanadate reacts with H_2_O_2_, halide, and protons to form hypohalide in an initial reaction cycle, whereby the hypohalide is subsequently ejected from the vanadate-bound region and shuttled toward the substrate-binding pocket where it reacts with substrate. In addition, umbrella sampling molecular dynamics studies were performed on HOCl movement through the tunnel connecting the vanadate cofactor and the substrate-binding site (see Supporting Information Figure S13). These simulations show a gradual movement of HOCl through the tunnel and implicate a low energy barrier for HOCl release from the vanadate active site followed by energetically favorable movement toward the substrate binding pocket.

### Different Catalytic Steps Limit Reaction Chemistry with Different Halogens

As our initial fluorescence studies unambiguously confirm that the reaction of the HOX intermediate with aromatic substrates is only catalyzed in the presence of the enzyme, we decided to follow up our work with a kinetics study and identify the rate-limiting steps during catalysis. To this end, steady-state activity measurements and stopped-flow absorption kinetics experiments were carried out to establish what drives the reaction and which steps are likely to be rate-limiting during catalysis.

**Figure 4:**
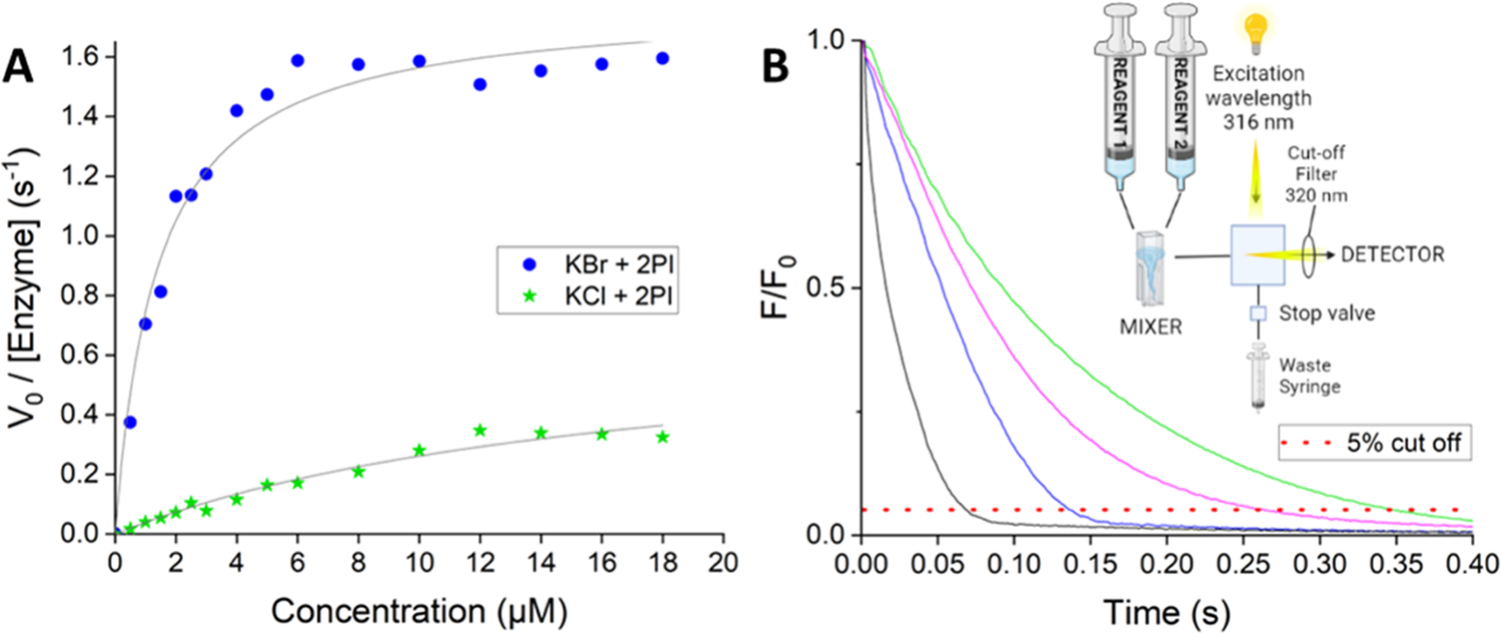
Kinetics of 2-phenylindole halogenation catalyzed by *Ci*VCPO. (A) Michaelis–Menten analysis of the steady-state kinetics following the reactions of 2-phenylindole with KBr (blue dots) or KCl (green stars) by *Ci*VCPO. The black lines show the fit to the Michaelis–Menten equation. (B) Examples of stopped-flow kinetic transients showing the decrease in fluorescence upon halogenation of 2-phenylindole with either Br^–^ (black and blue) or Cl^–^ (magenta and green). Transients are shown upon mixing [*Ci*VCPO] with [2-phenylindole + H_2_O_2_ + halogen] (blue and green traces) or [*Ci*VCPO + H_2_O_2_] with [2-phenylindole + halogen] (black and magenta traces). The red line indicates the point at which the fluorescence has decreased by 95%. The inset shows a schematic of the stopped-flow setup used for these measurements. The *R*_95%_ values (see main text) are given in Table 1.

Steady-state activity measurements with indole derivatives (2-phenylindole or methylphenylindole) follow Michaelis–Menten (saturation) behavior irrespective of whether Br^–^ or Cl^–^ is used (Figures 4A and S14–S17, Supporting Information). This is consistent with our hypothesis above that the enzyme is essential for catalyzing the halogenation of the substrates. Even though 2-phenylindole and methylphenylindole both yield similar kinetic parameters for *K*_m_ and *k*_cat_ (see Table S3, Supporting Information), significant differences are observed between the bromination and chlorination chemistry catalyzed by *Ci*VCPO. The *k*_cat_ value for the bromination reaction was determined to be 1.64 s^–1^ for methylphenylindole and 1.74 s^–1^ for 2-phenylindole. These values are about 3-fold higher than those obtained for the equivalent chlorination chemistry, namely, 0.68 s^–1^ for methylphenylindole and 0.63 s^–1^ for 2-phenyindole. Moreover, the *K*_m_ values for the indole substrates increase significantly for the chlorination reaction compared to the bromination chemistry. The *K*_m_ values (1.58 μM for methylphenylindole and 1.52 μM for 2-phenylindole) for bromination are comparable to the *K*_D_ value from the fluorescence binding titration (0.66 μM). In contrast, the *K*_m_ increases to >15 μM (accurate values could not be determined due to the insolubility of the substrates at higher concentrations) in the presence of KCl, suggesting that the rate-limiting step may be different for each halogen.

To probe the possible rate-limiting steps in more detail, we used stopped-flow kinetics experiments to follow the halogenation of 2-phenylindole in single-turnover measurements under different mixing regimes for the insertion of either Br^–^ or Cl^–^ (see Table 1). Due to the complex multisubstrate and multistep nature of the reaction, it was not possible to fit any of the kinetic stopped-flow fluorescence transients to a single- or multiexponential function to obtain an observed rate constant (Figures S20 and S21, Supporting Information). Consequently, we have analyzed the data qualitatively by calculating the time taken to achieve 95% product formation: designated *R*_95%_ (i.e., for the fluorescence to decrease by 95%, Figure 4B).

**Table 1:** Stopped-Flow UV/Vis Spectroscopy Measurements Following the Observed Reaction Times (*R*_95%_) for Halogenation of 2-Phenylindole When Mixing Components of Syringe 1 with Syringe 2^a^

entry	syringe 1	syringe 2	*R*_95%_ using KBr (s)	*R*_95%_ using KCl (s)
1	*Ci*VCPO	SUB, KX, H_2_O_2_	0.137 ± 0.001	0.348 ± 0.009
2	*Ci*VCPO, SUB	KX, H_2_O_2_	0.138 ± 0.001	0.344 ± 0.012
3	*Ci*VCPO, H_2_O_2_	SUB, KX	0.069 ± 0.001	0.264 ± 0.009
4	*Ci*VCPO, H_2_O_2_, KX	SUB	0.022 ± 0.001	0.087 ± 0.003

a X = Br or Cl, SUB = 2-phenylindole.

Four experiments were done for bromination and chlorination of 2-phenylindole in *Ci*VHPO. First, substrate, H_2_O_2_, and KX were mixed and then added to an enzyme solution. In the second experiment, substrate and enzyme were mixed and later a mixture of H_2_O_2_ and KX was added. In the third experiment, the enzyme was mixed with H_2_O_2_ and afterward KX and substrate were supplied. Finally, an experiment was done with pre-mixed enzyme, H_2_O_2_, and KX, and later, the substrate was added.

Similar to the steady-state measurements, the apparent rate of bromination chemistry is considerably faster than the chlorination reaction under all conditions. Moreover, the apparent rate of halogenation is almost identical when the enzyme is mixed against all three reactants, namely, H_2_O_2_, halogen, and 2-phenylindole, compared to pre-binding of the substrate prior to mixing with HX and H_2_O_2_ (entries 1 versus 2 in Table 1). These results provide further evidence that substrate binding is not rate-limiting during the catalytic cycle.

In contrast, when the enzyme is mixed with H_2_O_2_ first (entry 3, Table 1) prior to exposure to substrate and KX, the apparent rate is significantly faster than when H_2_O_2_ is added later (compare the results of entries 1 and 2 with entry 3 in Table 1). This is more pronounced for Br^–^ than it is for Cl^–^, whereby the *R*_95%_ drops by almost 50% in value. A further drop in the *R*_95%_ value is seen when the enzyme is pre-mixed with H_2_O_2_ and KX prior to mixing with the substrate (entry 4, Table 1), again confirming that substrate halogenation by HOX is not rate-limiting. A similar effect is observed when methylphenylindole is used as a substrate, although it should be noted that there is a slight increase in the rate of reaction when the substrate is pre-bound in this case (Figures S20 and S21, Supporting Information). These single-turnover stopped-flow experiments therefore highlight that the reaction of H_2_O_2_ and halide with the vanadate cofactor to form hypohalide is likely to be the rate-limiting step in the overall reaction cycle. However, as the rate of substrate chlorination is slower than that of bromination it indicates that different catalytic steps may be (partially) rate-limiting for different halogens. The experimental findings from the current work align with computational studies from the literature,^50,83^ where the highest energy barrier in the formation of HOCl is the transfer of OH^+^ to the chloride. However, OH^+^ transfer to the bromide is likely to be less energetically costly and an earlier energy barrier for the binding and activation of H_2_O_2_ at the *Ci*VCPO vanadate cofactor may be rate-limiting for the formation of HOBr.

### Mechanism of Substrate Halogenation by VCPO

**Scheme 1:**
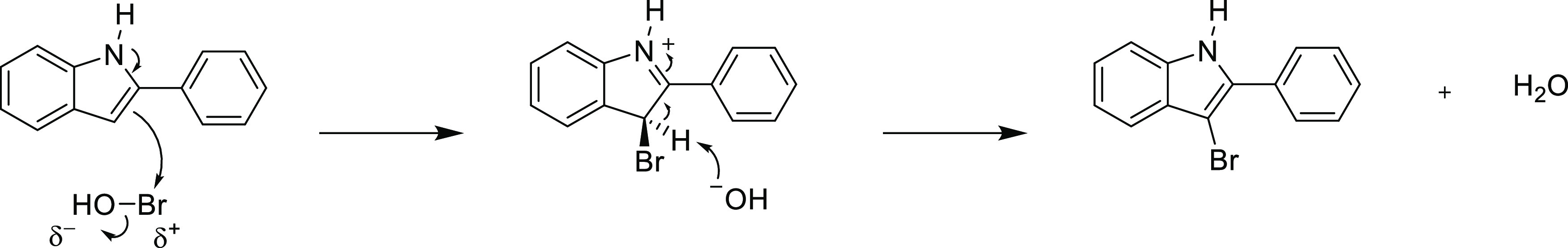
Proposed Halogenation Mechanism of 2-Phenylindole with HOBr in *Ci*VCPO

To understand why the enzyme is essential for the halogenation of the indole derivatives, we investigated the mechanism of substrate halogenation in more detail by using a combination of analytical and computational methods. Product analysis (Supporting Information Figures S22 and S23) confirms that the indole derivatives are singly halogenated at the unsubstituted carbon on the 5-membered ring (see Scheme 1) most likely through a nucleophilic addition mechanism. As the aromaticity of the phenyl and benzene rings is unlikely to be easily broken at room temperature, the carbon on the 5-membered ring is the only possible position for C–X bond formation. NMR data also reveals that the proton at this position is removed (Supporting Information Figure S24). As shown in Scheme 1, we predict that the reactivity of the HOX oxidants toward the indole derivatives is likely to be driven by partial charge separation across the HO–X bond caused by the electronegativity of the oxygen atom. This charge separation is more pronounced in HOBr than HOCl due to the electronegativity difference of chlorine versus bromine. It is also tempting to speculate that this becomes further enhanced in the enzyme tunnel via H-bonding interactions with surrounding residues, thus explaining why the enzyme is necessary to catalyze this step in the reaction.

**Figure 5:**
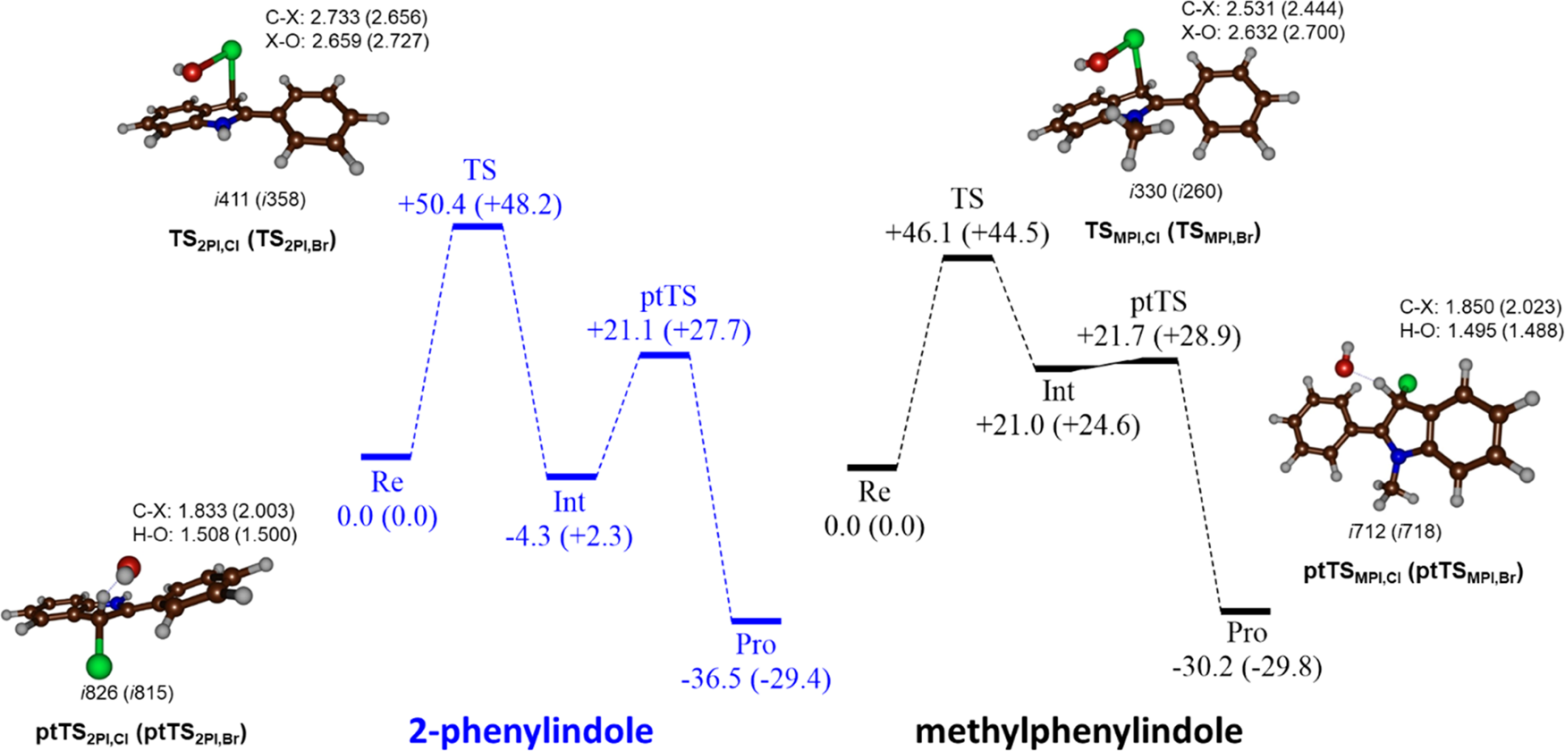
Energy profiles and optimized geometries calculated with implicit solvent model included for the reactions of the indole derivatives with HOX. Data for 2-phenylindole (left) in blue. Data for methylphenylindole (right) in black. The structures show bond lengths in Å and the imaginary frequency of the transition state in cm^–1^, while the energies are calculated at UB3LYP/BS2//UB3LYP/BS1 and contain ZPE and solvent corrections in kcal mol^–1^.

Subsequently, DFT methods were applied to investigate the reaction mechanism of substrate halogenation and the possible effects of the enzyme environment on the energetic barriers of the C–X bond formation of methylphenylindole and 2-phenylindole by HOCl/HOBr. Calculations were carried out in the gas phase as well as using a continuum-polarized conductor model (CPCM, see Tables S5–S20, Supporting Information). In general, adding a solvent model lowers the barriers by 3–5 kcal mol^–1^ but gives the same trends and similar optimized geometries. The overall landscape using an implicit solvent model is shown in Figure 5 for the reaction mechanisms of chlorination and bromination of 2-phenylindole (Figure 5, blue) and methylphenylindole (Figure 5, black). The reactions start from a reactant complex of HOX with the substrate (**Re**_2PI_ for 2-phenylindole and **Re**_MPI_ for methylphenylindole) and proceed via a transition state for C–X bond formation (**TS**_2PI_ and **TS**_MPI_). Thereafter, the system relaxes to a local minimum with halide bound indole (**Int**) and, through proton transfer from the *ipso*-carbon position to OH^–^ via transition state **ptTS**, leads to halogenated products (**Pro**). The electrophilic addition step is rate-determining for all systems and a much smaller proton transfer barrier is found in all cases.

Nevertheless, the overall reaction is highly exothermic for all substrates and halides by 44.5–50.4 kcal mol^–1^. Despite this, high halogen transfer barriers are calculated for all substrates and hypohalides. These results confirm the experimental work that no products are obtained from mixing HOX with indoles in solution. Therefore, the computational results highlight that substrate halogenation must take place in the enzyme (or on the enzyme surface), which must lower the halogen transfer reaction barriers dramatically with respect to those in solution. *Ci*VCPO might have a catalytic role in this step of the reaction by stabilizing the reactants by binding and potentially lowering the energy barriers. This is supported by the low pH optimum for the enzyme activity at pH 5.5 (see Supporting Information Figure S25), where the majority of residues, such as Arg, His, and Lys are in their protonated states. The polar residues in the protein are, therefore, likely to play a role in forming hydrogen-bonding interactions with the HOX intermediate as it accumulates.

As the p*K*_a_ of HOCl and HOBr are ∼7.5 and ∼8.7, respectively, the hydrogen-bonding interactions with protein residues would allow for the OH component of the HOX intermediate to have a more negative partial charge, thus increasing the positive charge of the halogen and lowering the energy barrier of the C–X bond formation. Inspection of the residue types present around the entrance to the protein hydrogen peroxide and halogen tunnel shows a large number of polar residues (green area around the 2-phenylindole in orange in Figure S26). Interestingly, mass spectrometry analysis of a reaction of VCPO with H_2_O_2_ and KCl in the absence of substrate gives an increase in the mass of the enzyme, which implies that HOX can react with enzymatic amino acid residues possibly located in the substrate entrance tunnel (Figure S27). For both substrates, the energy barrier of bromination of the indole derivatives is slightly lower than that for chlorination (+48.2 vs +50.4 kcal mol^–1^ for 2-phenylindole and +44.5 vs +46.1 kcal mol^–1^ for methylphenylindole), supporting the findings that bromination is faster than chlorination.

Optimized geometries for the transition states with HOCl are shown in Figure 5, while those with HOBr are given in the Supporting Information (Figure S28). In the electrophilic transition state **TS**_2PI_, the O–Cl bond elongates to 2.76 Å, while the C–Cl bond remains relatively long at 2.84 Å and consequently represents an early transition state. By contrast, the transition state is much later on the potential energy profile with methylphenylindole as a substrate with an O–Cl distance of 2.63 Å and a C–Cl distance of 2.53 Å in **TS**_MPI_. The imaginary frequencies for **TS**_2PI_ and **TS**_MPI_ have values of i256 and i330 cm^–1^, respectively, and represent a C–Cl stretch vibration. Surprisingly, the proton transfer transition states have small imaginary frequencies of i277 cm^–1^ for **ptTS**_2PI_ and i243 cm^–1^ for **ptTS**_MPI_. Typical proton or hydrogen transfer barriers are narrow and steep, and usually these transition states have an imaginary frequency of well over i1200 cm^–1^.^84,85^ Structurally, the proton transfer transition states are early with long H–O distances of (1.74 and 1.71 Å for **ptTS**_2PI_ and **ptTS**_MPI_) and short C–H distances.

**Figure 6:**
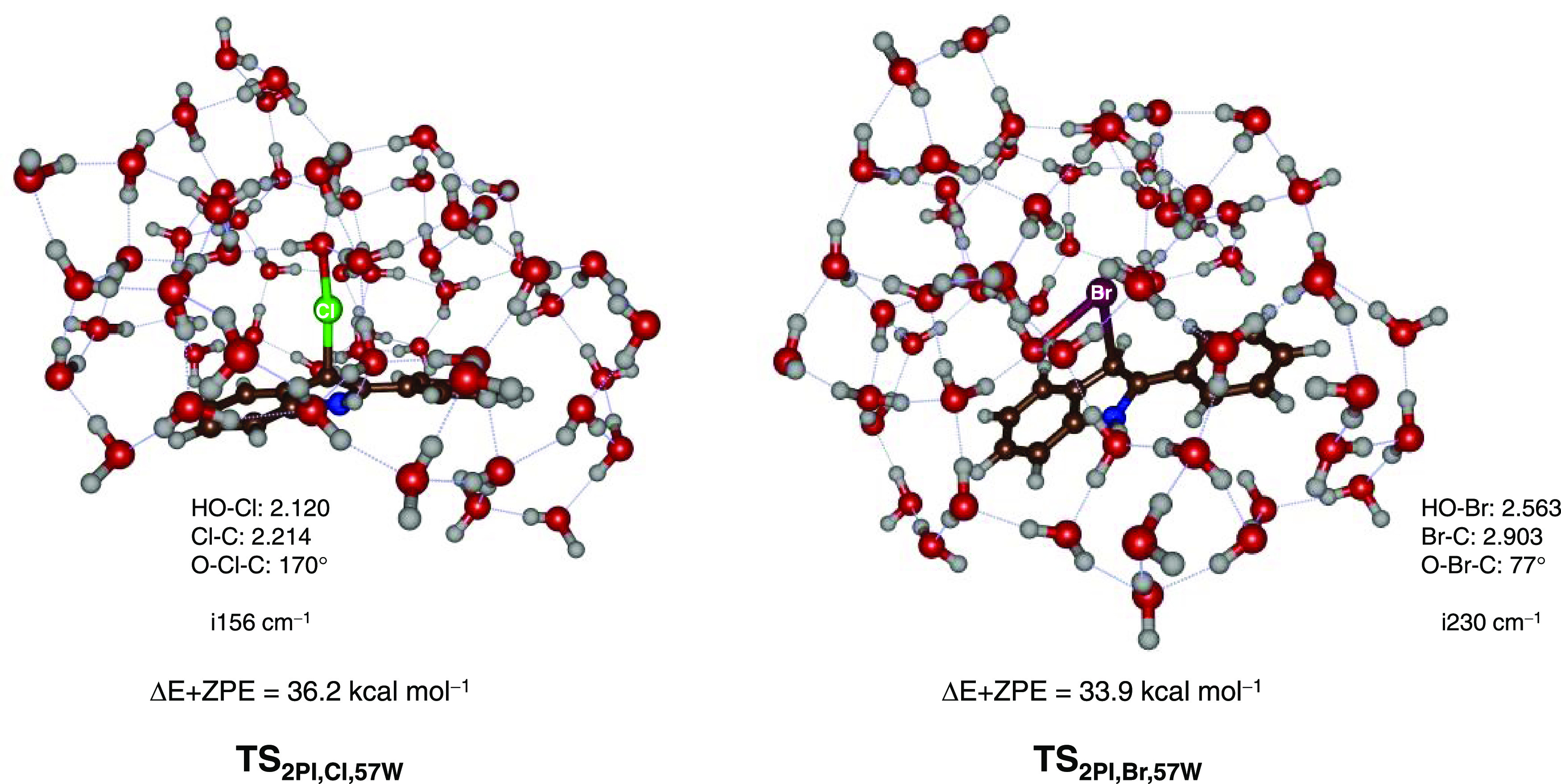
Optimized (UB3LYP/BS1) geometries of the halide transfer barriers from HOCl (left) and HOBr (right) to 2-phenylindole. Bond lengths are in angstroms, angles in degrees, and the imaginary frequency of the transition state in cm^–1^. Barriers are Δ*E* + ZPE values obtained at UB3LYP/BS2//UB3LYP/BS1 in kcal mol^–1^.

To understand how the barriers for halide transfer could potentially be reduced we calculated additional models with explicit solvent water molecules included. In particular, models were created with 57 water molecules surrounding the cluster of HOX (X = Cl, Br) and 2-phenylindole (Figure 6 and Supporting Information Tables S21–S26). In this case, the halide transfer barriers are reduced to 36.2 kcal mol^–1^ (for HOCl) and 33.9 kcal mol^–1^ (for HOBr). However, these barriers are still considerable and will be kinetically demanding under room temperature conditions. The cluster calculations further confirm that a water solvent does not produce a polar enough environment to guide a halide transfer reaction from hypohalide to 2-phenylindole and that an enzyme–substrate complex is needed for catalysis.

Geometrically, adding the water cluster leads to considerably shorter distances than those found in the gas-phase models even when an implicit solvent model is used in the calculations. Thus, the water cluster transition state for Cl transfer (**TS**_2PI,Cl,57W_) has an almost linear conformation with a C–Cl–O angle of 170° and C–Cl and Cl–O distances of 2.214 and 2.120 Å, respectively. By contrast, the bromination transition state is more bent with a C–Br–O angle of only 77°, which is more in line with the gas-phase structure. The O–Br distance is slightly shorter than in the gas phase at 2.563 Å, while the C–Br distance is elongated to 2.903 Å.

Exploratory QM/MM calculations were run and a geometry optimization was performed at the QM(UB3LYP/6-31G*:Amber) level of theory on HOCl approaching 2-phenylindole in the substrate binding pocket of VCPO. These calculations confirm a similar approach as that seen in the water-bound models depicted in Figure 6, see Supporting Information Figure S29.

**Figure 7:**
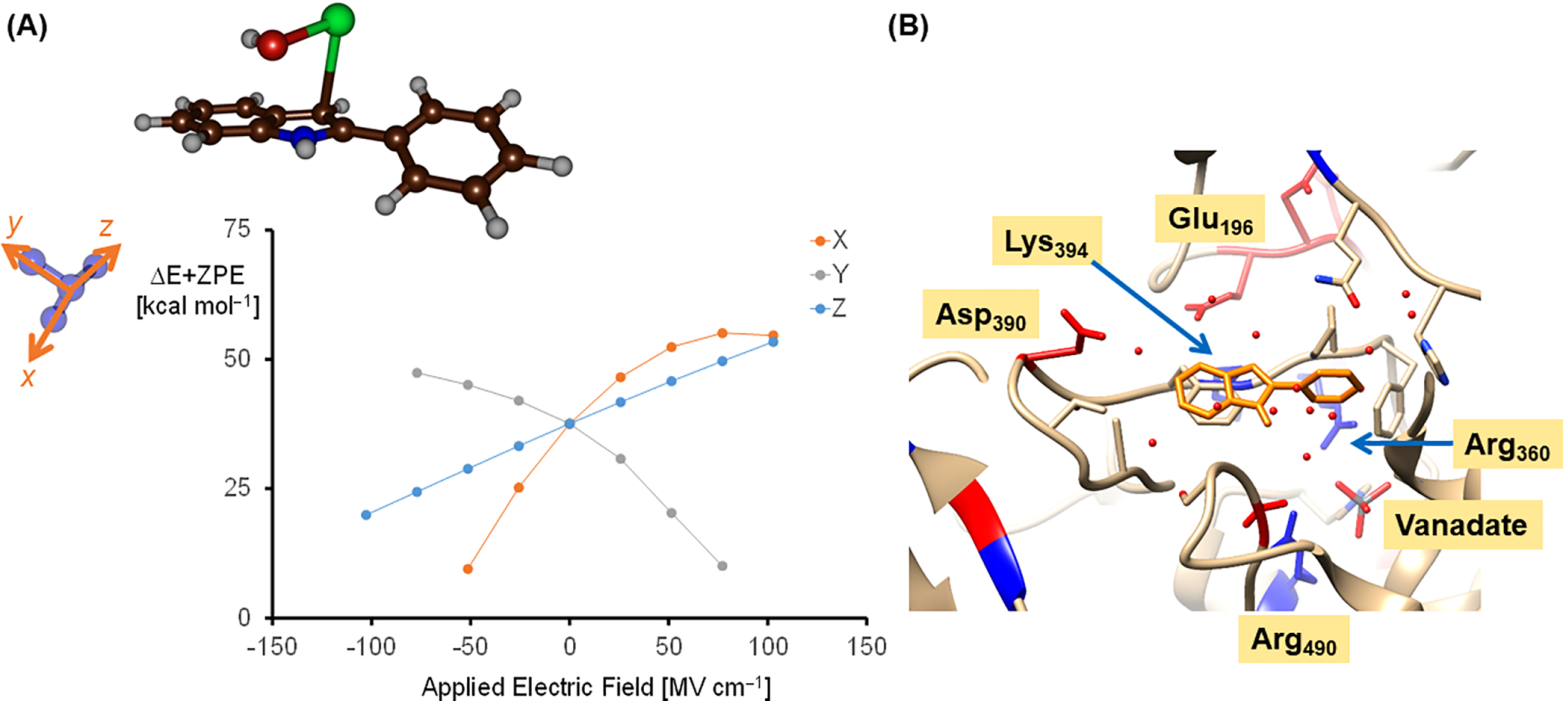
(A) Electric field effect calculations on the halogen transfer barrier from HOCl to 2-phenylindole as calculated at the UB3LYP/BS2 level of theory. The positive axis is as defined in Gaussian-09, and its direction is given with respect to the transition state structure. (B) Local environment of the substrate-binding orientation as obtained from the last frame of the docking simulation using methylphenylindole as a substrate with Asp residues highlighted in red, Lys residues in blue, polar residues in light blue, and hydrophobic residues in green.

To gain insight into polarization effects on the halogen transfer reaction and particularly in a polar protein environment, we tested applying an electric field effect along the molecular, *x*,-, *y*-, or *z*-axis on the transition states. To this end, we took the implicit solvent-optimized structures for the halogen transfer transition states and reactants and carried out single-point calculations using an additional electric field effect along a specific direction with specific magnitude. These calculations were previously shown to influence product distributions of enzymatic reactions (i.e., chemo- and regioselectivities), but sometimes also affect the electronic configuration of the oxidant and its properties.^75,76,86−90^ In particular, these field effects influence charge distributions and in the case of HOX with 2-phenylindole the charge separation along the O–X bond. Applying an electric field effect does indeed decrease the energy barriers of the C–X bond formation with the field in a specific direction (see Figure 7A and Supporting Information Figures S30–S32 and Tables S27–S34). In the case of the HOCl with 2-phenylindole reaction, a small negative electric field along the molecular *x*-axis decreases the barrier significantly.

This field is along the C–Cl bond and hence a negative field enhances the C–Cl bond formation step in the reaction by optimizing electron transfer. A field in the opposite direction, by contrast, slows down the reaction dramatically and high energy barriers are obtained. A similar effect is seen along the molecular *y*-axis, which goes through the plane of the indole ring. Along the *y*-axis with positive electric fields, a decrease in halogen transfer barrier is observed, while with negative fields, the barriers are raised. Finally, a steady increase of the barrier is found when the electric field changes from a strongly negative field to a more positive field. The latter field is positioned along the O–Cl bond of HOCl and hence, will push electron density along this bond to trigger a heterolytic cleavage of the O–Cl bond. Overall, the electric field effect calculations show that the halogen transfer barriers are highly sensitive to a polar environment, such as a local electric field or dipole moment that can stabilize and enhance the reactivity.

To investigate how the protein could stabilize and influence the halogen transfer reaction, the structures from the MD simulation were analyzed further. The environment surrounding the substrate in the VHPO structure contains a number of positively and negatively charged residues (Figure 7B). In particular, the vanadate cofactor is surrounded by the positively charged residues Arg_360_, Lys_353_, and Arg_490_ that are located below the aromatic indole ring of the substrate. On the other side of the substrate are located the negatively charged residues of Glu_196_ and Asp_390_. The positioning of these positive and negative residues is likely to create a dipole moment, connecting the vanadate binding site and the substrate binding pocket, which may push the HOX toward the direction of the substrate once formed. Furthermore, the vector connecting the Lys_394_ and Asp_390_ residues is parallel to the C–X bond formation line and hence, the dipole moment and local electric field effect generated by positioning of the Asp_390_ and Lys_394_ residues may assist in the C–X bond formation step. The positioning of these positively and negatively charged residues therefore supports our conclusions from the electric field effect calculations on the gas-phase optimized transition states that electrostatic perturbations in VCPO guide and trigger the halide transfer reaction. Thus, the protein maintains a crucial role in this part of the reaction despite the initial theory that its role stops at the release of the intermediate into solution.

**Figure 8:**
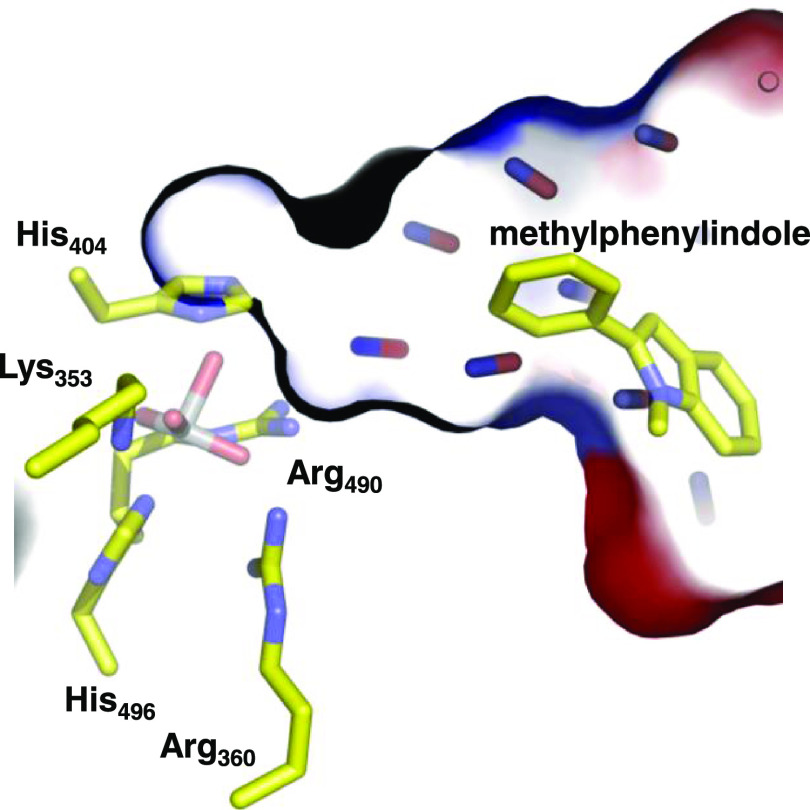
Electric field gradient established in the methylphenylindole-bound protein structure as obtained from the protein charges in the active site. Electric field gradient vectors are displayed as diatomic sticks with blue (more positive) and red (more negative) ends.

To establish further evidence on the local electric field vectors and gradients within the protein, we used the Poisson–Boltzmann approach on the complete protein structure. Previously, these field vectors have led to an understanding of the regio- and chemoselectivity patterns of enzymatic reaction mechanisms.^75,91^Figure 8 shows the electric field gradients in the vanadate and substrate-binding pockets. The positively charged amino acids Lys_353_, Arg_360_, and Arg_490_ are confirmed to trigger a positive field, leading to a gradient pattern from the vanadate cofactor to the substrate binding pocket. These field gradients imply that, after formation at the vanadate center, the HOX is pushed along the electric field vectors of the protein to the substrate binding pocket, where it targets the C3 position. These vectors will be aligned to the C–X bond that is formed and consequently, the local electric field in the protein guides the reaction of HOX with substrate using a low-energy reaction channel through optimum charge separation in the transition state. This field is absent in solution where the charge separation is not enhanced, and hence higher energy barriers are found.

**Scheme 2:**
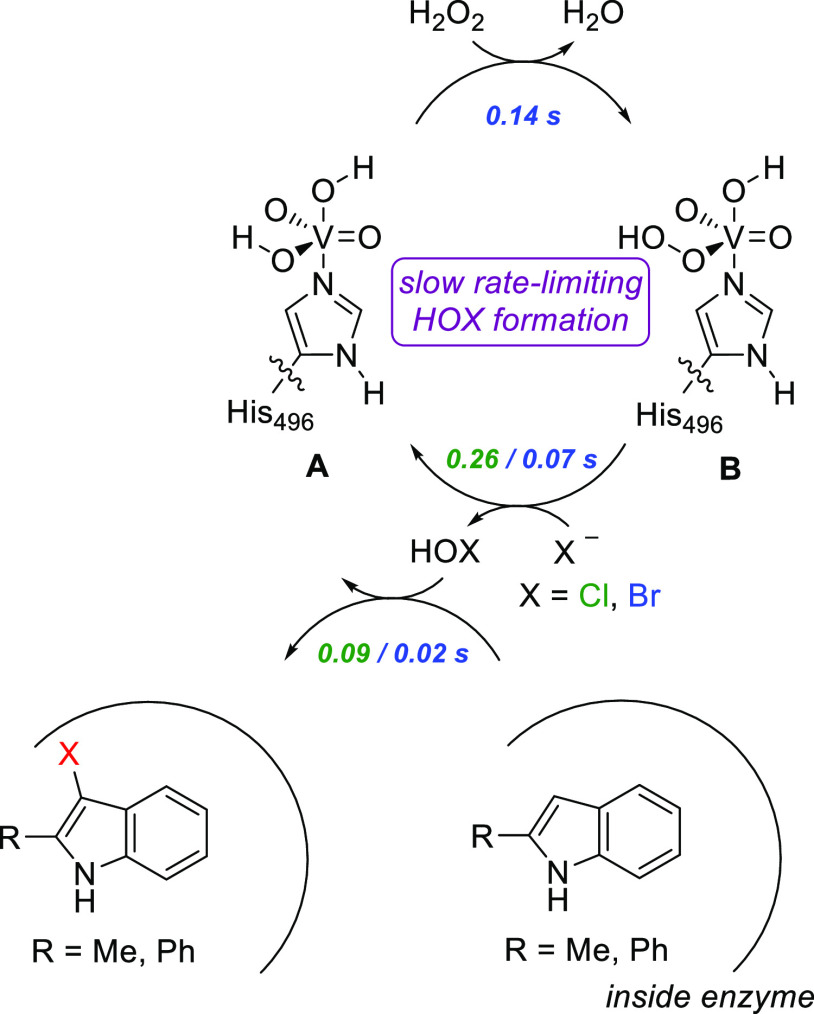
Catalytic Cycle of VHPO Enzymes as Established in This Work. Data Shown Are the Reaction Times Values (*R*_95%_ in s) from Table 1 for X = Cl/Br, and Represent Estimated Upper Limits for the Time Constant for Each Step

A summarizing scheme of the catalytic cycle of VHPO enzymes as derived from the experimental and computational studies described in this work is given in Scheme 2, with the respective time constants providing estimated upper limits for each step from stopped-flow experiments (see Table 1). Thus, starting from a vanadium(V)-dioxo-dihydroxo resting state of the enzyme, the cycle is triggered by H_2_O_2_ binding and activation to form vanadium(V)-dioxo(hydroxo)hydroperoxo species upon release of a water molecule. Although different rates were observed with each halogen for this step in the stopped-flow experiments (see entry 1, Table 1), halide would not be expected to be involved in the chemistry at this stage of the reaction and it implies that a latter step in HOX formation is at least partially rate-limiting for HOCl formation. Consequently, we propose that the time constant for peroxide binding and activation can be derived from the stopped-flow experiments with Br. Subsequently, the vanadium(V)-dioxo(hydroxo)hydroperoxo reacts with a halide to form HOX (X = Cl^–^/Br^–^) and returns the active site to the vanadium(V)-dioxo-dihydroxo species. The HOX, through protein electrostatic interactions, dipole moments, and electric field effects is pulled toward the substrate-binding pocket and is shown to react with 2-methylindole and 2-phenylindole by halogen transfer to the C_4_-position selectively in a fast reaction step. The cycle is completed with product release from the protein. Overall, HOX formation is rate-limiting in the substrate halogenation chemistry, although the rates of HOX formation are different for Cl and Br, and hence, different steps are likely to be partially rate-limiting, at least in the reaction with Cl.

## Conclusions

An extensive combination of experimental and computational techniques has been used to elucidate the role of the *Ci*VCPO enzyme in the halogenation of indole derivatives by the HOX intermediate. The presence of a substrate binding pocket near the protein surface suggests the enzyme may play a catalytic role in the halogenation of substrates beyond the simple generation of HOX. Moreover, a protein tunnel between the vanadate active site and the putative substrate-binding pocket is likely to direct the HOX intermediate toward potential substrates. Activity measurements have confirmed that the enzyme is essential for the halogenation reaction as HOX is not capable of directly halogenating indole derivatives in solution. However, the halogenation of substrates is not rate-limiting. Instead, the initial H_2_O_2_ activation step is at least partially rate-limiting. As this step does not involve HOX, and the apparent rates of turnover are different with HOCl and HOBr, this implies that another HOX-dependent step is also partially rate-limiting, at least in the case of the slower reaction with HOCl.

Moreover, the high energy barriers for substrate halogenation are lowered significantly when an electric field effect is added to the DFT transition states, indicating that the halogen transfer reaction is sensitive to a polar environment and a local electric field strength and direction. Such electric fields have been identified in the *Ci*VCPO enzyme and are aligned with the C–X bond to lower the halogen transfer energy barrier. We expect our results and conclusions to be general for the broad class of vanadium haloperoxidases where we propose substrate to bind in a tunnel nearby the vanadium cofactor that is involved in the hypohalide formation from H_2_O_2_ and halide. The hypohalide then travels through a narrow tunnel toward the substrate binding tunnel where the reaction with the substrate takes place. Overall, our work provides detailed insights into how the protein environment in VHPOs is crucial in catalyzing the substrate halogenation chemistry. This understanding is critical for engineering these important enzymes for improved biocatalytic performance in future biotechnological applications.

## Supporting Information Available

Full data with substrate, product and enzyme characterization, kinetics and stopped-flow results, and full computational data (structures and energies) (PDF)
