# Supplementary material for: How Is Substrate Halogenation Triggered by the Vanadium Haloperoxidase from Curvularia inaequalis?
Source: ACS Catal. 2023 Jun 6;13(12):8247–61. doi: 10.1021/acscatal.3c00761 (PMC10278073; doi:10.1021/acscatal.3c00761)
Supplement: Supplementary file 1 — cs3c00761_si_001.pdf [file cs3c00761_si_001.pdf]

Supporting Information

How is substrate halogenation triggered by the vanadium haloperoxidase from *Curvularia inaequalis*?

Emilie F. Gérard,[§⊥] Thirakorn Mokkawes,[§⊥] Linus O. Johannissen,[§] Jim Warwicker,[§#] Reynard R. Spiess,[§] Christopher F. Blanford,[§‡] Sam Hay,[*§&] Derren J. Heyes,[*§] and Sam P. de Visser[*§⊥]

[§] Manchester Institute of Biotechnology, The University of Manchester, 131 Princess Street, Manchester M1 7DN, United Kingdom

[⊥] Department of Chemical Engineering, The University of Manchester, Oxford Road, Manchester M13 9PL, United Kingdom

[&] Department of Chemistry, The University of Manchester, Oxford Road, Manchester M13 9PL, United Kingdom

[#] School of Biological Sciences, Faculty of Biology, Medicine and Health, The University of Manchester, Oxford Road, Manchester 13 9PL, United Kingdom

[‡] Department of Materials, The University of Manchester, Oxford Road, Manchester M13 9PL, United Kingdom

Corresponding author email addresses: sam.devisser@manchester.ac.uk (SPdV); Derren.Heyes@manchester.ac.uk (DH), Sam.Hay@manchester.ac.uk (SH).

Contents

1. SDS-PAGE 3

2. Fluorescence standard curves 4

3. Vanadate parameters in the topology file 5

4. Molecular dynamics results 7

4.1. RMSD plots 7

4.2. Distance between the vanadium centre and substrates 8

4.1 Hydrogen-bonding 10

5. Ligand docking 11

6. Caver tunnelling analysis 13

7. Umbrella sampling of HOCl in the substrate channel. 14

8. Steady-State UV/Vis spectroscopy 15

8.1. Methylphenylindole 15

8.2. 2-phenylindole 17

8.3. Fluorescence spectra 18

9. Stopped-Flow UV/Vis spectroscopy 19

10. Product analysis 21

10.1. GCMS 21

10.1.1. Methylphenylindole 21

10.1.2. 2-phenylindole 21

10.2. NMR 22

11. pH-dependence of enzyme activity 23

12. Residue types on *Ci*VCPO surface 24

13. Optimized geometries for the transition states for HOBr 25

14. Optimized QM/MM geometry for the HOCl / 2-phenylindole system 25

15. Density Functional Theory (DFT) data 26

16. DFT Calculations with explicit solvent molecules including a water matrix 30

17. Electric Field effects on halogen transfer transition state 32

18. Cartesian Coordinates of DFT models 42

17.1 Implicit solvent model calculations 42

17.2. Explicit solvent model calculations with 57 water molecules 47

1. SDS-PAGE

The SDS-PAGE gel was purchased from Bio-Rad. The loading samples were prepared in a 1:1 ratio of loading dye to protein sample. Each sample was boiled on a heating block (5 minutes, 95 °C). The plates containing the gel were held in the electrode gasket with a buffer dam or another SDS-PAGE. SDS running buffer (0.25 M Tris, 1.92 M glycine, 1% SDS) was added to cover the gel and the wells to a maximum. Unless stated otherwise, each well was loaded with 15 μL loading sample in each well alongside an unstained protein standard (5 μL). The gel was run at 300 Volts for 20 min. Imaging the SDS-Page was done using the Bio-Rad Gel Doc EZ Imager. The molecular weight of *Curvularia Inaequalis* vanadium chloroperoxidase is 67 kDa.

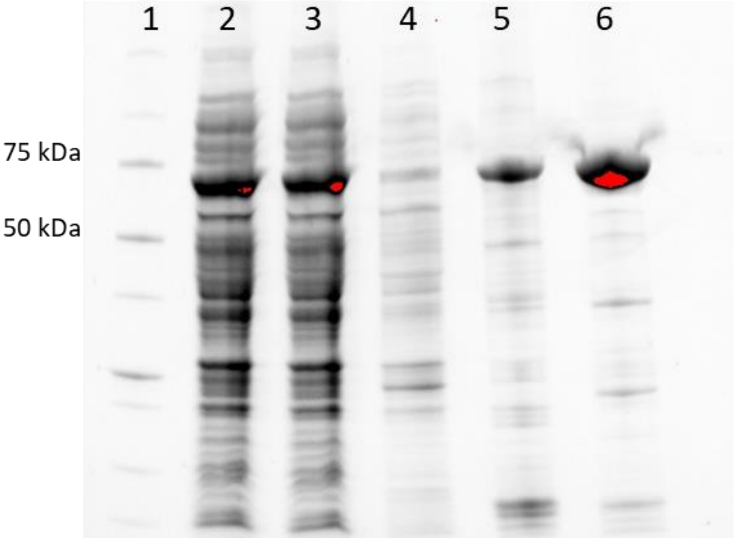

Figure S1. SDS-PAGE analysis of expression of *Ci*VCPO in *E. coli* BL21 competent cells and autoinduction terrific broth at 20°C overnight. Lane 1: protein standard; lane 2: supernatant; lane 3: flow-through; lane 4: wash 1; lane 5: wash 2; lane 6: elution.

2. Fluorescence standard curves

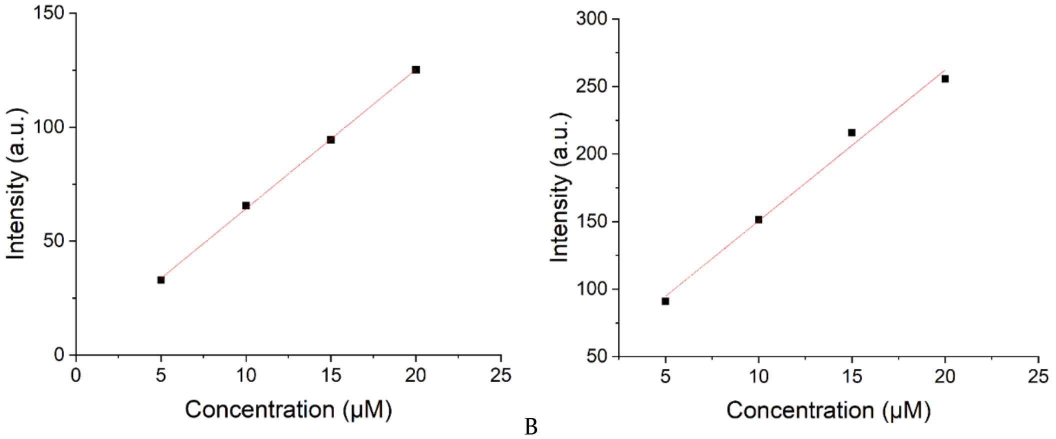

A B

Figure S2. A: Fluorescence standard curve of methylphenylindole in 10% DMSO and MES buffer (50 mM, pH 5.5). The excitation and emission wavelengths were 316 nm and 380 nm, respectively. The data is fit to a linear equation with a slope of 6.1098 Int/μM. B: Fluorescence standard curve of 2-phenylindole in 10% DMSO and MES buffer (50 mM, pH 5.5). The excitation and emission wavelengths were 316 nm and 376 nm, respectively. The data is fit to a linear equation with a slope of 11.173 Int/μM.

3. Vanadate parameters in the topology file

Table 1. Parameters in the topology file for the vanadate bound co-factor.

[atomtypes]					
name	mass	charge	ptype	sigma	epsilon
o	16	0	A	0.2959922	0.87864
V	50.94	0	A	0.2430372	0.044769
oh	16	0	A	0.3066473	0.880314
ho	1.008	0	A	0	0

[molecule type]								
Name	nrexcl							
system1	3							
[atoms]								
residue	493	HIE	rtp	HIE	q	0		
7558	N	493	HID	N	7558	-0.4157	14.01	; qtot -18.415700
7559	H	493	HID	H	7559	0.2719	1.008	; qtot -18.143800
7560	CX	493	HID	CA	7560	0.0188	12.01	; qtot -18.201900
7561	H1	493	HID	HA	7561	0.0881	1.008	; qtot -18.065900
7562	CT	493	HID	CB	7562	-0.0462	12.01	; qtot -18.073300
7563	HC	493	HID	HB2	7563	0.0402	1.008	; qtot -18.036600
7564	HC	493	HID	HB3	7564	0.0402	1.008	; qtot -17.999900
7565	CC	493	HID	CG	7565	-0.0266	12.01	; qtot -17.813100
7566	NA	493	HID	ND1	7566	-0.3811	14.01	; qtot -18.356300
7567	H	493	HID	HD1	7567	0.3649	1.008	; qtot -18.192800
7568	CR	493	HID	CE1	7568	0.2057	12.01	; qtot -18.049300
7569	H5	493	HID	HE1	7569	0.1392	1.008	; qtot -18.328800
7570	NB	493	HID	NE2	7570	-0.5727	14.01	; qtot -17.994900
7571	CV	493	HID	CD2	7571	0.1292	12.01	; qtot -18.215600
7572	H4	493	HID	HD2	7572	0.1147	1.008	; qtot -18.029400
7573	C	493	HID	C	7573	0.5973	12.01	; qtot -17.432100
7574	O	493	HID	O	7574	-0.5679	16	; qtot -18.000000
residue	575	VO4	rtp	VO4	q	-1		
8816	o	575	VO4	O1	8816	-0.46673	16	; qtot -0.466730
8817	V	575	VO4	V1	8817	0.741034	50.94	; qtot 0.274304
8818	o	575	VO4	O3	8818	-0.4696	16	; qtot -0.195296
8819	oh	575	VO4	O4	8819	-0.74141	16	; qtot -0.936706
8820	ho	575	VO4	H1	8820	0.355617	1.008	; qtot -0.581089
8821	oh	575	VO4	O2	8821	-0.78463	16	; qtot -1.365719
8822	ho	575	VO4	H2	8822	0.365714	1.008	; qtot -1.000005
[bonds]								
ai	aj	funct	c0	c1				

7570	8817	1	0.21553	4195354.12				
8817	8818	1	0.164	303534				
8817	8819	1	0.1919	303534				
8817	8821	1	0.1919	303534				
8816	8817	1	0.164	303534				
8821	8822	1	0.0973	332039				
8819	8820	1	0.0973	332039				
[pairs]								
ai	aj	funct						
8820	8821	1						
8819	8822	1						
8818	8820	1						
8818	8822	1						
8816	8820	1						
8816	8822	1						
[angles]								
ai	aj	ak	funct	c0	c1			
8819	8817	8821	1	80.7965344	3644.09664			
8818	8817	8819	1	99.2101426	1857.696			
8818	8817	8821	1	99.2101426	1857.696			
8816	8817	8818	1	110.6780476	3003.10784			
8816	8817	8819	1	99.2101426	1857.696			
8816	8817	8821	1	99.2101426	1857.696			
8817	8819	8820	1	106.6250456	952.44576			
8817	8821	8822	1	106.6250456	952.44576			
7568	7570	8817	1	126.4129	1707.3826			
7571	7570	8817	1	126.4129	1707.3826			
7570	8817	8816	1	91.6597	228.15473			
7570	8817	8818	1	91.6597	228.15473			
7570	8817	8819	1	158.0473	207.595809			
7570	8817	8821	1	77.6342	207.595809			
[dihedrals]								
ai	aj	ak	al	funct	c0	c1	c2	
8820	8819	8817	8821	1	0	6.126771	3	
8819	8817	8821	8822	1	0	6.126771	3	
8818	8817	8819	8820	1	0	15.00661	3	
8818	8817	8821	8822	1	0	15.00661	3	
8816	8817	8819	8820	1	0	15.00661	3	
8816	8817	8821	8822	1	0	15.00661	3	
8821	8816	8817	8818	4	0	15.00661	2	

4. Molecular dynamics results

4.1. RMSD plots

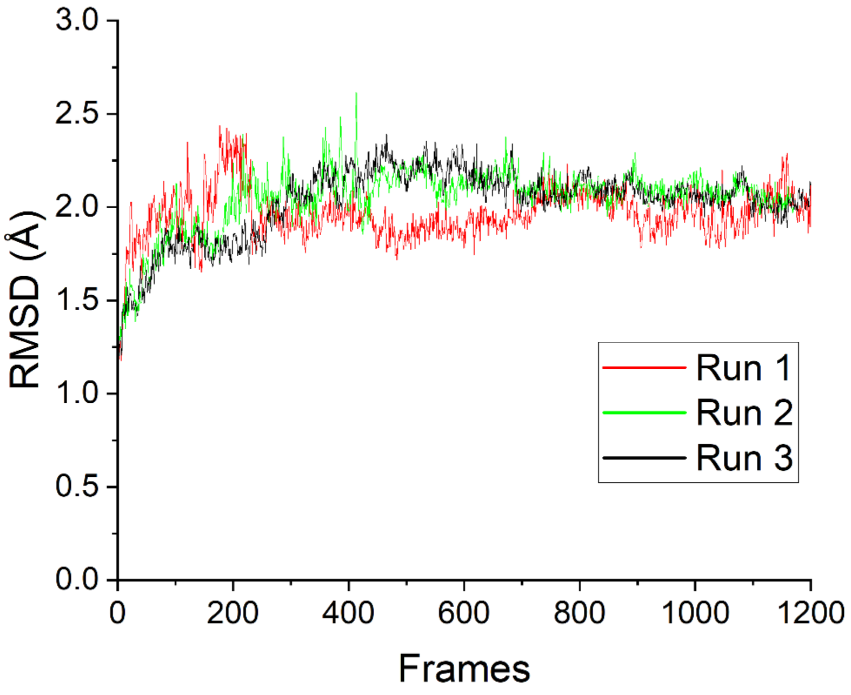

Figure S3. Molecular dynamics simulations showing the RMSD (root-mean-square deviation) of 2-phenylindole in the suggested binding pocket of *Ci*VCPO.

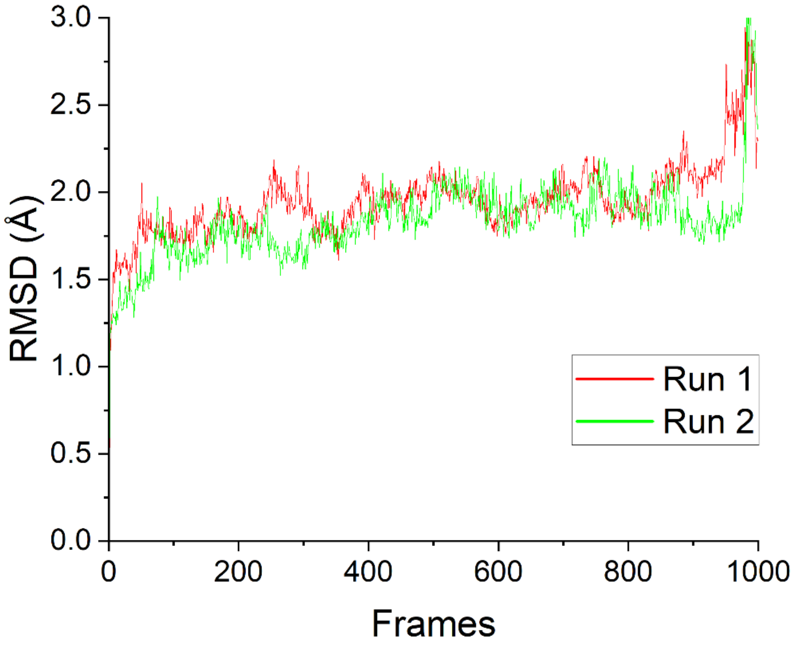

Figure S4. Molecular dynamics simulations showing the RMSD (root-mean-square deviation) of indole in the suggested binding pocket of *Ci*VCPO.

4.2. Distance between the vanadium centre and substrates

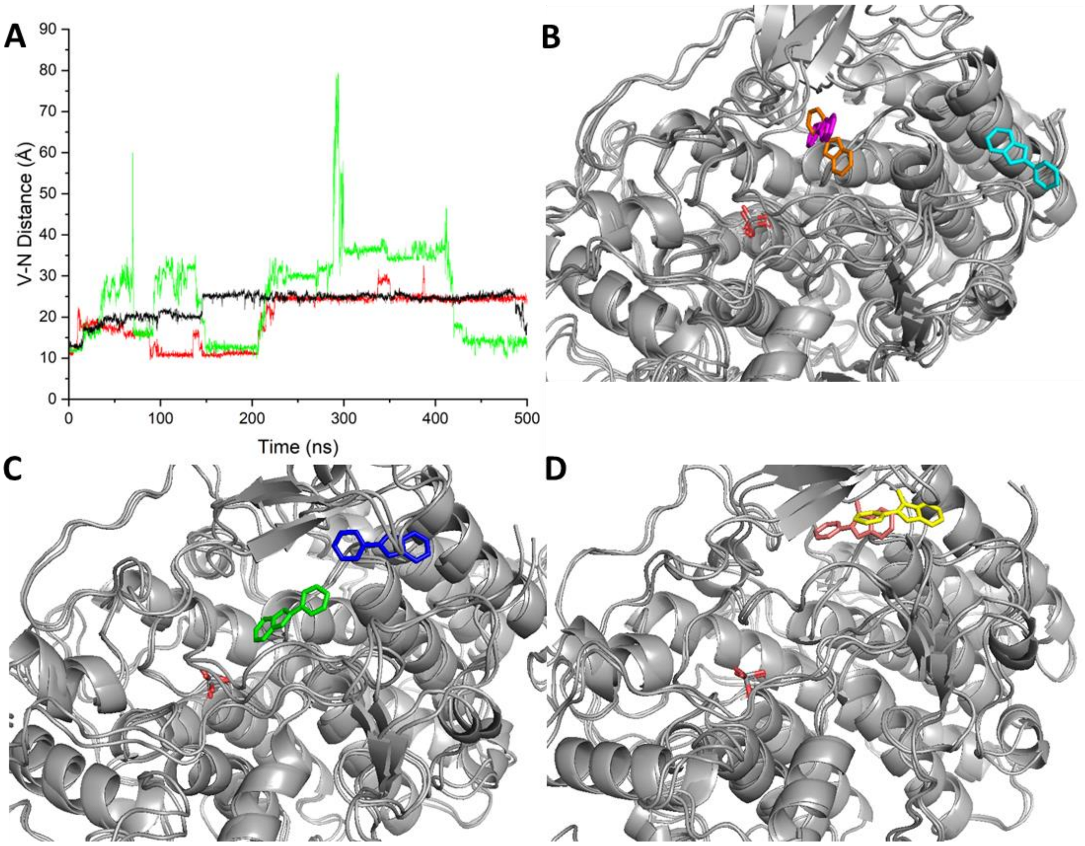

Figure S5. (A) Distance in angstroms between the vanadium atom and the nitrogen on 2-phenylindole (red and green) and methyl-phenylindole (black) during 500 ns molecular dynamics simulations. (B) Snapshots along the MD simulation of the green 500 ns 2-phenylindole run, where the substrate is seen dissociating and rebinding to the substrate binding channel. Orange = 175 ns, cyan = 250 ns and magenta = 500 ns. (C) Snapshots along the MD simulation of the red 500 ns 2-phenylindole run. Green = 125 ns and blue = 250 ns. (D) Snapshots along the MD simulation of the methyl-phenylindole 500 ns run. Pink = 125 ns and yellow = 250 ns.

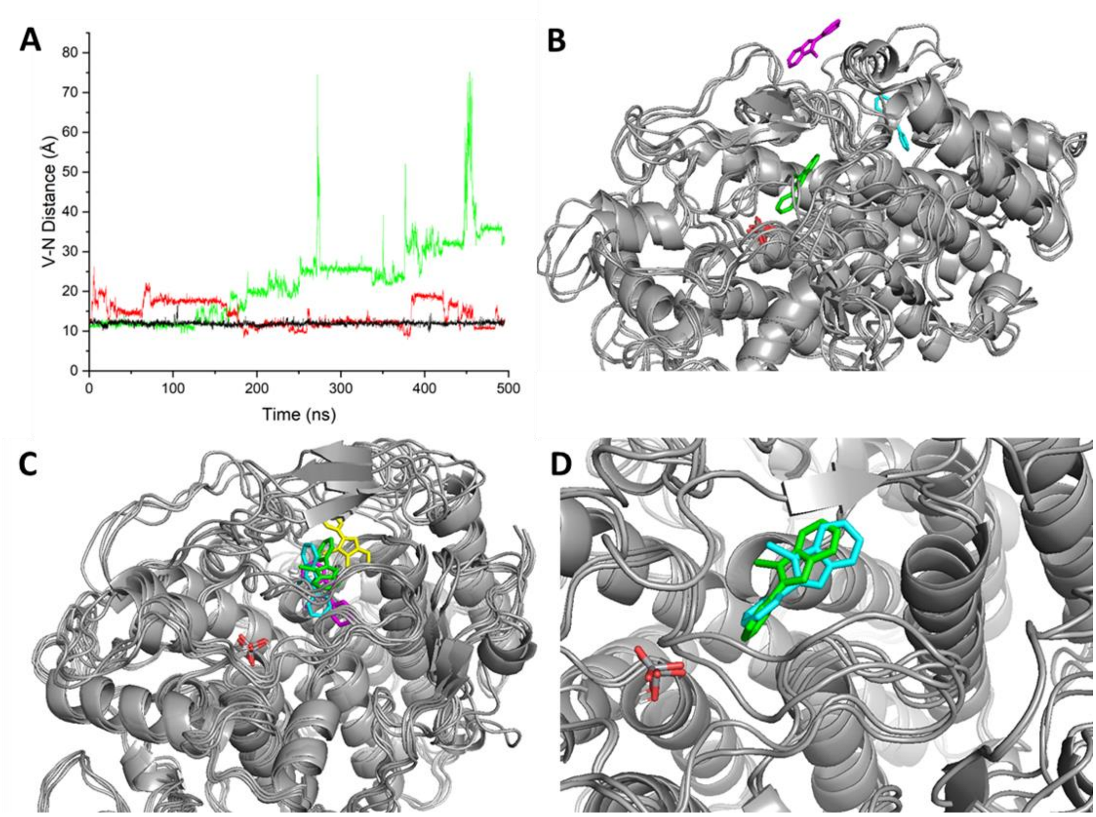

Figure S6. (A) Distance in angstroms between the vanadium atom and the nitrogen on 2-phenyl-3-bromoindole (red and green) and 2-phenyl-3-chloroindole (black) during 500 ns molecular dynamics simulations. (B) Snapshots along the MD simulation of the green 500 ns br-2-phenylindole run, where the substrate is seen dissociating from the substrate binding channel. Green = 50 ns, magenta = 300 ns and cyan = 490 ns. (C) Snapshots along the MD simulation of the red 500 ns br-2-phenylindole run. Green = 50 ns, yellow = 125 ns, magenta = 300 ns and cyan = 490 ns. (D) Snapshots along the MD simulation of the cl-2-phenylindole 500 ns run. Green = 50 ns and cyan = 490 ns.

4.1 Hydrogen-bonding

Table S2. Residues and atoms involved in hydrogen-bonding with 2-phenylindole during the 3 molecular dynamics calculations. Highlighted in green are the residues within 5 Angstroms of 2-phenylindole (2PI) from the docking.

2PI runs	Residue donor	Atom Donor	Residue acceptor	Atom acceptor
Run 1	His38	Oxygen	2PI	H on Nitrogen
	Thr45	Oxygen	2PI	H on Nitrogen
	Pro125	Oxygen	2PI	H on Nitrogen
	Asn128	Oxygen	2PI	H on Nitrogen
	Asn128	Oxygen	2PI	H on Nitrogen
	Gln181	Oxygen	2PI	H on Nitrogen
	Leu203	Oxygen	2PI	H on Nitrogen
	Gln220	Oxygen	2PI	H on Nitrogen
	Asp292	Oxygen	2PI	H on Nitrogen
	Asn124	Oxygen	2PI	H on Nitrogen
	Asn124	Oxygen	2PI	H on Nitrogen
	Pro125	Oxygen	2PI	H on Nitrogen
	Ala127	Oxygen	2PI	H on Nitrogen
Run 2	Ala179	Oxygen	2PI	H on Nitrogen
	Gln220	Oxygen	2PI	H on Nitrogen
	Gln220	Oxygen	2PI	H on Nitrogen
	Gln220	Oxygen	2PI	H on Nitrogen
	2PI	Nitrogen	Asn128	H on Nitrogen
Run 3	Thr39	Oxygen	2PI	H on Nitrogen
	Gln220	Oxygen	2PI	H on Nitrogen

5. Ligand docking

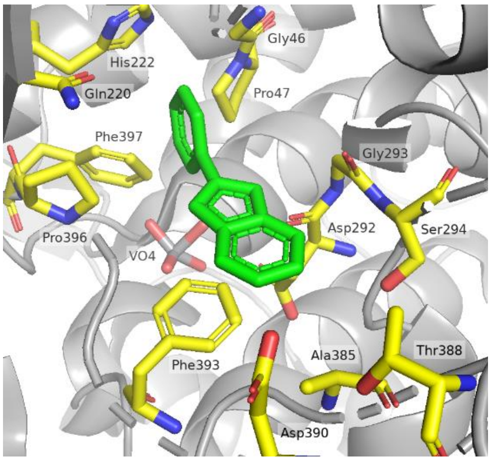

Figure S7. Most favourable binding pose for 2-phenylindole as docked using AutoDock Vina into the 1IDQ pdb structure. Residues selected within 5 Å.

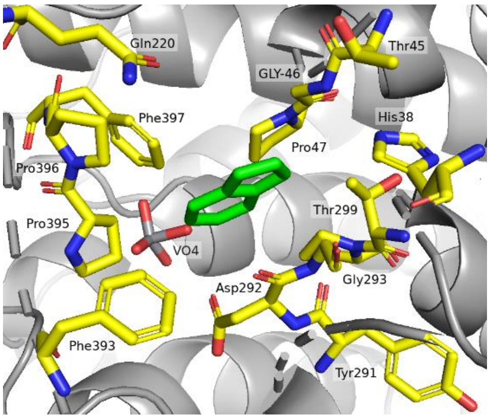

Figure S8. Most favourable binding pose for indole as docked using AutoDock Vina into the 1IDQ pdb structure. Residues selected within 5 Å.

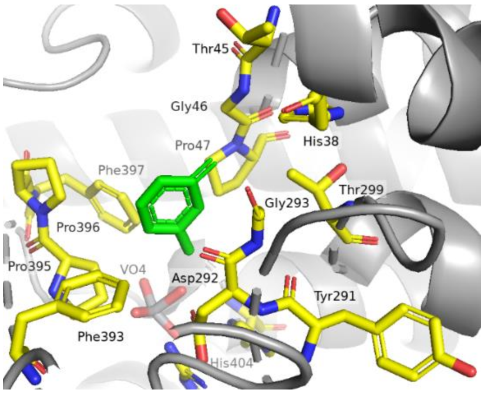

Figure S9. Most favourable binding pose for cytosine as docked using AutoDock Vina into the 1IDQ pdb structure. Residues selected within 5 Å.

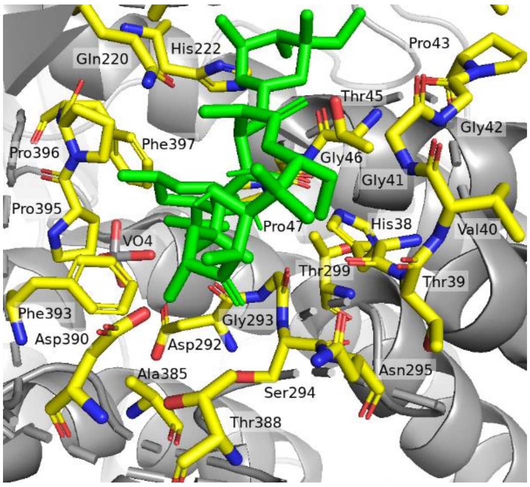

Figure S10. Most favourable binding pose for erythromycin as docked using AutoDock Vina into the 1IDQ pdb structure. Residues selected within 5 Å.

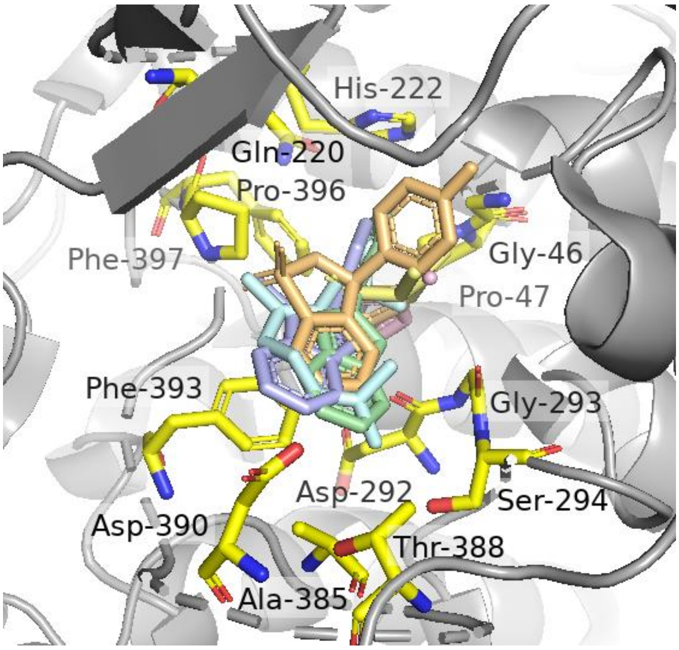

Figure S11. Overlay of all docked substrates into 1IDQ pdb using AutoDock vina. As can be seen all substrates bind in the same area of the protein. Residues highlighted are those within 5 Å of the substrate. 2-phenylindole (green), methylphenylindole (blue), monochlorodimedone (light yellow), styrene (pink), phenol red (orange) and geraniol (light blue).

6. Caver tunnelling analysis

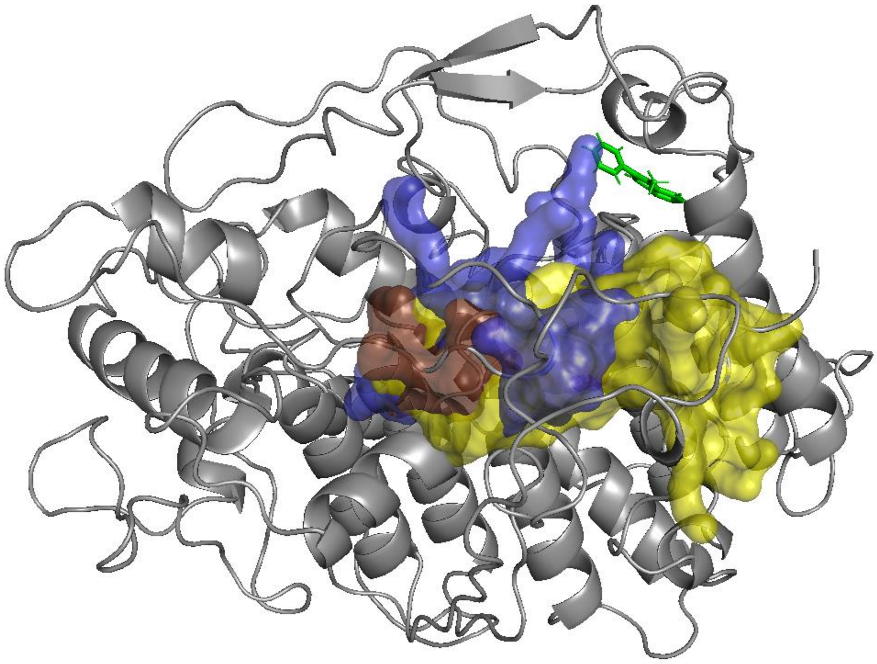

Figure S12. Tunnel analysis of methylphenylindole MD simulation using the Caver Software package showing the channels (red, blue and yellow) linking the vanadate active site to the substrate.

7. Umbrella sampling of HOCl in the substrate channel.

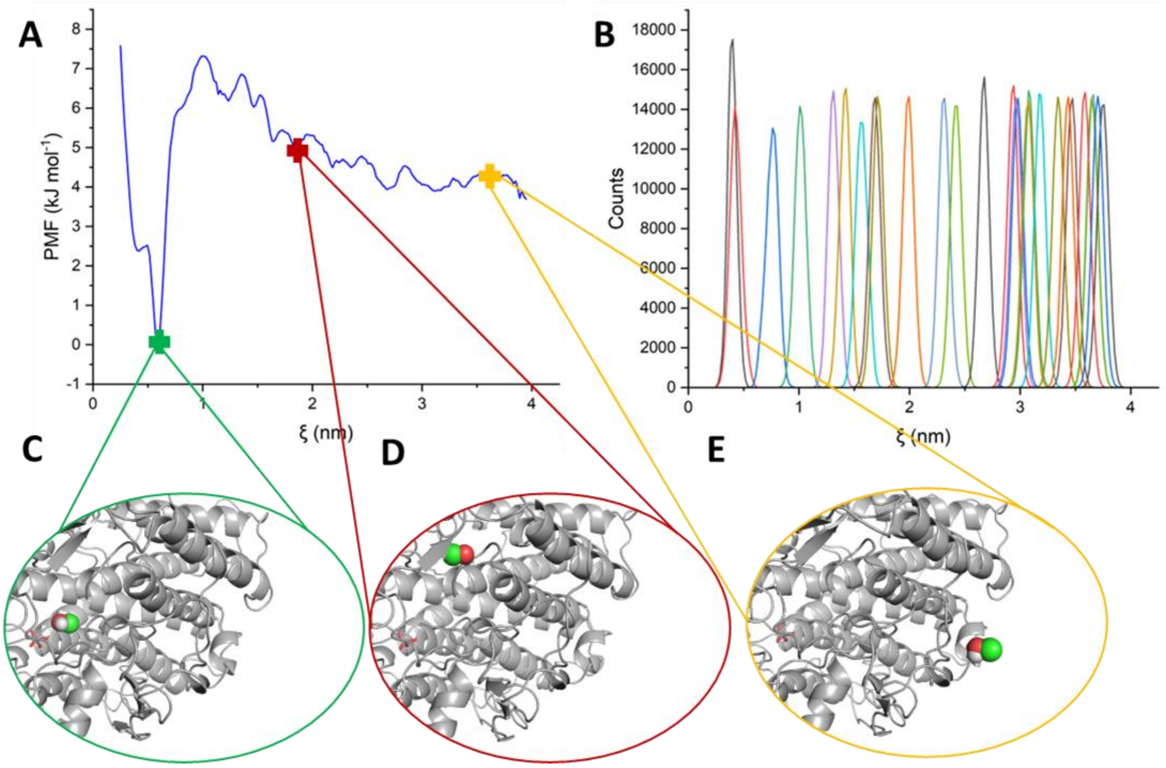

Figure S13. (A) Potential of mean force calculated by umbrella sampling as a function of ξ (distance between the centre of mass of the vanadate and HOCl). (B) Distribution for each point along the umbrella trajectory, each sampled for 10 ns. (C) The dip in the potential of mean force at a ξ of 0.6 nm corresponds to the HOCl (Cl in green) in the vanadate active site. (D) At ξ of 1.8 nm, the HOCl is in the substrate binding pocket. (E) At ξ of 3.6 nm, HOCl is at the end of the substrate channel.

8. Steady-State UV/Vis spectroscopy

Table S3. Michaelis Menten analysis of steady-state kinetics of *Ci*VCPO with 2-phenylindole and methylphenylindole and HOBr and HOCl monitored using fluorescence spectroscopy, with the data shown in Figures S13-S16.

	2-phenylindole			methylphenylindole		
	K_M (μM)	V_{max} (μM s^{-1})	k_{cat} (s^{-1})	K_M (μM)	V_{max} (μM s^{-1})	k_{cat} (s^{-1})
**HOBr**	1.52	0.017363	1.736333	1.58	0.016356	1.6356
**HOCl**	15.48	0.031746	0.634913	46.70	0.033755	0.6751

8.1. Methylphenylindole

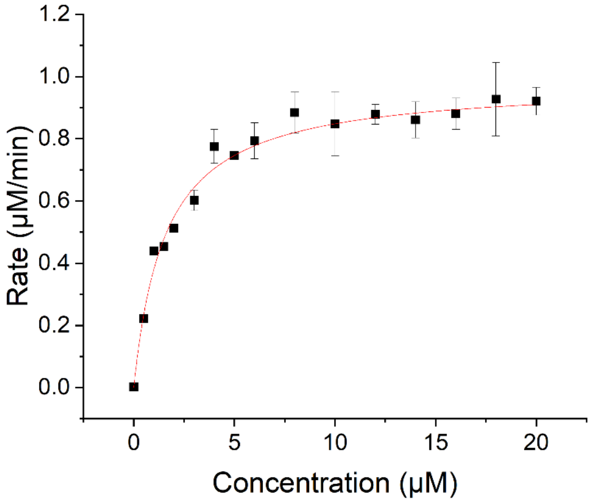

Figure S14. Steady-state kinetics following the reaction of methylphenylindole with *Ci*VCPO (0.01 μM), KBr (20 mM), H_2O_2 (10 mM) and Na_3VO_4 (50 μM). Excitation at 316 nm, following the decrease of the emission peak at 380 nm. The red line is a fit to the Michaelis-Menten equation with fit parameters given in Table S3.

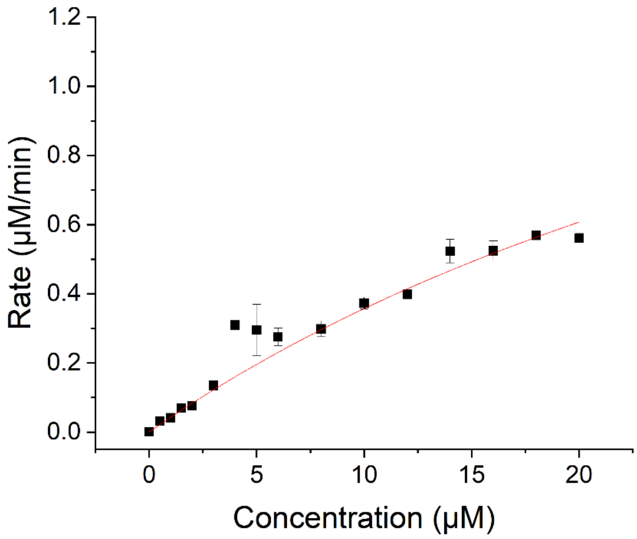

Figure S15. Steady-state kinetics following the reaction of methylphenylindole with *Ci*VCPO (0.05 µM), KCl (20 mM), H_2O_2 (10 mM) and Na_3VO_4 (50 µM). Excitation at 316 nm, following the decrease of the emission peak at 380 nm. The red line is a fit to the Michaelis-Menten equation with fit parameters given in Table S3.

8.2. 2-phenylindole

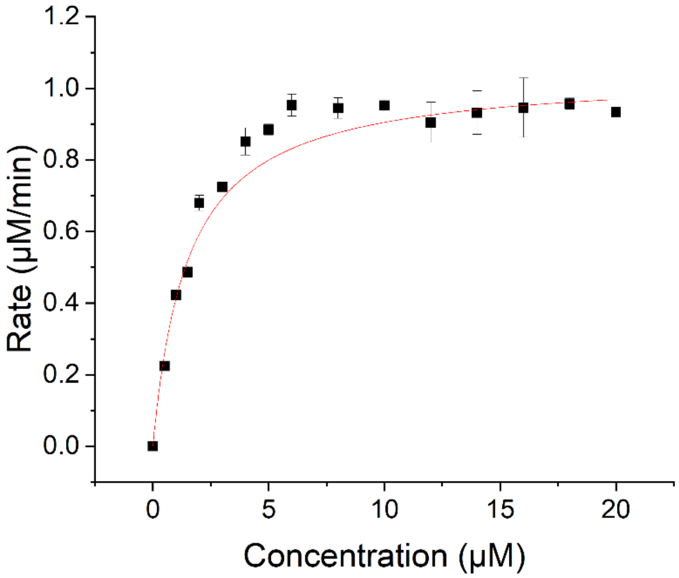

Figure S16. Steady-state kinetics following the reaction of 2-phenylindole with *Ci*VCPO (0.01 µM), KBr (20 mM), H_2O_2 (10 mM) and Na_3VO_4 (50 µM). Excitation at 316 nm, following the decrease of the emission peak at 376 nm. The red line is a fit to the Michaelis-Menten equation with fit parameters given in Table S3.

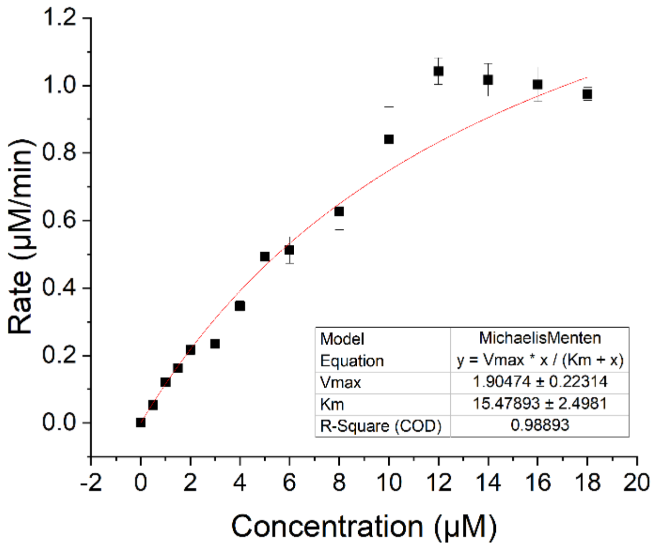

Figure S17. Steady-state kinetics following the reaction of 2-phenylindole with *Ci*VCPO (0.05 µM), KCl (20 mM), H_2O_2 (10 mM) and Na_3VO_4 (50 µM). Excitation at 316 nm, following the decrease of the emission peak at 376 nm. The red line is a fit to the Michaelis-Menten equation with fit parameters given in Table S3.

8.3. Fluorescence spectra

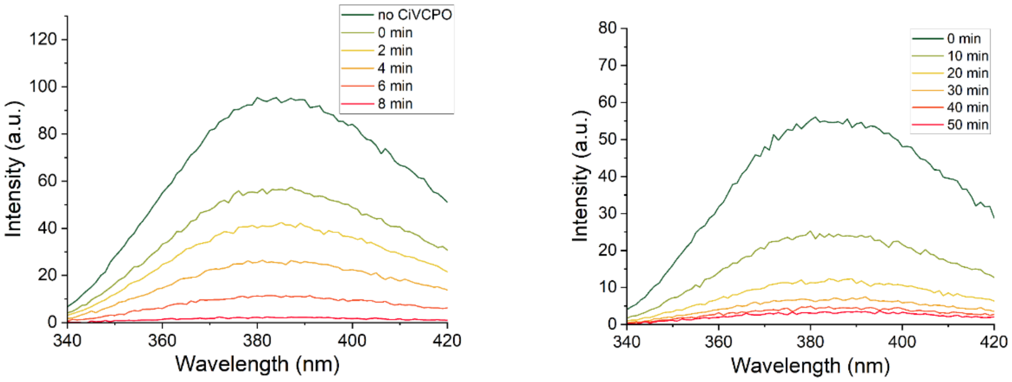

Figure S18. Left: Fluorescence emission spectra after excitation at 316 nm of the reaction of *Ci*VCPO (0.01 µM) and methylphenylindole (15 µM) in the presence of KBr (20 mM), Na_3VO_4 (50 µM) and H_2O_2 (10 mM) in 10% DMSO and MES buffer (50 mM, pH 5.5) over 10 minutes. Right: Fluorescence emission spectra of the reaction of *Ci*VCPO (0.05 µM) and methylphenylindole (15 µM) in the presence of KCl (20 mM), Na_3VO_4 (50 µM) and H_2O_2 (10 mM) in 10% DMSO and MES buffer (50 mM, pH 5.5) over 60 minutes.

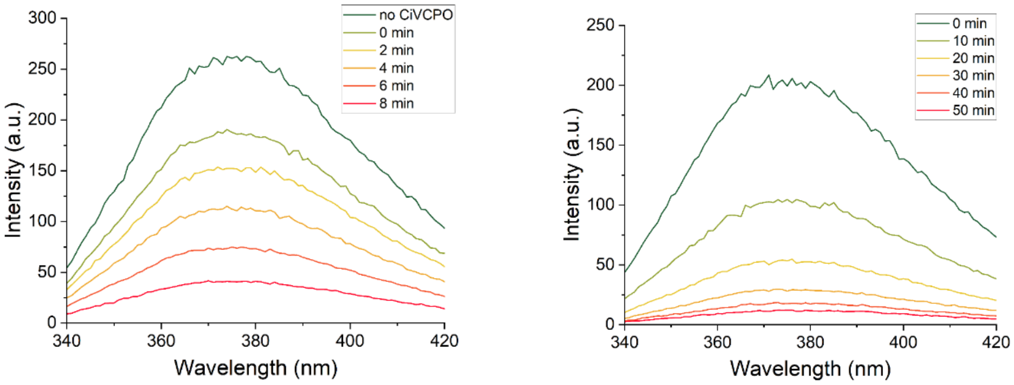

Figure S19. Left: Fluorescence emission spectra after excitation at 316 nm of the reaction of *Ci*VCPO (0.01 µM) and 2-phenylindole (15 µM) in the presence of KBr (20 mM), Na_3VO_4 (50 µM) and H_2O_2 (10 mM) in 10% DMSO and MES buffer (50 mM, pH 5.5) over 10 minutes. Right: Fluorescence emission spectra of the reaction of *Ci*VCPO (0.05 µM) and 2-phenylindole (15 µM) in the presence of KCl (20 mM), Na_3VO_4 (50 µM) and H_2O_2 (10 mM) in 10% DMSO and MES buffer (50 mM, pH 5.5) over 60 minutes.

9. Stopped-Flow UV/Vis spectroscopy

Table S4. Stopped-flow fluorescence measurements monitoring the observed reaction times, $R_{95\%}$ (s) of halogenation of methylphenylindole when mixing components of syringe 1 with syringe 2. KX = KBr or KCl, SUB = methylphenylindole.

	Reagent 1	Reagent 2	Methylphenylindole	
			KBr	KCl
Condition 1	*Ci*VCPO	SUB, KX, H_2O_2	0.203 ± 0.007	0.396 ± 0.006
Condition 2	*Ci*VCPO, SUB	KX, H_2O_2	0.162 ± 0.001	0.229 ± 0.003
Condition 3	*Ci*VCPO, H_2O_2	SUB, KX	0.074 ± 0.002	0.190 ± 0.007

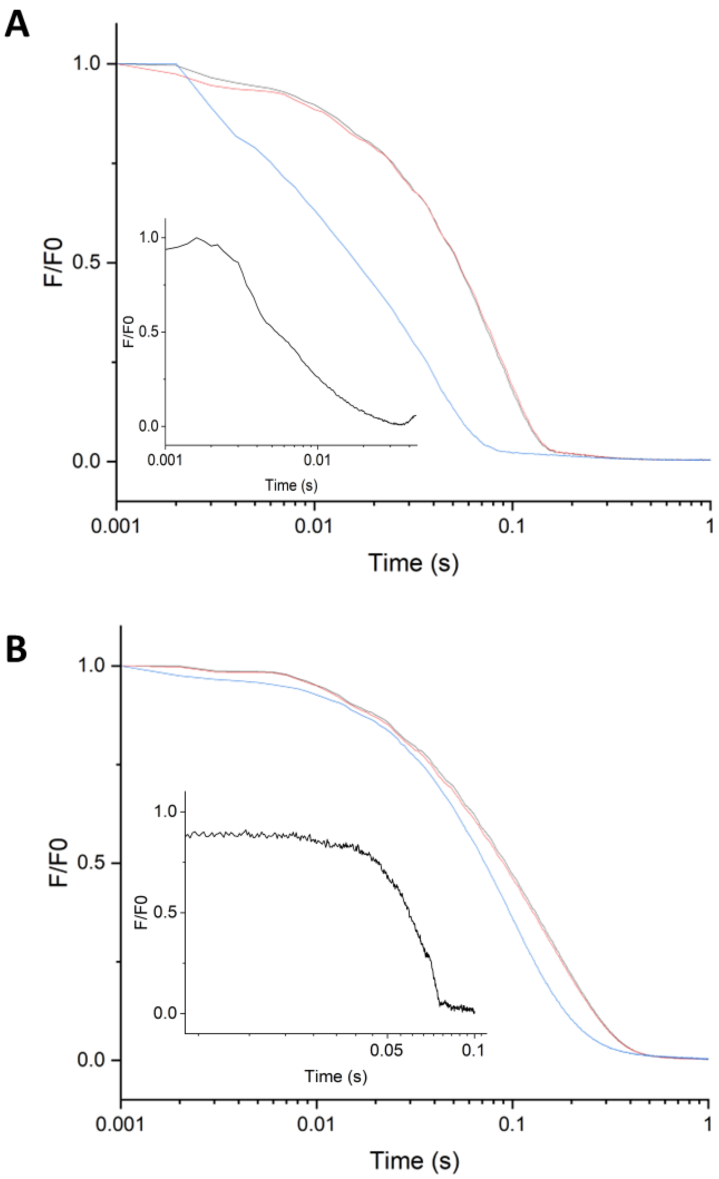

Figure S20. Stopped-flow kinetics. Condition 1 from Table S4 (Black): *Ci*VCPO vs SUB (2-phenylindole, H_2O_2, KX. Condition 2 (Red): *Ci*VCPO, SUB (2-phenylindole) vs H_2O_2, KX. Condition 3 (Blue): *Ci*VCPO, H_2O_2 vs SUB (2-phenylindole), KX. Condition 4 (Inset, Black): *Ci*VCPO, H_2O_2, KX vs SUB (2-phenylindole). A: KX = KBr. B: KX = KCl.

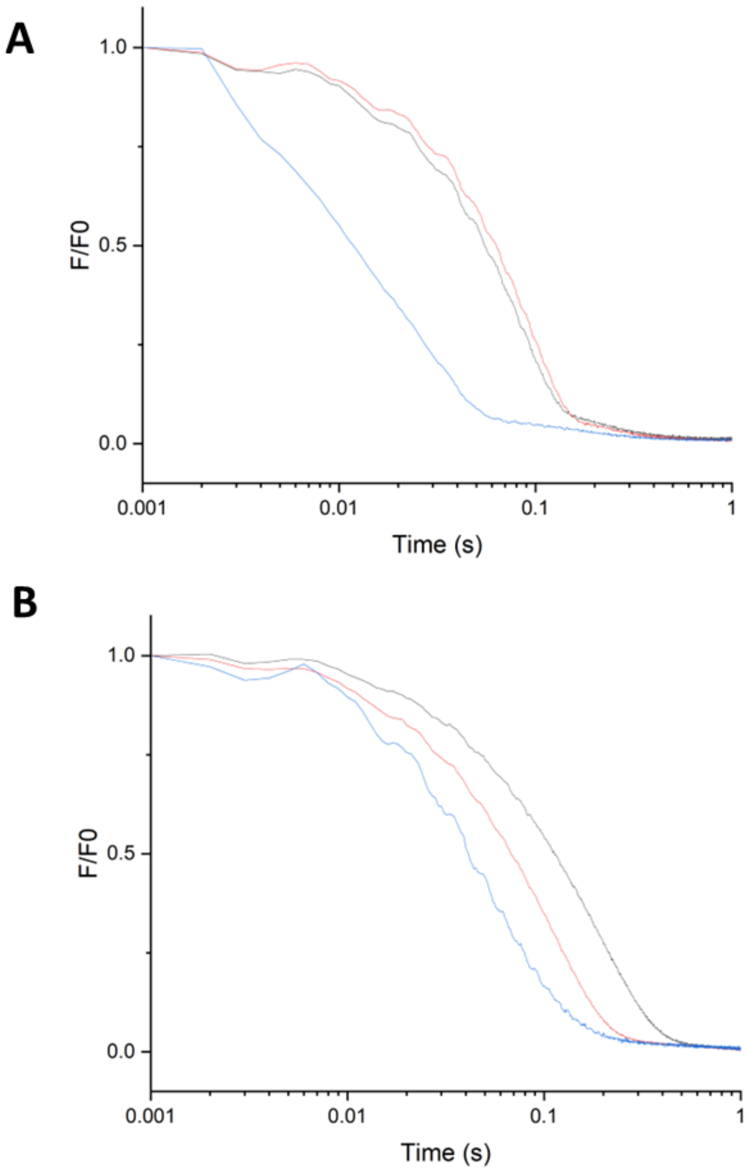

Figure S21. Stopped-flow kinetics. Condition 1 (Black): *Ci*VCPO vs SUB (methylphenylindole, H_2O_2, KX. Condition 2 (Red): *Ci*VCPO, SUB (methylphenylindole) vs H_2O_2, KX. Condition 3 (Blue): *Ci*VCPO, H_2O_2 vs SUB (methylphenylindole), KX. A: KX = KBr. B: KX = KCl.

10. Product analysis

10.1. GCMS

10.1.1. Methylphenylindole

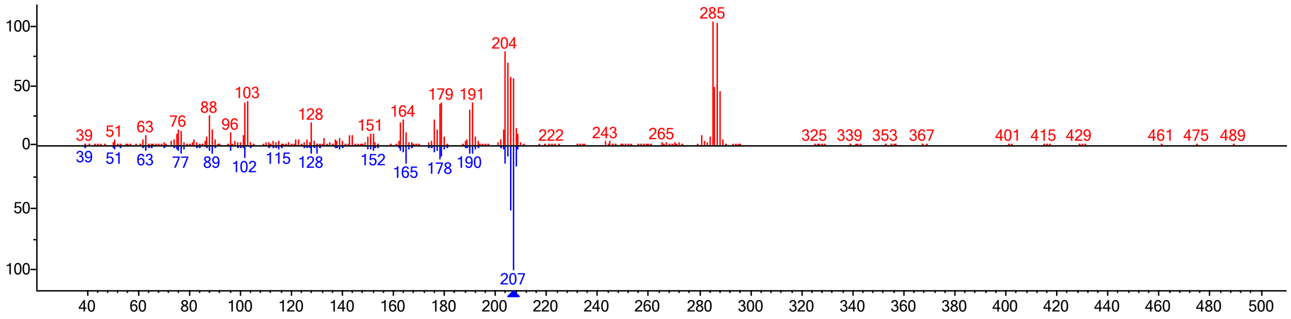

Figure S22. Product analysis for the reaction of *Ci*VCPO with H_2O_2, methylphenylindole and KBr. The main product is Br-methylphenylindole as identified with GCMS. Bromine isotopic peak at 285 g/mol.

10.1.2. 2-phenylindole

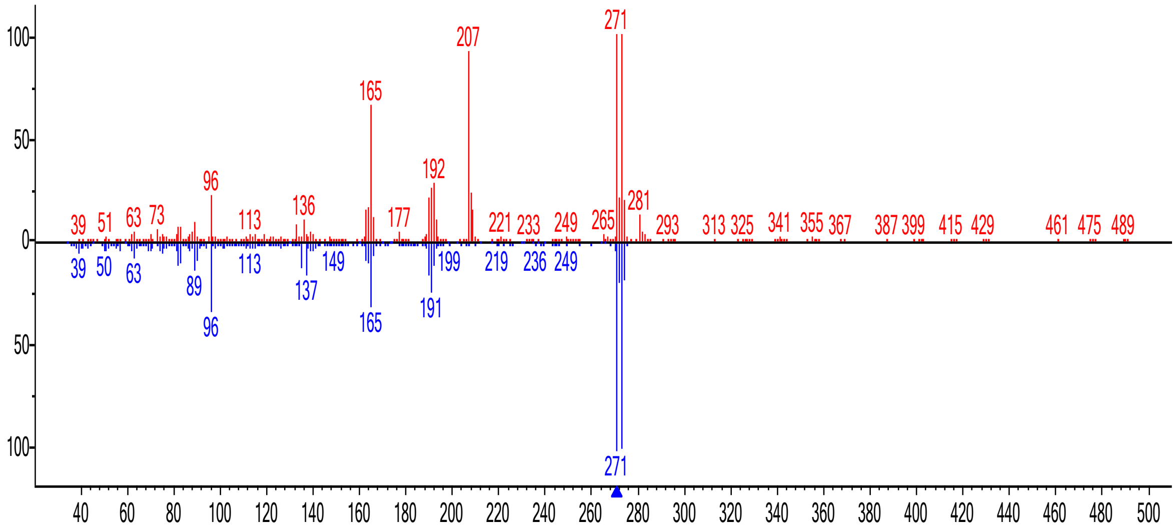

Figure S23. Product analysis for the reaction of *Ci*VCPO with H_2O_2, 2-phenylindole and KBr. The main product is Br-2-phenylindole as identified with GCMS. Bromine isotopic peak at 271 g/mol.

10.2. NMR

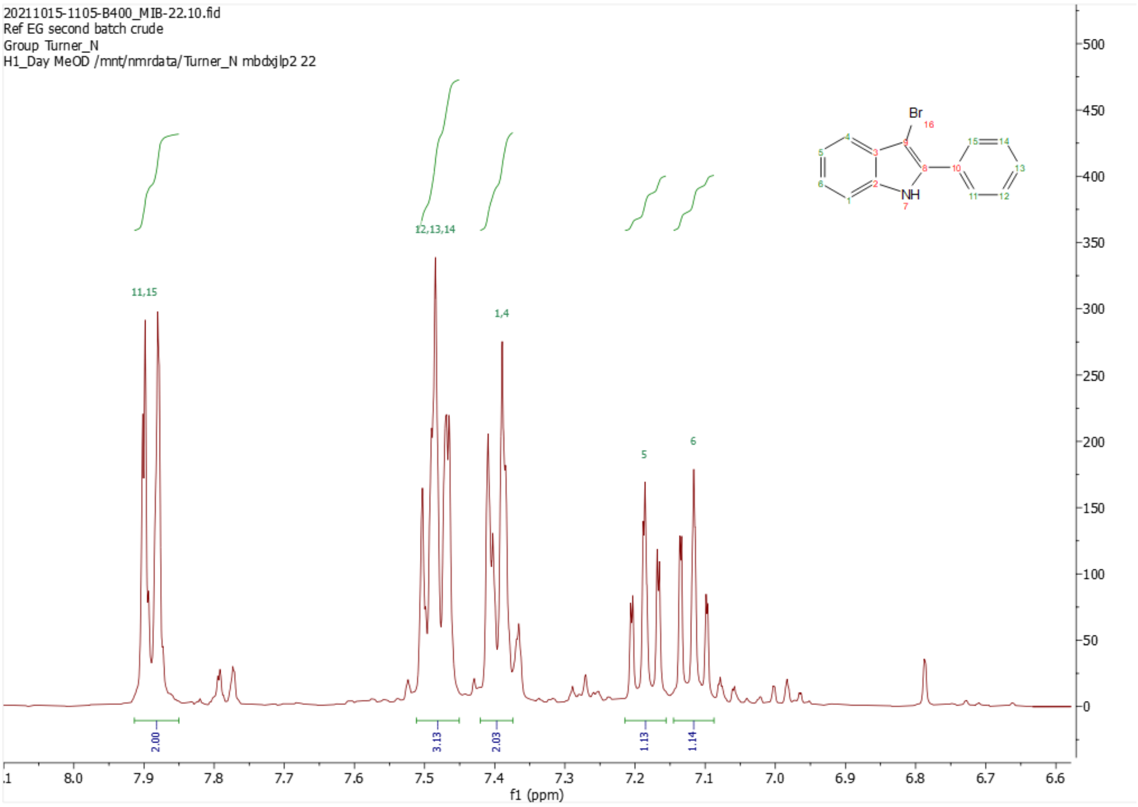

Figure S24. NMR characterization of Br-2-phenylindole.

11. pH-dependence of enzyme activity

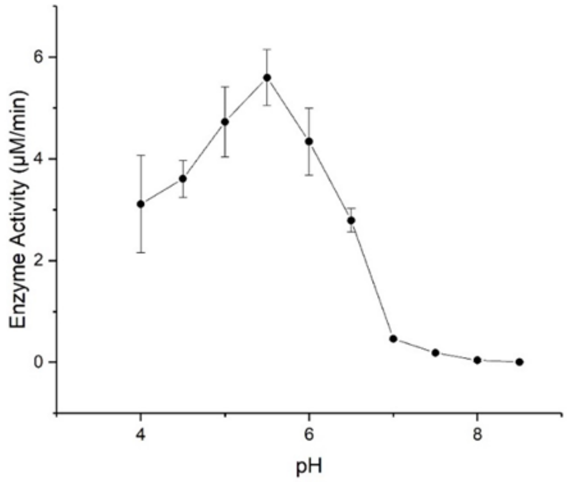

Figure S25. pH profile of *Ci*VCPO activity measured using monochlorodimedone as substrate in 50 mM buffers (NaOAc pH 4.0, 4.5, 5.0 and 5.5; MES pH 6.0 and 6.5; Tris pH 7.0, 7.5, 8.0 and 8.5); 50 μM MCD; 10 mM H_2O_2; 200 mM KBr; 100 μM Na_3VO_4; 0.12 μM *Ci*VCPO. The absorbance decrease at 290 nm was followed over a minute. Enzyme activity was converted to μM/min from Abs/min using the Beer-Lambert law (A = εCl).

12. Residue types on *Ci*VCPO surface

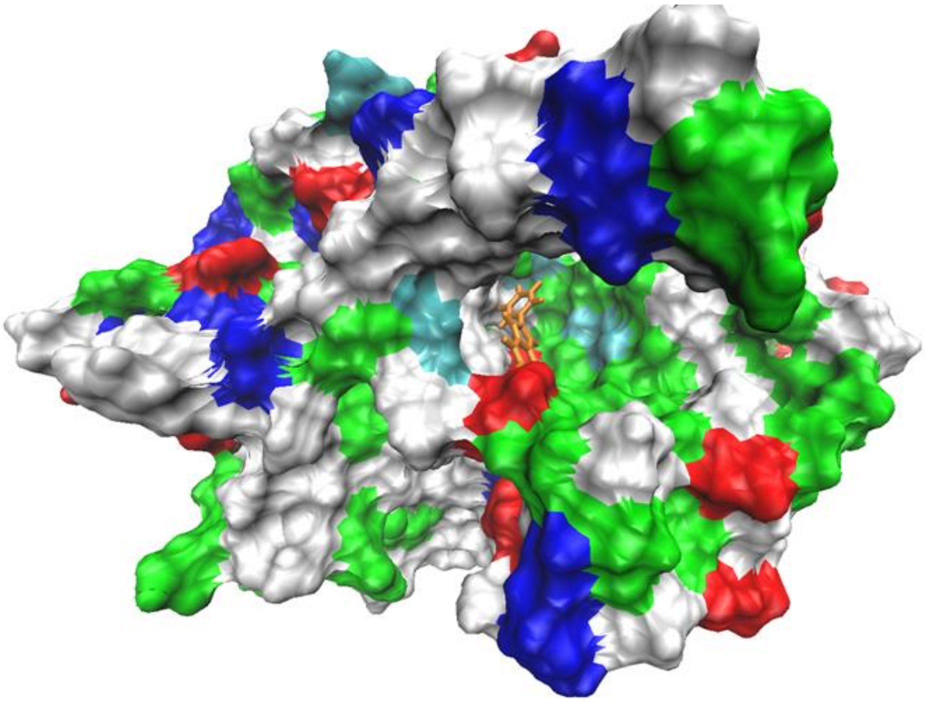

Figure S26. Snapshot of protein surface showing 2-phenylindole (orange) bound at the channel entrance. Residues are coloured by type: polar residues (green), acidic residues (red), basic residues (blue) and non-polar residues (white).

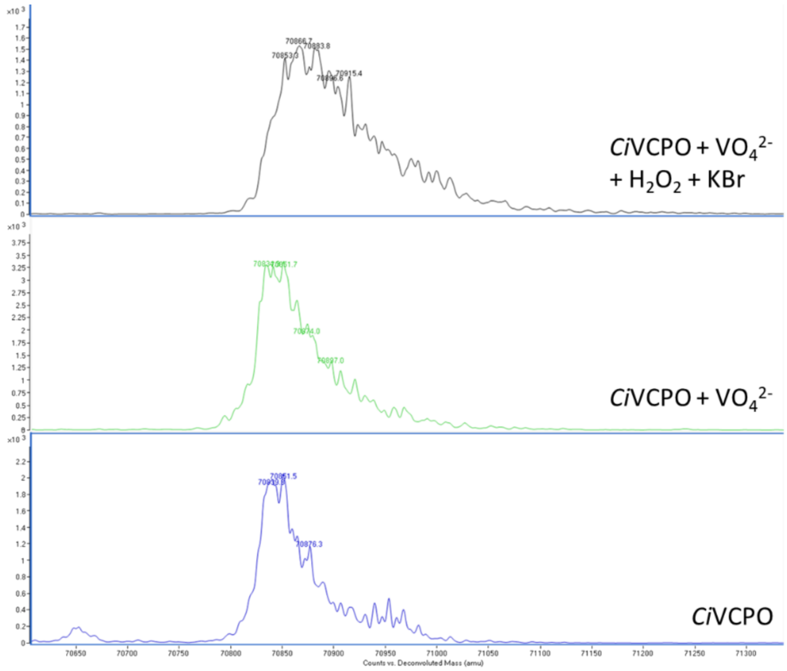

Figure S27. Mass spectroscopy analysis of *Ci*VCPO samples before and after reaction with H_2O_2 and KBr.

13. Optimized geometries for the transition states for HOBr

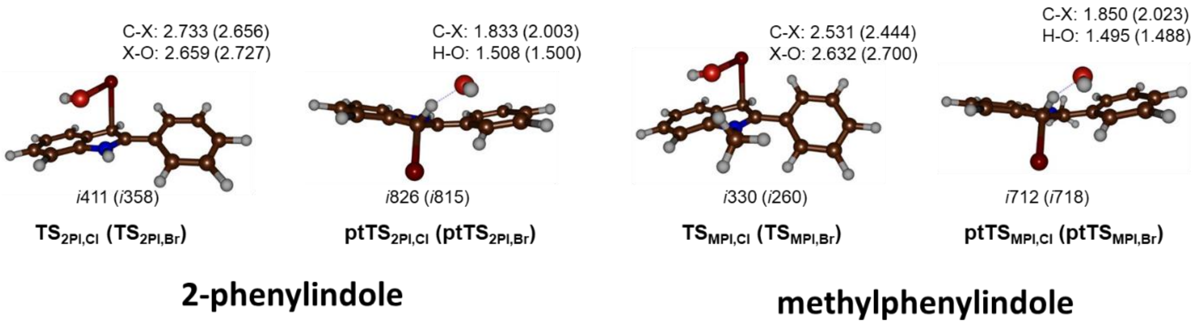

Figure S28. Optimized geometries for the transition states for HOBr with 2-phenylindole and methylphenylindole calculated with implicit solvent model. The structures show bond lengths in Å and the imaginary frequency of the transition state in cm^{-1}.

14. Optimized QM/MM geometry for the HOCl / 2-phenylindole system

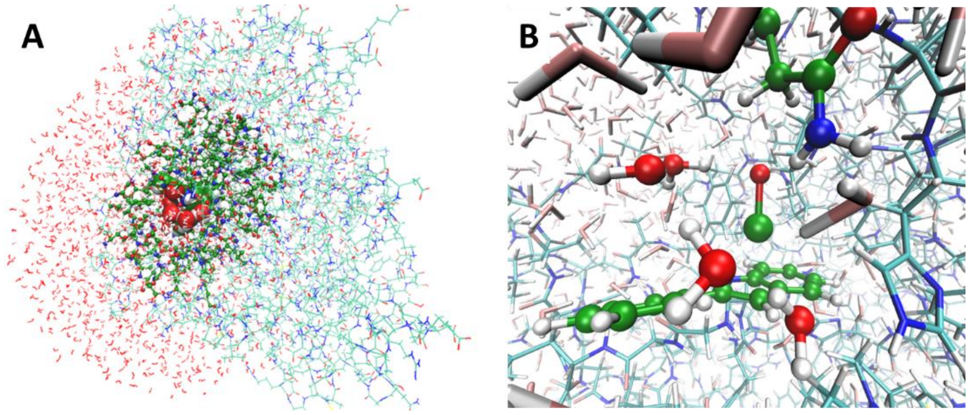

Figure S29. Optimized QM/MM geometry for the HOCl / 2-phenylindole system: (A) Entire system with fixed MM atoms shown as lines, free MM atoms as sticks and QM region as a van-der Waals surface; (B) close-up of active site showing MM region as sticks and QM region using the ball-and-stick representation. Key distances are C-Cl: 2.47 Å, O-Cl: 1.86 Å.

15. Density Functional Theory (DFT) data

Gas-phase calculations:

Table S5. Absolute energies, zero-point energies and free energies (in a.u.) of optimized geometries for the reaction of 2-phenylindole with HOCl as obtained in Gaussian-09. All calculations were done in the gas phase.

	E [au]	ZPE [au]	G [au]	E [au]
	B3LYP/BS1	B3LYP/BS1	B3LYP/BS1	B3LYP/BS2
2-phenylindole	-594.8788796	0.211073	-594.7052	-595.0085638
HOCl	-535.9435702	0.013047	-535.9535	-535.9998548
**Re**$_{2PI, Cl}$	-1130.830141	0.22552	-1130.652621	-1131.015673
**TS**$_{2PI, Cl}$	-1130.740315	0.223346	-1130.560801	-1130.93319
**Int**$_{2PI, Cl}$	-1130.827122	0.223873	-1130.644583	-1131.020925
**ptTS**$_{2PI, Cl}$	-1130.785604	0.22214	-1130.628089	-1130.978676
**Pr**$_{2PI, Cl}$	-1130.884893	0.224544	-1130.70709	-1131.072923

Table S6. Relative energies, zero-point energies and free energies (in kcal mol^{-1}) of optimized geometries for the reaction of 2-phenylindole with HOCl as obtained in Gaussian-09.

	ΔE	ΔE+ZPE	ΔG	ΔE	ΔE+ZPE	ΔG
	BS1	BS1	BS1	BS2	BS2	BS2
**Re**$_{2PI, Cl}$	0.00	0.00	0.00	0.00	0.00	0.00
**TS**$_{2PI, Cl}$	56.37	55.00	57.62	51.76	50.39	53.01
**Int**$_{2PI, Cl}$	1.89	0.86	5.04	-3.30	-4.33	-0.15
**ptTS**$_{2PI, Cl}$	27.95	25.83	15.39	23.22	21.09	10.66
**Pr**$_{2PI, Cl}$	-34.36	-34.97	-34.18	-35.92	-36.54	-35.75

Table S7. Absolute energies, zero-point energies and free energies (in a.u.) of optimized geometries for the reaction of 2-phenylindole with HOBr as obtained in Gaussian-09. All calculations were done in the gas phase.

	E [au]	ZPE [au]	G [au]	E [au]
	B3LYP/BS1	B3LYP/BS1	B3LYP/BS1	B3LYP/BS2
2-phenylindole	-594.8788796	0.211073	-594.7052	-595.0085638
HOBr	-2647.464876	0.012688	-2647.476	-2649.936227
**Re**$_{2PI, Br}$	-3242.354123	0.225005	-3242.175311	-3244.948512
**TS**$_{2PI, Br}$	-3242.268288	0.223502	-3242.08885	-3244.870221
**Int**$_{2PI, Br}$	-3242.340922	0.223264	-3242.159945	-3244.94312
**ptTS**$_{2PI, Br}$	-3242.300627	0.221488	-3242.120775	-3244.90085
**Pr**$_{2PI, Br}$	-3242.400132	0.225019	-3242.221485	-3244.995397

Table S8. Relative energies, zero-point energies and free energies (in kcal mol^{-1}) of optimized geometries for the reaction of 2-phenylindole with HOBr as obtained in Gaussian-09.

	ΔE	ΔE+ZPE	ΔG	ΔE	ΔE+ZPE	ΔG
	BS1	BS1	BS1	BS2	BS2	BS2
**Re**$_{2PI, Br}$	0.00	0.00	0.00	0.00	0.00	0.00
**TS**$_{2PI, Br}$	53.86	52.92	54.25	49.13	48.18	49.52
**Int**$_{2PI, Br}$	8.28	7.19	9.64	3.38	2.29	4.74
**ptTS**$_{2PI, Br}$	33.57	31.36	34.22	29.91	27.70	30.56
**Pr**$_{2PI, Br}$	-28.87	-28.86	-28.97	-29.42	-29.41	-29.52

Table S9. Absolute energies, zero-point energies and free energies (in a.u.) of optimized geometries for the reaction of methylphenylindole with HOCl as obtained in Gaussian-09. All calculations were done in the gas phase.

	E [au]	ZPE [au]	G [au]	E [au]
	B3LYP/BS1	B3LYP/BS1	B3LYP/BS1	B3LYP/BS2
Methylphenylindole	-634.186844	0.239331	-633.986939	-634.3226203
HOCl	-535.9435702	0.013047	-535.9535	-535.9998548
**Re**$_{MPI, Cl}$	-1170.139284	0.254004	-1169.93401	-1170.33005
**TS**$_{MPI, Cl}$	-1170.055677	0.252141	-1169.847398	-1170.254739
**Int**$_{MPI, Cl}$	-1170.099195	0.251565	-1169.893917	-1170.294121
**ptTS**$_{MPI, Cl}$	-1170.092509	0.250892	-1169.883637	-1170.29231
**Pr**$_{MPI, Cl}$	-1170.196977	0.253372	-1169.99085	-1170.393391

Table S10. Relative energies, zero-point energies and free energies (in kcal mol^{-1}) of optimized geometries for the reaction of methylphenylindole with HOCl as obtained in Gaussian-09.

	ΔE	ΔE+ZPE	ΔG	ΔE	ΔE+ZPE	ΔG
	BS1	BS1	BS1	BS2	BS2	BS2
**Re**$_{MPI, Cl}$	0.00	0.00	0.00	0.00	0.00	0.00
**TS**$_{MPI, Cl}$	52.46	51.29	54.35	47.26	46.09	49.14
**Int**$_{MPI, Cl}$	25.16	23.63	25.16	22.55	21.01	22.55
**ptTS**$_{MPI, Cl}$	29.35	27.40	31.61	23.68	21.73	25.94
**Pr**$_{MPI, Cl}$	-36.20	-36.60	-35.67	-39.75	-40.14	-39.21

Table S11. Absolute energies, zero-point energies and free energies (in a.u.) of optimized geometries for the reaction of methylphenylindole with HOBr as obtained in Gaussian-09. All calculations were done in the gas phase.

	E [au]	ZPE [au]	G [au]	E [au]
	B3LYP/BS1	B3LYP/BS1	B3LYP/BS1	B3LYP/BS2
Methylphenylindole	-634.186844	0.239331	-633.986939	-634.3226203
HOBr	-2647.464876	0.012688	-2647.476	-2649.936227
**Re**$_{MPI, Br}$	-3281.661656	0.253849	-3281.456761	-3284.26444
**TS**$_{MPI, Br}$	-3281.585129	0.251999	-3281.377953	-3284.191629
**Int**$_{MPI, Br}$	-3281.620028	0.251454	-3281.416232	-3284.222291
**ptTS**$_{MPI, Br}$	-3281.608179	0.250121	-3281.401157	-3284.214739
**Pr**$_{MPI, Br}$	-3281.711004	0.253311	-3281.505228	-3284.312846

Table S12. Relative energies, zero-point energies and free energies (in kcal mol^{-1}) of optimized geometries for the reaction of methylphenylindole with HOBr as obtained in Gaussian-09.

	ΔE	ΔE+ZPE	ΔG	ΔE	ΔE+ZPE	ΔG
	BS1	BS1	BS1	BS2	BS2	BS2
**Re**$_{MPI, Br}$	0.00	0.00	0.00	0.00	0.00	0.00
**TS**$_{MPI, Br}$	48.02	46.86	49.45	45.69	44.53	47.12
**Int**$_{MPI, Br}$	26.12	24.62	25.43	26.45	24.95	25.76
**ptTS**$_{MPI, Br}$	33.56	31.22	34.89	31.19	28.85	32.52
**Pr**$_{MPI, Br}$	-30.97	-31.30	-30.41	-30.37	-30.71	-29.82

Implicit solvent model calculations:

Table S13. Absolute energies, zero-point energies and free energies (in a.u.) of optimized geometries for the reaction of 2-phenylindole with HOCl as obtained in Gaussian-09. All calculations were done with a continuum polarized conductor model (CPCM) included with the solvent Methanol that has a dielectric constant of 32.7.

	E [au]	ZPE [au]	G [au]	E [au]
	B3LYP/BS1	B3LYP/BS1	B3LYP/BS1	B3LYP/BS2
2-phenylindole	-594.8868549	0.211086	-594.713248	-595.0177616
HOCl	-535.948647	0.013035	-535.958602	-536.0058532
**Re**$_{2PI, Cl}$	-1130.840795	0.225339	-1130.663372	-1131.028003
**TS**$_{2PI, Cl}$	-1130.766869	0.222991	-1130.588298	-1130.965813
**Int**$_{2PI, Cl}$	-1130.845934	0.224566	-1130.662663	-1131.044588
**ptTS**$_{2PI, Cl}$	-1130.80518	0.223816	-1130.622535	-1131.006825
**Pr**$_{2PI, Cl}$	-1130.897976	0.224307	-1130.720519	-1131.0923

Table S14. Relative energies, zero-point energies and free energies (in kcal mol^{-1}) of optimized geometries for the reaction of 2-phenylindole with HOCl as obtained in Gaussian-09.

	ΔE	ΔE+ZPE	ΔG	ΔE	ΔE+ZPE	ΔG
	BS1	BS1	BS1	BS2	BS2	BS2
**Re**$_{2PI, Cl}$	0.00	0.00	0.00	0.00	0.00	0.00
**TS**$_{2PI, Cl}$	46.39	44.92	47.11	39.02	37.55	39.74
**Int**$_{2PI, Cl}$	-3.22	-3.71	0.44	-10.41	-10.89	-6.74
**ptTS**$_{2PI, Cl}$	22.35	21.39	25.63	13.29	12.33	16.57
**Pr**$_{2PI, Cl}$	-35.88	-36.53	-35.86	-40.35	-40.99	-40.32

Table S15. Absolute energies, zero-point energies and free energies (in a.u.) of optimized geometries for the reaction of 2-phenylindole with HOBr as obtained in Gaussian-09. All calculations were done with a continuum polarized conductor model (CPCM) included with the solvent Methanol that has a dielectric constant of 32.7.

	E [au]	ZPE [au]	G [au]	E [au]
	B3LYP/BS1	B3LYP/BS1	B3LYP/BS1	B3LYP/BS2
2-phenylindole	-594.8868549	0.211086	-594.713248	-595.0177616
HOBr	-2647.469917	0.012662	-2647.481463	-2649.942373
**Re**$_{2PI, Br}$	-3242.364552	0.224899	-3242.185934	-3244.961348
**TS**$_{2PI, Br}$	-3242.290198	0.223037	-3242.113039	-3244.871865
**Int**$_{2PI, Br}$	-3242.359845	0.223955	-3242.178131	-3244.966766
**ptTS**$_{2PI, Br}$	-3242.320318	0.223468	-3242.138826	-3244.929143
**Pr**$_{2PI, Br}$	-3242.413078	0.224976	-3242.23419	-3245.010901

Table S16. Relative energies, zero-point energies and free energies (in kcal mol^{-1}) of optimized geometries for the reaction of 2-phenylindole with HOBr as obtained in Gaussian-09.

	ΔE	ΔE+ZPE	ΔG	ΔE	ΔE+ZPE	ΔG
	BS1	BS1	BS1	BS2	BS2	BS2
**Re**$_{2PI, Br}$	0.00	0.00	0.00	0.00	0.00	0.00
**TS**$_{2PI, Br}$	46.66	45.49	45.74	56.15	54.98	55.23
**Int**$_{2PI, Br}$	2.95	2.36	4.90	-3.40	-3.99	-1.46
**ptTS**$_{2PI, Br}$	27.76	26.86	29.56	20.21	19.31	22.01
**Pr**$_{2PI, Br}$	-30.45	-30.40	-30.28	-31.09	-31.05	-30.93

Table S17. Absolute energies, zero-point energies and free energies (in a.u.) of optimized geometries for the reaction of methylphenylindole with HOCl as obtained in Gaussian-09 All calculations were done with a continuum polarized conductor model (CPCM) included with the solvent methanol that has a dielectric constant of 32.7.

	E [au]	ZPE [au]	G [au]	E [au]
	B3LYP/BS1	B3LYP/BS1	B3LYP/BS1	B3LYP/BS2
Methylphenylindole	-634.1932731	0.239308	-633.993332	-634.3301322
HOCl	-535.948647	0.013035	-535.958602	-536.0058532
**Re**$_{MPI, Cl}$	-1170.14712	0.253752	-1169.942057	-1170.339399
**TS**$_{MPI, Cl}$	-1170.055677	0.25214	-1169.847401	-1170.254741
**Int**$_{MPI, Cl}$	-1170.11874	0.250868	-1169.91522	-1170.316505
**ptTS**$_{MPI, Cl}$	-1170.111875	0.252641	-1169.901662	-1170.320216
**Pr**$_{MPI, Cl}$	-1170.207025	0.252988	-1170.001735	-1170.393391

Table S18. Relative energies, zero-point energies and free energies (in kcal mol^{-1}) of optimized geometries for the reaction of methylphenylindole with HOCl as obtained in Gaussian-09.

	ΔE	ΔE+ZPE	ΔG	ΔE	ΔE+ZPE	ΔG
	BS1	BS1	BS1	BS2	BS2	BS2
**Re**$_{MPI, Cl}$	0.00	0.00	0.00	0.00	0.00	0.00
**TS**$_{MPI, Cl}$	57.38	56.37	59.40	53.12	52.11	55.14
**Int**$_{MPI, Cl}$	17.81	16.00	16.84	14.37	12.56	13.40
**ptTS**$_{MPI, Cl}$	22.12	21.42	25.35	12.04	11.34	15.27
**Pr**$_{MPI, Cl}$	-37.59	-38.07	-37.45	-33.88	-34.36	-33.74

Table S19. Absolute energies, zero-point energies and free energies (in a.u.) of optimized geometries for the reaction of methylphenylindole with HOBr as obtained in Gaussian-09. All calculations were done with a continuum polarized conductor model (CPCM) included with the solvent methanol that has a dielectric constant of 32.7.

	E [au]	ZPE [au]	G [au]	E [au]
	B3LYP/BS1	B3LYP/BS1	B3LYP/BS1	B3LYP/BS2
Methylphenylindole	-634.1932731	0.239308	-633.993332	-634.3301322
HOBr	-2647.469917	0.012662	-2647.481463	-2649.942373
**Re**$_{MPI, Br}$	-3281.669595	0.253696	-3281.464228	-3284.274049
**TS**$_{MPI, Br}$	-3281.603766	0.252055	-3281.396558	-3284.215099
**Int**$_{MPI, Br}$	-3281.63444	0.251035	-3281.430996	-3284.240804
**ptTS**$_{MPI, Br}$	-3281.627464	0.25202	-3281.418861	-3284.242598
**Pr**$_{MPI, Br}$	-3281.721832	0.253106	-3281.516282	-3284.32553

Table S20. Relative energies, zero-point energies and free energies (in kcal mol^{-1}) of optimized geometries for the reaction of methylphenylindole with HOBr as obtained in Gaussian-09.

	ΔE	ΔE+ZPE	ΔG	ΔE	ΔE+ZPE	ΔG
	BS1	BS1	BS1	BS2	BS2	BS2
**Re**$_{MPI, Br}$	0.00	0.00	0.00	0.00	0.00	0.00
**TS**$_{MPI, Br}$	41.31	40.28	42.46	36.99	35.96	38.15
**Int**$_{MPI, Br}$	22.06	20.39	20.85	20.86	19.19	19.65
**ptTS**$_{MPI, Br}$	26.44	25.39	28.47	19.74	18.68	21.77
**Pr**$_{MPI, Br}$	-32.78	-33.15	-32.66	-32.30	-32.67	-32.19

16. DFT Calculations with explicit solvent molecules including a water matrix

Table S21. Absolute energies, zero-point energies and free energies (in a.u.) of optimized geometries for the reaction of 2-phenylindole with HOCl as obtained in Gaussian-09 surrounded by a water matrix made of 57 water molecules. All calculations were done with a continuum polarized conductor model (CPCM) included with the solvent Methanol that has a dielectric constant of 32.7.

	E [au]	ZPE [au]	G [au]	E [au]
	B3LYP/BS1	B3LYP/BS1	B3LYP/BS1	B3LYP/BS2
**Re**$_{2PI, Cl}$	-5487.620352	1.683648	-5486.135531	-5489.569434
**TS**$_{2PI, Cl}$	-5487.558367	1.679355	-5486.077886	-5489.511513
**Int**$_{2PI, Cl}$	-5487.687961	1.688301	-5486.193606	-5489.635409

Table S22. Relative energies, zero-point energies and free energies (in kcal mol^{-1}) of optimized geometries for the reaction of 2-phenylindole with HOCl as obtained in Gaussian-09 surrounded by a water matrix made of 57 water molecules.

	ΔE	ΔE+ZPE	ΔG	ΔE	ΔE+ZPE	ΔG
	BS1	BS1	BS1	BS2	BS2	BS2
**Re**$_{2PI, Cl}$	0.00	0.00	0.00	0.00	0.00	0.00
**TS**$_{2PI, Cl}$	38.90	36.20	36.17	36.35	33.65	33.62
**Int**$_{2PI, Cl}$	-10.46	-12.02	-12.01	-41.40	-42.96	-42.95

Table S23. Absolute energies, zero-point energies and free energies (in a.u.) of optimized geometries for the reaction of 2-phenylindole with HOBr as obtained in Gaussian-09 surrounded by a water matrix made of 57 water molecules. All calculations were done with a continuum polarized conductor model (CPCM) included with the solvent Methanol that has a dielectric constant of 32.7.

	E [au]	ZPE [au]	G [au]	E [au]
	B3LYP/BS1	B3LYP/BS1	B3LYP/BS1	B3LYP/BS2
**Re**$_{2PI, Br}$	-7599.155118	1.683303	-7597.671019	-7603.504411
**TS**$_{2PI, Br}$	-7599.097204	1.679398	-7597.618427	-7603.447672
**Int**$_{2PI, Br}$	-7599.199956	1.686764	-7597.705581	-7603.540965

Table S24. Relative energies, zero-point energies and free energies (in kcal mol^{-1}) of optimized geometries for the reaction of 2-phenylindole with HOBr as obtained in Gaussian-09 surrounded by a water matrix made of 57 water molecules.

	ΔE	ΔE+ZPE	ΔG	ΔE	ΔE+ZPE	ΔG
	BS1	BS1	BS1	BS2	BS2	BS2
**Re**$_{2PI, Br}$	0.00	0.00	0.00	0.00	0.00	0.00
**TS**$_{2PI, Br}$	36.34	33.89	33.00	35.60	33.15	32.26
**Int**$_{2PI, Br}$	-2.00	-3.60	-3.42	-22.94	-24.55	-24.36

Table S25. Absolute energies, zero-point energies and free energies (in a.u.) of optimized geometries for the reaction of methylphenylindole with HOBr as obtained in Gaussian-09 surrounded by a water matrix made of 57 water molecules. All calculations were done with a continuum polarized conductor model (CPCM) included with the solvent Methanol that has a dielectric constant of 32.7.

	E [au]	ZPE [au]	G [au]	E [au]
	B3LYP/BS1	B3LYP/BS1	B3LYP/BS1	B3LYP/BS2
**Re**$_{MPI, Br}$	-7638.455865	1.710891	-7636.947428	-7642.81251
**TS**$_{MPI, Br}$	-7638.384004	1.705947	-7636.882794	-7642.742033
**Int**$_{MPI, Br}$	-7638.476177	1.711092	-7636.964631	-7642.825256

Table S26. Relative energies, zero-point energies and free energies (in kcal mol^{-1}) of optimized geometries for the reaction of methylphenylindole with HOBr as obtained in Gaussian-09 surrounded by a water matrix made of 57 water molecules.

	ΔE	ΔE+ZPE	ΔG	ΔE	ΔE+ZPE	ΔG
	BS1	BS1	BS1	BS2	BS2	BS2
**Re**$_{MPI, Br}$	0.00	0.00	0.00	0.00	0.00	0.00
**TS**$_{MPI, Br}$	45.09	41.99	40.56	44.22	41.12	39.69
**Int**$_{MPI, Br}$	3.13	0.82	0.68	-8.00	-10.31	-10.45

17. Electric Field effects on halogen transfer transition state

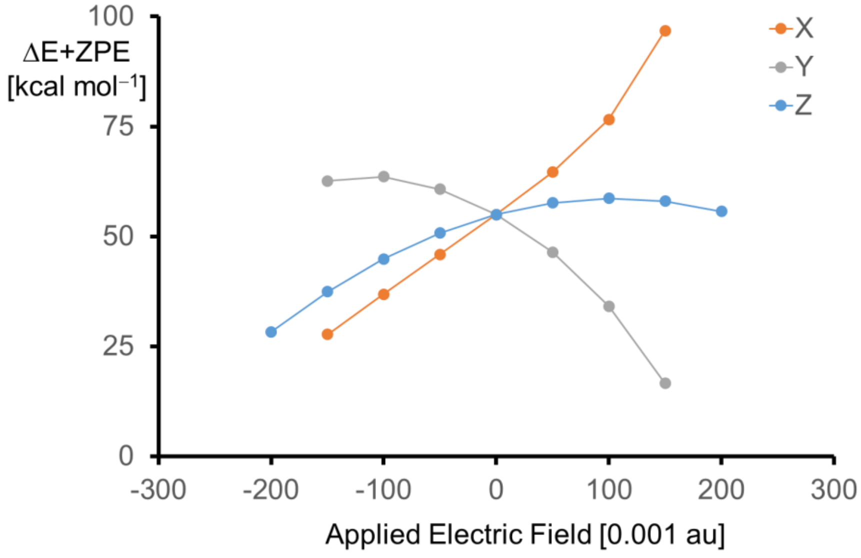

Figure S30. Electric field effect calculations on the halogen transfer barrier from HOBr to 2-phenylindole as calculated at UB3LYP/BS2 level of theory. The positive axis is as defined in Gaussian-09 and its direction is given with respect to the transition state structure.

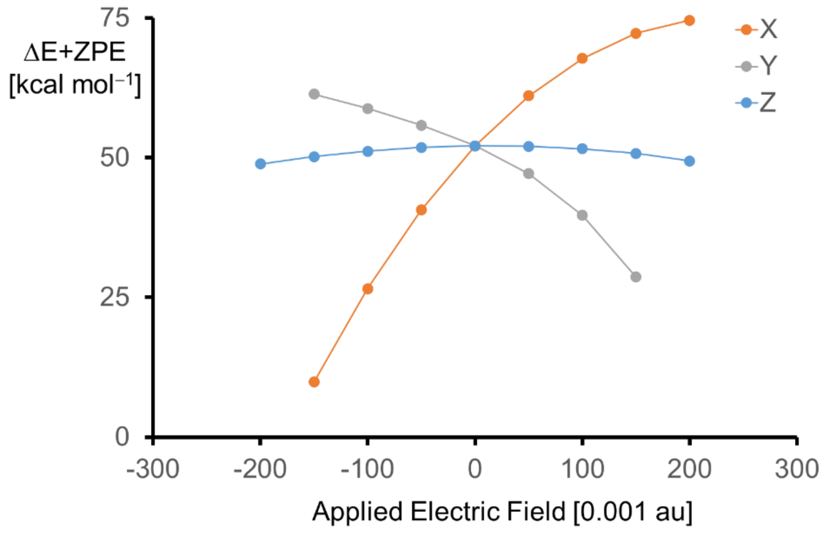

Figure S31. Electric field effect calculations on the halogen transfer barrier from HOCl to methylphenylindole as calculated at UB3LYP/BS2 level of theory. The positive axis is as defined in Gaussian-09 and its direction is given with respect to the transition state structure.

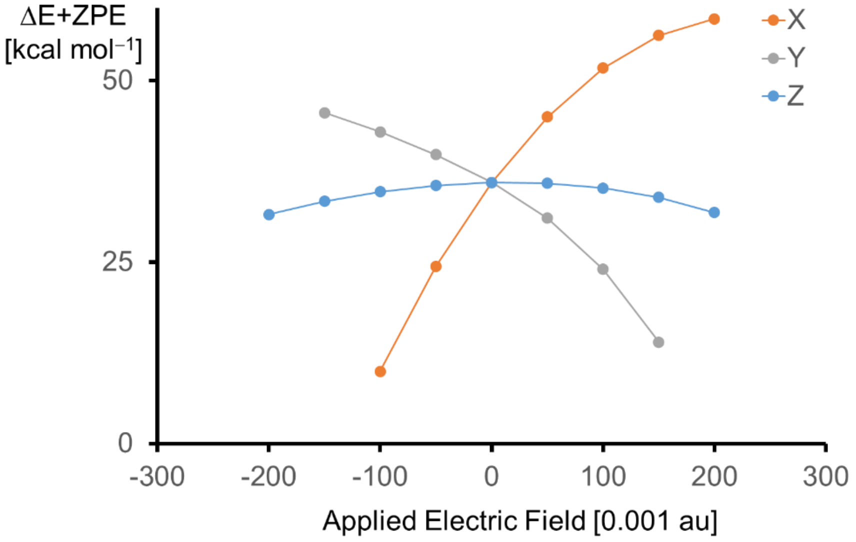

Figure S32. Electric field effect calculations on the halogen transfer barrier from HOBr to methylphenylindole as calculated at UB3LYP/BS2 level of theory. The positive axis is as defined in Gaussian-09 and its direction is given with respect to the transition state structure.

Table S27. Absolute energies (in a.u.) of optimized geometries with an applied electric field (over x, y and z axes) for the reaction of 2-phenylindole with HOCl as obtained in Gaussian-09. All calculations were done with a continuum polarized conductor model (CPCM) included with the solvent Methanol that has a dielectric constant of 32.7.

	E [au]								
	x-200	x-150	x-100	x-50	x +0	x+50	x+100	x+150	x+200
**Re**$_{2PI, Cl}$	-1131.08534103	-1131.06313463	-1131.04649619	-1131.03485938	-1131.02800315	-1131.02584852	-1131.02840367	-1131.03575589	-1131.04808992
**TS**$_{2PI, Cl}$	-1131.13365846	-1131.07611767	-1131.02904454	-1130.99231986	-1130.96581296	-1130.94930678	-1130.94260069	-1130.94563949	-1130.95869442
**Int**$_{2PI, Cl}$	-1131.09974580	-1131.07849106	-1131.06233360	-1131.05107050	-1131.04458840	-1131.04284324	-1131.04585335	-1131.05370301	-1131.06655994
**ptTS**$_{2PI, Cl}$	-1131.00567534	-1130.99432258	-1130.99101491	-1130.99527931	-1131.00682480	-1131.02542511	-1131.05091538	-1131.08322764	-1131.12245875
**Pr**$_{2PI, Cl}$	-1131.14624363	-1131.12569339	-1131.10993777	-1131.09883120	-1131.09229976	-1131.09032738	-1131.09295307	-1131.10027781	-1131.11248362

	E [au]								
	y-200	y-150	y-100	y-50	y +0	y+50	y+100	y+150	y+200
**Re**$_{2PI, Cl}$	-1131.22399874	-1131.05577090	-1131.03442807	-1131.02587234	-1131.02800315	-1131.04028198	-1131.06331009	-1131.10720702	-1131.23404819
**TS**$_{2PI, Cl}$	-1131.10571504	-1130.97788251	-1130.96023961	-1130.95656570	-1130.96581296	-1130.98889099	-1131.02860354	-1131.08882135	-1131.17165512
**Int**$_{2PI, Cl}$	-1131.14742577	-1131.07829486	-1131.05499469	-1131.04434690	-1131.04458840	-1131.05517603	-1131.07629161	-1131.10873637	-1131.17375996
**ptTS**$_{2PI, Cl}$	-1131.19032927	-1131.09802179	-1131.05350643	-1131.02382550	-1131.00682480	-1131.00173307	-1131.00859504	-1131.03199313	-1131.17932089
**Pr**$_{2PI, Cl}$	-1131.24712906	-1131.11965145	-1131.09914456	-1131.09061319	-1131.09229976	-1131.10372793	-1131.12512370	-1131.15756840	-1131.24655411

	E [au]								
	z-200	z-150	z-100	z-50	z +0	z+50	z+100	z+150	z+200
**Re**$_{2PI, Cl}$	-1131.06644795	-1131.04643775	-1131.03352436	-1131.02742890	-1131.02800315	-1131.03518326	-1131.04897483	-1131.06945432	-1131.09678778
**TS**$_{2PI, Cl}$	-1131.03234485	-1131.00523165	-1130.98520384	-1130.97209428	-1130.96581296	-1130.96633130	-1130.97368082	-1130.98796224	-1131.00936999
**Int**$_{2PI, Cl}$	-1131.16761821	-1131.12610537	-1131.09185958	-1131.06470871	-1131.04458840	-1131.03156071	-1131.02594492	-1131.02882705	-1131.04449948
**ptTS**$_{2PI, Cl}$	-1131.04513677	-1131.02696830	-1131.01464803	-1131.00796680	-1131.00682480	-1131.01120174	-1131.02115808	-1131.03687882	-1131.05899002
**Pr**$_{2PI, Cl}$	-1131.17528120	-1131.14390558	-1131.11975428	-1131.10258884	-1131.09229976	-1131.08886555	-1131.09234524	-1131.10289185	-1131.12080399

Table S28. Relative energies (in kcal mol-1) of optimized geometries with an applied electric field (over x, y and z axes) for the reaction of 2-phenylindole with HOCl as obtained in Gaussian-09.

	E [au]								
	x-200	x-150	x-100	x-50	x +0	x+50	x+100	x+150	x+200
**Re**$_{2PI, Cl}$	9.04	9.64	9.94	10.17	10.41	10.66	10.95	11.26	11.59
**TS**$_{2PI, Cl}$	-21.28	1.49	20.89	36.87	49.43	58.69	64.79	67.81	67.69
**Int**$_{2PI, Cl}$	0.00	0.00	0.00	0.00	0.00	0.00	0.00	0.00	0.00
**ptTS**$_{2PI, Cl}$	59.03	52.82	44.75	35.01	23.70	10.93	-3.18	-18.53	-35.08
**Pr**$_{2PI, Cl}$	-29.18	-29.62	-29.87	-29.97	-29.94	-29.80	-29.56	-29.23	-28.82

	E [au]								
	y-200	y-150	y-100	y-50	y +0	y+50	y+100	y+150	y+200
**Re**$_{2PI, Cl}$	-48.05	14.13	12.91	11.59	10.41	9.35	8.15	0.96	-37.83
**TS**$_{2PI, Cl}$	26.17	63.01	59.46	55.08	49.43	41.59	29.92	12.50	1.32
**Int**$_{2PI, Cl}$	0.00	0.00	0.00	0.00	0.00	0.00	0.00	0.00	0.00
**ptTS**$_{2PI, Cl}$	-26.92	-12.38	0.93	12.88	23.70	33.54	42.48	48.16	-3.49
**Pr**$_{2PI, Cl}$	-62.56	-25.95	-27.70	-29.03	-29.94	-30.47	-30.64	-30.64	-45.68

	E [au]								
	z-200	z-150	z-100	z-50	z +0	z+50	z+100	z+150	z+200
**Re**$_{2PI, Cl}$	63.48	49.99	36.61	23.39	10.41	-2.27	-14.45	-25.49	-32.81
**TS**$_{2PI, Cl}$	84.88	75.85	66.93	58.12	49.43	40.93	32.80	25.64	22.04
**Int**$_{2PI, Cl}$	0.00	0.00	0.00	0.00	0.00	0.00	0.00	0.00	0.00
**ptTS**$_{2PI, Cl}$	76.86	62.21	48.45	35.61	23.70	12.78	3.00	-5.05	-9.09
**Pr**$_{2PI, Cl}$	-4.81	-11.17	-17.50	-23.77	-29.94	-35.96	-41.67	-46.48	-47.88

Table S29. Absolute energies (in a.u.) of optimized geometries with an applied electric field (over x, y and z axes) for the reaction of 2-phenylindole with HOBr as obtained in Gaussian-09. All calculations were done with a continuum polarized conductor model (CPCM) included with the solvent Methanol that has a dielectric constant of 32.7.

	E [au]								
	x-200	x-150	x-100	x-50	x +0	x+50	x+100	x+150	x+200
**Re**$_{2PI, Br}$	-3245.15511098	-3245.01902887	-3244.98866371	-3244.96970862	-3244.96134761	-3244.96380927	-3244.97943648	-3245.01744999	-3245.15003327
**TS**$_{2PI, Br}$	-3245.05839450	-3245.00056645	-3244.95559506	-3244.92219783	-3244.89941459	-3244.88652253	-3244.88306958	-3244.88891454	-3244.90436718
**Int**$_{2PI, Br}$	-3245.02415532	-3245.00180878	-3244.98494692	-3244.97331425	-3244.96676575	-3244.96523747	-3244.96873587	-3244.97733997	-3244.99121935
**ptTS**$_{2 PI, Br}$	-3244.92907060	-3244.91708289	-3244.91339167	-3244.91751071	-3244.92914294	-3244.94806287	-3244.97411281	-3245.00723953	-3245.04756501
**Pr**$_{2PI, Br}$	-3245.05127174	-3245.03364991	-3245.02120637	-3245.01366490	-3245.01090128	-3245.01287509	-3245.01961487	-3245.03122391	-3245.04791019

	E [au]								
	y-200	y-150	y-100	y-50	y +0	y+50	y+100	y+150	y+200
**Re**$_{2PI, Br}$	-3245.01106666	-3244.98679702	-3244.97058610	-3244.96210590	-3244.96134761	-3244.96860559	-3244.98459656	-3245.01074295	-3245.05063207
**TS**$_{2PI, Br}$	-3245.01091451	-3244.91269549	-3244.89499243	-3244.89106944	-3244.89941459	-3244.92033386	-3244.95592861	-3245.00998694	-3245.08564980
**Int**$_{2PI, Br}$	-3245.07106049	-3245.00093520	-3244.97725596	-3244.96644683	-3244.96676575	-3244.97767698	-3244.99937632	-3245.03269243	-3245.13432142
**ptTS**$_{2 PI, Br}$	-3245.11594890	-3245.02257211	-3244.97704785	-3244.94660489	-3244.92914294	-3244.92391706	-3244.93098444	-3244.95497133	-3245.10366850
**Pr**$_{2PI, Br}$	-3245.17667218	-3245.02933507	-3245.01126186	-3245.00572334	-3245.01090128	-3245.02625796	-3245.05192254	-3245.08874513	-3245.19191305

	E [au]								
	z-200	z-150	z-100	z-50	z +0	z+50	z+100	z+150	z+200
**Re**$_{2PI, Br}$	-3244.99094688	-3244.97606911	-3244.96627564	-3244.96139839	-3244.96134761	-3244.96609594	-3244.97567416	-3244.99017684	-3245.00978074
**TS**$_{2PI, Br}$	-3244.97150509	-3244.94213345	-3244.92044509	-3244.90623839	-3244.89941459	-3244.89995064	-3244.90789921	-3244.92340755	-3244.94677296
**Int**$_{2PI, Br}$	-3245.09563668	-3245.05196452	-3245.01604874	-3244.98767571	-3244.96676575	-3244.95339754	-3244.94803128	-3244.95260803	-3244.97684123
**ptTS**$_{2 PI, Br}$	-3244.97342977	-3244.95292779	-3244.93884020	-3244.93094142	-3244.92914294	-3244.93346542	-3244.94406610	-3244.96143273	-3244.99061457
**Pr**$_{2PI, Br}$	-3245.09807051	-3245.06492000	-3245.03957958	-3245.02162476	-3245.01090128	-3245.00736354	-3245.01105495	-3245.02212216	-3245.04088998

Table S30. Relative energies (in kcal mol[-1]) of optimized geometries with an applied electric field (over x, y and z axes) for the reaction of 2-phenylindole with HOBr as obtained in Gaussian-09.

	E [au]								
	x-200	x-150	x-100	x-50	x +0	x+50	x+100	x+150	x+200
**Re**$_{2PI, Br}$	-82.17	-10.81	-2.33	2.26	3.40	0.90	-6.71	-25.17	-99.66
**TS**$_{2PI, Br}$	-21.49	0.78	18.42	32.08	42.26	49.39	53.76	55.49	54.50
**Int**$_{2PI, Br}$	0.00	0.00	0.00	0.00	0.00	0.00	0.00	0.00	0.00
**ptTS**$_{2PI, Br}$	59.67	53.17	44.90	35.02	23.61	10.78	-3.37	-18.76	-35.36
**Pr**$_{2PI, Br}$	-17.02	-19.98	-22.75	-25.32	-27.70	-29.89	-31.93	-33.81	-35.57

	E [au]								
	y-200	y-150	y-100	y-50	y +0	y+50	y+100	y+150	y+200
**Re**$_{2PI, Br}$	37.65	8.87	4.19	2.72	3.40	5.69	9.27	13.77	52.52
**TS**$_{2PI, Br}$	37.74	55.37	51.62	47.30	42.26	35.98	27.26	14.25	30.54
**Int**$_{2PI, Br}$	0.00	0.00	0.00	0.00	0.00	0.00	0.00	0.00	0.00
**ptTS**$_{2PI, Br}$	-28.17	-13.58	0.13	12.45	23.61	33.73	42.92	48.77	19.23
**Pr**$_{2PI, Br}$	-66.27	-17.82	-21.34	-24.65	-27.70	-30.48	-32.97	-35.17	-36.14

	E [au]								
	z-200	z-150	z-100	z-50	z +0	z+50	z+100	z+150	z+200
**Re**$_{2PI, Br}$	65.69	47.62	31.23	16.49	3.40	-7.97	-17.35	-23.57	-20.67
**TS**$_{2PI, Br}$	77.89	68.92	59.99	51.10	42.26	33.54	25.18	18.32	18.87
**Int**$_{2PI, Br}$	0.00	0.00	0.00	0.00	0.00	0.00	0.00	0.00	0.00
**ptTS**$_{2PI, Br}$	76.68	62.15	48.45	35.60	23.61	12.51	2.49	-5.54	-8.64
**Pr**$_{2PI, Br}$	-1.53	-8.13	-14.77	-21.30	-27.70	-33.86	-39.55	-43.62	-40.19

Table S31. Absolute energies (in a.u.) of optimized geometries with an applied electric field (over x, y and z axes) for the reaction of methylphenylindole with HOCl as obtained in Gaussian-09. All calculations were done with a continuum polarized conductor model (CPCM) included with the solvent Methanol that has a dielectric constant of 32.7.

	E [au]								
	x-200	x-150	x-100	x-50	x +0	x+50	x+100	x+150	x+200
**Re**$_{MPI, Cl}$	-1170.39979794	-1170.37303993	-1170.35564861	-1170.34468295	-1170.33939907	-1170.33960649	-1170.34529098	-1170.35655216	-1170.37361831
**TS**$_{MPI, Cl}$	-1170.43680128	-1170.38141447	-1170.33738653	-1170.30394011	-1170.28041905	-1170.26631859	-1170.26133564	-1170.26541152	-1170.27883336
**Int**$_{MPI, Cl}$	-1170.46563271	-1170.40515313	-1170.35525458	-1170.31607824	-1170.28710999	-1170.26791605	-1170.25816844	-1170.25765126	-1170.26630553
**ptTS**$_{MPI, Cl}$	-1170.31502544	-1170.30405341	-1170.30154547	-1170.30703985	-1170.32021583	-1170.34081383	-1170.36864277	-1170.40362815	-1170.44589194
**Pr**$_{MPI, Cl}$	-1170.44799646	-1170.42952651	-1170.41624454	-1170.40797694	-1170.40463463	-1170.40619345	-1170.41269019	-1170.42423076	-1170.44101571

	E [au]								
	y-200	y-150	y-100	y-50	y +0	y+50	y+100	y+150	y+200
**Re**$_{MPI, Cl}$	-1170.52316875	-1170.37013358	-1170.34857385	-1170.33890109	-1170.33939907	-1170.34965239	-1170.36994258	-1170.40324367	-1170.50802493
**TS**$_{MPI, Cl}$	-1170.39663752	-1170.29636021	-1170.27891803	-1170.27400388	-1170.28041905	-1170.29856483	-1170.33079018	-1170.38168211	-1170.45493588
**Int**$_{MPI, Cl}$	-1170.38982546	-1170.31064575	-1170.29058973	-1170.28227006	-1170.28710999	-1170.30641826	-1170.34273448	-1170.39862829	-1170.47507591
**ptTS**$_{MPI, Cl}$	-1170.51877736	-1170.41991861	-1170.37249394	-1170.33985867	-1170.32021583	-1170.31304194	-1170.31853594	-1170.33768079	-1170.48733067
**Pr**$_{MPI, Cl}$	-1170.56134478	-1170.43957045	-1170.41717383	-1170.40597178	-1170.40463463	-1170.41285632	-1170.43085334	-1170.45951455	-1170.55802322

	E [au]								
	z-200	z-150	z-100	z-50	z +0	z+50	z+100	z+150	z+200
**Re**$_{MPI, Cl}$	-1170.38744979	-1170.36450199	-1170.34901050	-1170.34068764	-1170.33939907	-1170.34510230	-1170.35782961	-1170.37768996	-1170.40488921
**TS**$_{MPI, Cl}$	-1170.33359179	-1170.30855700	-1170.29151452	-1170.28217608	-1170.28041905	-1170.28621841	-1170.29963744	-1170.32084433	-1170.35016688
**Int**$_{MPI, Cl}$	-1170.27882155	-1170.26676505	-1170.26430786	-1170.27112744	-1170.28710999	-1170.31228433	-1170.34680528	-1170.39093749	-1170.44506256
**ptTS**$_{MPI, Cl}$	-1170.35445998	-1170.33645542	-1170.32488596	-1170.31951227	-1170.32021583	-1170.32696227	-1170.33979822	-1170.35888548	-1170.38473411
**Pr**$_{MPI, Cl}$	-1170.43772662	-1170.41715985	-1170.40512128	-1170.40102835	-1170.40463463	-1170.41583910	-1170.43464649	-1170.46116683	-1170.49565536

Table S32. Relative energies (in kcal mol[-1]) of optimized geometries with an applied electric field (over x, y and z axes) for the reaction of methylphenylindole with HOCl as obtained in Gaussian-09.

	E [au]								
	x-200	x-150	x-100	x-50	x +0	x+50	x+100	x+150	x+200
**Re**$_{MPI, Cl}$	41.31	20.15	-0.25	-17.95	-32.81	-44.99	-54.67	-62.06	-67.34
**TS**$_{MPI, Cl}$	18.09	14.90	11.21	7.62	4.20	1.00	-1.99	-4.87	-7.86
**Int**$_{MPI, Cl}$	0.00	0.00	0.00	0.00	0.00	0.00	0.00	0.00	0.00
**ptTS**$_{MPI, Cl}$	94.51	63.44	33.70	5.67	-20.77	-45.74	-69.32	-91.60	-112.69
**Pr**$_{MPI, Cl}$	11.07	-15.29	-38.27	-57.67	-73.75	-86.77	-96.96	-104.53	-109.63

	E [au]								
	y-200	y-150	y-100	y-50	y +0	y+50	y+100	y+150	y+200
**Re**$_{MPI, Cl}$	-83.67	-37.33	-36.39	-35.54	-32.81	-27.13	-17.07	-2.90	-20.68
**TS**$_{MPI, Cl}$	-4.27	8.96	7.32	5.19	4.20	4.93	7.50	10.63	12.64
**Int**$_{MPI, Cl}$	0.00	0.00	0.00	0.00	0.00	0.00	0.00	0.00	0.00
**ptTS**$_{MPI, Cl}$	-80.92	-68.57	-51.39	-36.14	-20.77	-4.16	15.18	38.24	-7.69
**Pr**$_{MPI, Cl}$	-107.63	-80.90	-79.43	-77.62	-73.75	-66.79	-55.29	-38.21	-52.05

	E [au]								
	z-200	z-150	z-100	z-50	z +0	z+50	z+100	z+150	z+200
**Re**$_{MPI, Cl}$	-68.16	-61.33	-53.15	-43.65	-32.81	-20.59	-6.92	8.31	25.21
**TS**$_{MPI, Cl}$	-34.37	-26.22	-17.07	-6.93	4.20	16.36	29.60	43.98	59.55
**Int**$_{MPI, Cl}$	0.00	0.00	0.00	0.00	0.00	0.00	0.00	0.00	0.00
**ptTS**$_{MPI, Cl}$	-47.46	-43.73	-38.01	-30.36	-20.77	-9.21	4.40	20.11	37.86
**Pr**$_{MPI, Cl}$	-99.71	-94.37	-88.36	-81.51	-73.75	-64.98	-55.12	-44.07	-31.75

Table S33. Absolute energies (in a.u.) of optimized geometries with an applied electric field (over x, y and z axes) for the reaction of methylphenylindole with HOBr as obtained in Gaussian-09. All calculations were done with a continuum polarized conductor model (CPCM) included with the solvent Methanol that has a dielectric constant of 32.7.

	E [au]								
	x-200	x-150	x-100	x-50	x +0	x+50	x+100	x+150	x+200
**Re**$_{MPI, Br}$	-3284.34027879	-3284.30782290	-3284.28968067	-3284.27885002	-3284.27404917	-3284.27504525	-3284.28182286	-3284.29449076	-3284.31330220
**TS**$_{MPI, Br}$	-3284.37629086	-3284.31767456	-3284.27218601	-3284.23834967	-3284.21509875	-3284.20170508	-3284.19776936	-3284.20322112	-3284.21844336
**Int**$_{MPI, Br}$	-3284.38100460	-3284.32332102	-3284.27665961	-3284.24143867	-3284.21685904	-3284.20209333	-3284.19654109	-3284.19985651	-3284.21197225
**ptTS**$_{MPI, Br}$	-3284.23906514	-3284.22731094	-3284.22427610	-3284.22947968	-3284.24259835	-3284.26337929	-3284.29164470	-3284.32733951	-3284.37061487
**Pr**$_{MPI, Br}$	-3284.34655592	-3284.33015922	-3284.32141102	-3284.31992164	-3284.32552967	-3284.33818346	-3284.35792270	-3284.38489679	-3284.41945805

	E [au]								
	y-200	y-150	y-100	y-50	y +0	y+50	y+100	y+150	y+200
**Re**$_{MPI, Br}$	-3284.44206501	-3284.30593510	-3284.28395856	-3284.27386807	-3284.27404917	-3284.28409193	-3284.30423861	-3284.33732378	-3284.43553247
**TS**$_{MPI, Br}$	-3284.34218572	-3284.23167045	-3284.21385993	-3284.20878994	-3284.21509875	-3284.23297942	-3284.26432706	-3284.31340229	-3284.38488843
**Int**$_{MPI, Br}$	-3284.31420091	-3284.23368625	-3284.21072492	-3284.20863374	-3284.21685904	-3284.23914782	-3284.27764631	-3284.33460577	-3284.41051850
**ptTS**$_{MPI, Br}$	-3284.44409278	-3284.34434653	-3284.29601551	-3284.26267260	-3284.24259835	-3284.23531675	-3284.24105700	-3284.26079034	-3284.41236674
**Pr**$_{MPI, Br}$	-3284.50091214	-3284.38744498	-3284.35610949	-3284.33582126	-3284.32552967	-3284.32497594	-3284.33434039	-3284.35436701	-3284.44018472

	E [au]								
	z-200	z-150	z-100	z-50	z +0	z+50	z+100	z+150	z+200
**Re**$_{MPI, Br}$	-3284.32602672	-3284.30159112	-3284.28497445	-3284.27584648	-3284.27404917	-3284.27952919	-3284.29231835	-3284.31253556	-3284.34040988
**TS**$_{MPI, Br}$	-3284.27408436	-3284.24679025	-3284.22806599	-3284.21754251	-3284.21509875	-3284.22073520	-3284.23456282	-3284.25683455	-3284.28805404
**Int**$_{MPI, Br}$	-3284.22141684	-3284.20551269	-3284.19962540	-3284.20342719	-3284.21685904	-3284.24005739	-3284.27337905	-3284.31740394	-3284.37287538
**ptTS**$_{MPI, Br}$	-3284.28360456	-3284.26303007	-3284.24948609	-3284.24271255	-3284.24259835	-3284.24914438	-3284.26248252	-3284.28303736	-3284.31523760
**Pr**$_{MPI, Br}$	-3284.35655571	-1170.41715985	-1170.40512128	-1170.40102835	-3284.32552967	-3284.33329981	-3284.34721096	-3284.36742881	-3284.39434475

Table S34. Relative energies (in kcal mol^{-1}) of optimized geometries with an applied electric field (over x, y and z axes) for the reaction of methylphenylindole with HOBr as obtained in Gaussian-09.

	E [au]								
	x-200	x-150	x-100	x-50	x +0	x+50	x+100	x+150	x+200
**Re**$_{MPI, Br}$	25.56	9.73	-8.17	-23.48	-35.89	-45.78	-53.51	-59.38	-63.58
**TS**$_{MPI, Br}$	2.96	3.54	2.81	1.94	1.10	0.24	-0.77	-2.11	-4.06
**Int**$_{MPI, Br}$	0.00	0.00	0.00	0.00	0.00	0.00	0.00	0.00	0.00
**ptTS**$_{MPI, Br}$	89.07	60.25	32.87	7.50	-16.15	-38.46	-59.68	-80.00	-99.55
**Pr**$_{MPI, Br}$	21.62	-4.29	-28.08	-49.25	-68.19	-85.40	-101.27	-116.11	-130.20

	E [au]								
	y-200	y-150	y-100	y-50	y +0	y+50	y+100	y+150	y+200
**Re**$_{MPI, Br}$	-80.23	-45.34	-45.95	-40.93	-35.89	-28.20	-16.69	-1.71	-15.70
**TS**$_{MPI, Br}$	-17.56	1.26	-1.97	-0.10	1.10	3.87	8.36	13.31	16.08
**Int**$_{MPI, Br}$	0.00	0.00	0.00	0.00	0.00	0.00	0.00	0.00	0.00
**ptTS**$_{MPI, Br}$	-81.51	-69.44	-53.52	-33.91	-16.15	2.40	22.96	46.32	-1.16
**Pr**$_{MPI, Br}$	-117.16	-96.48	-91.23	-79.81	-68.19	-53.86	-35.58	-12.40	-18.62

	E [au]								
	z-200	z-150	z-100	z-50	z +0	z+50	z+100	z+150	z+200
**Re**$_{MPI, Br}$	-65.64	-60.29	-53.56	-45.44	-35.89	-24.77	-11.88	3.05	20.37
**TS**$_{MPI, Br}$	-33.05	-25.90	-17.85	-8.86	1.10	12.12	24.36	38.01	53.23
**Int**$_{MPI, Br}$	0.00	0.00	0.00	0.00	0.00	0.00	0.00	0.00	0.00
**ptTS**$_{MPI, Br}$	-39.02	-36.09	-31.29	-24.65	-16.15	-5.70	6.84	21.57	36.17
**Pr**$_{MPI, Br}$	-84.80	-83.83	-80.76	-75.57	-68.19	-58.51	-46.33	-31.39	-13.47

18. Cartesian Coordinates of DFT models

17.1 Implicit solvent model calculations

Re$_{2PI,Cl}$:

6	-4.972185000	-0.699541000	6.726547000
6	-5.059511000	-1.762848000	5.807153000
6	-4.671868000	-1.536666000	4.453154000
6	-4.221780000	-0.290495000	4.002114000
6	-4.150529000	0.744838000	4.936856000
6	-4.522906000	0.539140000	6.286791000
6	-5.474118000	-3.129095000	5.893850000
6	-5.333417000	-3.692498000	4.638824000
7	-4.831722000	-2.724921000	3.777551000
1	-5.257515000	-0.846473000	7.764856000
1	-3.925552000	-0.136875000	2.968432000
1	-3.782973000	1.718936000	4.625674000
1	-4.451753000	1.366789000	6.986733000
1	-5.800059000	-3.648196000	6.784631000
6	-5.605792000	-5.060616000	4.187404000
6	-6.523176000	-5.866110000	4.887835000
6	-4.962309000	-5.603979000	3.060320000
6	-6.783599000	-7.171111000	4.476374000
6	-5.232107000	-6.907928000	2.645609000
6	-6.142144000	-7.698053000	3.351280000
1	-7.045847000	-5.457270000	5.747480000
1	-4.226572000	-5.017259000	2.517186000
1	-7.497350000	-7.775292000	5.029626000
1	-4.722181000	-7.308777000	1.773982000
1	-6.350408000	-8.714100000	3.028322000
17	-7.677748000	0.874898000	2.702943000
8	-7.115897000	1.566925000	4.181076000
1	-6.222891000	1.164961000	4.291105000
1	-4.749032000	-2.833005000	2.777049000

TS$_{2PI,Cl}$:

6	-3.883581000	-0.296048000	6.684562000
6	-4.322263000	-1.354866000	5.848687000
6	-3.966688000	-1.349946000	4.469371000
6	-3.174453000	-0.361074000	3.904362000
6	-2.766865000	0.671779000	4.750093000
6	-3.114134000	0.706347000	6.124791000
6	-5.145385000	-2.468577000	6.053305000
6	-5.226463000	-3.189295000	4.810057000
7	-4.523314000	-2.488728000	3.894028000
1	-4.152825000	-0.279553000	7.735936000
1	-2.908872000	-0.369049000	2.853224000
1	-2.162346000	1.476251000	4.342171000
1	-2.766989000	1.532366000	6.736564000
1	-5.576302000	-2.796623000	6.986111000
6	-5.855393000	-4.468889000	4.553947000
6	-6.748383000	-5.012501000	5.502459000
6	-5.587612000	-5.197423000	3.374938000
6	-7.356762000	-6.241543000	5.272501000
6	-6.198114000	-6.425908000	3.153181000
6	-7.085000000	-6.951851000	4.099506000
1	-6.981239000	-4.459095000	6.404550000
1	-4.884671000	-4.820418000	2.638591000
1	-8.045679000	-6.645664000	6.007770000
1	-5.978417000	-6.978617000	2.245053000
1	-7.558503000	-7.913174000	3.923697000
17	-7.705257000	-1.544681000	5.258760000
8	-6.064666000	0.061158000	3.718894000
1	-5.660456000	0.437057000	4.519451000
1	-4.439659000	-2.723185000	2.925507000

Int$_{2PI,Cl}$:

6	-3.156817000	0.114412000	7.675507000
6	-3.715523000	-0.869783000	6.874064000
6	-3.970004000	-0.610967000	5.524442000
6	-3.697700000	0.607299000	4.919225000
6	-3.136831000	1.598306000	5.733812000
6	-2.868728000	1.355875000	7.086798000
6	-4.149792000	-2.285058000	7.140335000
6	-4.643392000	-2.743143000	5.761015000
7	-4.510399000	-1.760206000	4.907246000
1	-2.954357000	-0.060022000	8.727716000
1	-3.922073000	0.758767000	3.868857000
1	-2.906148000	2.570280000	5.308586000
1	-2.431933000	2.142746000	7.693901000
1	-3.355196000	-2.929976000	7.520554000
6	-5.146884000	-4.058210000	5.412477000
6	-5.115554000	-5.109466000	6.351940000
6	-5.663151000	-4.293405000	4.116188000
6	-5.591104000	-6.370028000	6.005647000
6	-6.138900000	-5.557494000	3.787466000
6	-6.106189000	-6.595661000	4.725841000
1	-4.717789000	-4.945880000	7.347311000
1	-5.671496000	-3.476930000	3.386044000
1	-5.559658000	-7.175750000	6.732468000
1	-6.536741000	-5.738864000	2.793176000
1	-6.478860000	-7.580499000	4.458843000
17	-5.491595000	-2.374366000	8.384618000
8	-5.307133000	-1.510190000	2.633007000
1	-6.236839000	-1.257250000	2.760122000
1	-4.851183000	-1.701275000	3.919058000

ptTS$_{2PI,Cl}$:

6	-2.745809000	0.245437000	6.693917000
6	-3.709231000	-0.669573000	6.286908000
6	-4.817146000	-0.227198000	5.549644000
6	-5.000721000	1.100500000	5.184586000
6	-4.018614000	2.009028000	5.595227000
6	-2.908060000	1.590779000	6.338738000
6	-3.840825000	-2.145945000	6.478312000
6	-5.116283000	-2.484010000	5.746812000
7	-5.633322000	-1.341266000	5.265899000
1	-1.887909000	-0.073850000	7.277991000
1	-5.861843000	1.419862000	4.606549000
1	-4.123488000	3.056662000	5.329784000
1	-2.162253000	2.318565000	6.643312000
1	-3.066023000	-2.865537000	6.040324000
6	-5.503501000	-3.802480000	5.416036000
6	-4.749486000	-4.889778000	5.963741000
6	-6.497615000	-4.071074000	4.427508000
6	-5.050199000	-6.204042000	5.542232000
6	-6.746552000	-5.358538000	4.014455000
6	-6.012957000	-6.432799000	4.580175000
1	-4.189153000	-4.752131000	6.871359000
1	-7.051056000	-3.254070000	3.973533000
1	-4.517415000	-7.036039000	5.992436000
1	-7.499613000	-5.554239000	3.257726000

1	-6.231074000	-7.449547000	4.264137000
17	-3.920803000	-2.579205000	8.277306000
8	-2.599769000	-4.347020000	5.265068000
1	-2.114898000	-4.772435000	5.994570000
1	-6.456351000	-1.286791000	4.677977000

Pro$_{2PI,Cl}$:

6	-4.343939000	-0.594990000	6.930305000
6	-4.685929000	-1.947457000	7.113048000
6	-4.941882000	-2.434074000	8.425245000
6	-4.841827000	-1.609442000	9.553163000
6	-4.489741000	-0.278425000	9.347541000
6	-4.247547000	0.224805000	8.048975000
6	-4.863080000	-3.066847000	6.241541000
6	-5.219614000	-4.174798000	6.992238000
7	-5.279603000	-3.763348000	8.317454000
1	-4.151297000	-0.205779000	5.934685000
1	-5.028664000	-1.997534000	10.550169000
1	-4.400438000	0.386280000	10.201787000
1	-3.978683000	1.269986000	7.926641000
1	-6.979208000	0.031754000	7.404437000
6	-5.544481000	-5.556903000	6.620589000
6	-6.534055000	-6.263998000	7.329469000
6	-4.870669000	-6.209051000	5.572343000
6	-6.840172000	-7.582869000	6.997836000
6	-5.184540000	-7.526247000	5.240852000
6	-6.168527000	-8.218929000	5.951033000
1	-7.083436000	-5.772197000	8.127380000
1	-4.088437000	-5.689328000	5.030132000
1	-7.610329000	-8.110461000	7.553455000
1	-4.650864000	-8.015638000	4.430891000
1	-6.409248000	-9.245897000	5.691405000
17	-4.747331000	-2.975135000	4.504176000
8	-7.691941000	-0.140527000	8.040109000
1	-7.195624000	-0.382199000	8.838266000
1	-5.384829000	-4.398461000	9.096256000

Re$_{2PI,Br}$:

6	-4.389563000	-0.936256000	-0.441398000
6	-3.005507000	-0.835895000	-0.200427000
6	-2.480767000	0.393045000	0.296235000
6	-3.289647000	1.511571000	0.528430000
6	-4.650539000	1.383592000	0.269666000
6	-5.195721000	0.170705000	-0.206825000
6	-1.896650000	-1.731926000	-0.340761000
6	-0.761491000	-1.058206000	0.065970000
7	-1.124680000	0.219540000	0.478514000
1	-4.817291000	-1.864705000	-0.811204000
1	-2.870371000	2.442646000	0.899445000
1	-5.306984000	2.232478000	0.440268000
1	-6.264405000	0.106915000	-0.393291000
1	-1.934948000	-2.765278000	-0.658175000
6	0.629827000	-1.510037000	0.131188000
6	1.113750000	-2.468031000	-0.775730000
6	1.515762000	-1.002342000	1.107370000
6	2.437520000	-2.901863000	-0.714551000
6	2.847386000	-1.431649000	1.154319000
6	3.312944000	-2.381204000	0.243027000
1	0.451156000	-2.857777000	-1.542726000
1	1.142871000	-0.330708000	1.875330000
1	2.790760000	-3.641482000	-1.427677000
1	3.509871000	-1.032929000	1.917129000

1	4.345438000	-2.715394000	0.280634000
35	2.233079000	1.567154000	-0.103686000
8	2.475554000	3.326752000	-0.741722000
1	2.199912000	3.245001000	-1.673775000
1	-0.459270000	0.971018000	0.608161000

TS$_{2PI,Br}$:

6	-3.819370000	-0.313471000	6.700463000
6	-4.302796000	-1.334226000	5.862533000
6	-3.970192000	-1.325065000	4.487291000
6	-3.192936000	-0.330969000	3.910026000
6	-2.726766000	0.675421000	4.762217000
6	-3.027589000	0.682390000	6.139046000
6	-5.172259000	-2.443654000	6.064326000
6	-5.238143000	-3.168656000	4.823081000
7	-4.553030000	-2.455730000	3.910479000
1	-4.065660000	-0.303087000	7.757817000
1	-2.959074000	-0.329126000	2.850936000
1	-2.114827000	1.472078000	4.349915000
1	-2.639390000	1.480214000	6.764078000
1	-5.499213000	-2.834809000	7.014844000
6	-5.865010000	-4.451702000	4.554154000
6	-6.826495000	-4.967081000	5.447398000
6	-5.513029000	-5.209660000	3.418617000
6	-7.423085000	-6.199682000	5.201808000
6	-6.112940000	-6.442054000	3.181106000
6	-7.069531000	-6.941357000	4.070650000
1	-7.133507000	-4.379310000	6.304894000
1	-4.749878000	-4.853595000	2.733113000
1	-8.168372000	-6.581793000	5.892908000
1	-5.827685000	-7.017968000	2.306001000
1	-7.533998000	-7.905245000	3.884559000
35	-7.606594000	-1.470620000	5.639865000
8	-6.279787000	-0.244709000	3.554615000
1	-5.805458000	0.293549000	4.210477000
1	-4.505865000	-2.667987000	2.934404000

Int$_{2PI,Br}$:

6	-3.103379000	0.094635000	7.660746000
6	-3.692071000	-0.877965000	6.865502000
6	-3.992470000	-0.600262000	5.528287000
6	-3.735188000	0.625149000	4.930428000
6	-3.142212000	1.602598000	5.738199000
6	-2.828995000	1.341309000	7.078532000
6	-4.116879000	-2.291299000	7.122790000
6	-4.652114000	-2.735806000	5.763940000
7	-4.551917000	-1.742059000	4.916402000
1	-2.870779000	-0.094477000	8.704148000
1	-3.992009000	0.791273000	3.889733000
1	-2.921052000	2.578964000	5.318016000
1	-2.368115000	2.118557000	7.680228000
1	-3.345258000	-2.950597000	7.519236000
6	-5.148397000	-4.053726000	5.411516000
6	-5.139054000	-5.099519000	6.357441000
6	-5.632566000	-4.296877000	4.104843000
6	-5.598652000	-6.364076000	6.004529000
6	-6.092066000	-5.565362000	3.769072000
6	-6.076266000	-6.599587000	4.711973000
1	-4.785608000	-4.924623000	7.367363000
1	-5.632791000	-3.482080000	3.372573000
1	-5.585410000	-7.165255000	6.736967000
1	-6.463977000	-5.752841000	2.765912000
1	-6.435689000	-7.587967000	4.439867000

35 -5.589375000 -2.354948000 8.475523000
8 -5.346875000 -1.499101000 2.637823000
1 -6.288925000 -1.289404000 2.752429000
1 -4.903884000 -1.681395000 3.932247000

ptTS$_{2PI,Br}$:

6	-2.725813000	0.242806000	6.645915000
6	-3.699071000	-0.670813000	6.257305000
6	-4.830159000	-0.223150000	5.557640000
6	-5.026054000	1.108050000	5.211550000
6	-4.032691000	2.013541000	5.600490000
6	-2.899172000	1.589930000	6.306515000
6	-3.822204000	-2.144036000	6.435936000
6	-5.108688000	-2.481684000	5.739598000
7	-5.648446000	-1.337544000	5.285099000
1	-1.854924000	-0.081659000	7.207480000
1	-5.904809000	1.432032000	4.663440000
1	-4.146483000	3.063512000	5.348277000
1	-2.145931000	2.316386000	6.595605000
1	-3.048959000	-2.883669000	6.036882000
6	-5.494893000	-3.800632000	5.402361000
6	-4.758370000	-4.890907000	5.965424000
6	-6.477430000	-4.064904000	4.402419000
6	-5.060608000	-6.204056000	5.543709000
6	-6.729573000	-5.352509000	3.989954000
6	-6.012228000	-6.429774000	4.569395000
1	-4.213636000	-4.749223000	6.882813000
1	-7.013183000	-3.244448000	3.933777000
1	-4.539313000	-7.037809000	6.004018000
1	-7.471586000	-5.545050000	3.221554000
1	-6.232531000	-7.446052000	4.253361000
35	-3.925851000	-2.580373000	8.409793000
8	-2.584237000	-4.378079000	5.298763000
1	-2.126588000	-4.795875000	6.049893000
1	-6.498541000	-1.280745000	4.737298000

Pro$_{2PI,Br}$:

6	-4.915720000	-0.316874000	7.555544000
6	-4.914102000	-1.723478000	7.572822000
6	-4.259773000	-2.409182000	8.634102000
6	-3.595900000	-1.727160000	9.660504000
6	-3.605804000	-0.337785000	9.617188000
6	-4.260740000	0.358387000	8.577392000
6	-5.445152000	-2.745790000	6.724936000
6	-5.134314000	-3.987025000	7.252912000
7	-4.427363000	-3.760574000	8.425462000
1	-5.410016000	0.229228000	6.756582000
1	-3.095177000	-2.266050000	10.459635000
1	-3.100738000	0.222530000	10.398913000
1	-4.248640000	1.444825000	8.578268000
1	-6.621822000	0.184685000	4.800602000
6	-5.433897000	-5.355272000	6.813217000
6	-5.684223000	-6.362514000	7.764094000
6	-5.453784000	-5.696710000	5.448843000
6	-5.948422000	-7.670672000	7.361363000
6	-5.725113000	-7.004896000	5.051011000
6	-5.971827000	-7.997170000	6.003314000
1	-5.695626000	-6.115731000	8.822060000
1	-5.244271000	-4.939008000	4.702139000
1	-6.143775000	-8.433163000	8.110138000
1	-5.732140000	-7.251418000	3.992921000
1	-6.180154000	-9.016154000	5.689746000
35	-6.512304000	-2.421184000	5.191924000
8	-6.648833000	1.156446000	4.806202000
1	-7.411058000	1.349178000	5.375019000
1	-3.957581000	-4.488377000	8.945706000

Re$_{MPI,Cl}$:

6	-3.912244000	-0.605494000	6.862063000
6	-4.336342000	-1.592977000	5.954000000
6	-4.377208000	-1.281718000	4.564465000
6	-4.016321000	-0.016056000	4.075040000
6	-3.614264000	0.945822000	5.004705000
6	-3.563486000	0.652594000	6.383699000
6	-4.778539000	-2.946268000	6.077089000
6	-5.067986000	-3.412213000	4.809848000
7	-4.821639000	-2.395052000	3.881814000
1	-3.868141000	-0.822365000	7.926321000
1	-4.005981000	0.199995000	3.010185000
1	-3.319385000	1.931117000	4.654924000
1	-3.241366000	1.423027000	7.078503000
1	-4.832917000	-3.533473000	6.983714000
6	-5.518807000	-4.763515000	4.439568000
6	-6.485473000	-5.410038000	5.231442000
6	-4.971517000	-5.461114000	3.346698000
6	-6.892594000	-6.710952000	4.939373000
6	-5.386746000	-6.759909000	3.052075000
6	-6.348224000	-7.389842000	3.845855000
1	-6.923881000	-4.878282000	6.070820000
1	-4.198883000	-4.996167000	2.741580000
1	-7.642743000	-7.191592000	5.561376000
1	-4.949405000	-7.283519000	2.206438000
1	-6.669451000	-8.401717000	3.615288000
6	-5.210502000	-2.358338000	2.477176000
1	-4.333765000	-2.359054000	1.820599000
1	-5.825223000	-3.227037000	2.245639000
1	-5.797355000	-1.453926000	2.286695000
17	-8.019331000	0.568081000	4.849571000
8	-6.904036000	1.033088000	3.615643000
1	-6.027163000	0.871308000	4.034153000

TS$_{MPI,Cl}$:

6	-3.683882000	-0.444720000	6.714126000
6	-4.247947000	-1.387276000	5.836281000
6	-4.062859000	-1.249568000	4.440554000
6	-3.340127000	-0.206647000	3.886583000
6	-2.787610000	0.725680000	4.780800000
6	-2.951024000	0.607881000	6.171681000
6	-5.067942000	-2.526353000	6.016134000
6	-5.265950000	-3.131664000	4.733660000
7	-4.698462000	-2.335935000	3.801506000
1	-3.821981000	-0.537172000	7.786862000
1	-3.199913000	-0.101957000	2.816810000
1	-2.213878000	1.556560000	4.381814000
1	-2.502166000	1.347943000	6.826109000
1	-5.363526000	-2.976280000	6.951653000
6	-5.888942000	-4.430181000	4.486602000
6	-6.960062000	-4.850800000	5.300274000
6	-5.391096000	-5.310982000	3.504550000
6	-7.532846000	-6.105731000	5.114665000
6	-5.965147000	-6.567633000	3.330857000
6	-7.040430000	-6.966914000	4.129614000
1	-7.357915000	-4.173587000	6.048434000
1	-4.530266000	-5.030901000	2.906564000
1	-8.366543000	-6.412426000	5.739359000
1	-5.564819000	-7.239939000	2.577967000

1	-7.486282000	-7.947507000	3.990858000
6	-4.899109000	-2.336405000	2.359800000
1	-3.943423000	-2.445731000	1.839772000
1	-5.565593000	-3.148834000	2.079363000
1	-5.372134000	-1.373535000	2.123274000
17	-7.561074000	-1.357337000	5.891563000
8	-6.516070000	-0.238183000	3.687719000
1	-5.924193000	0.344209000	4.195508000

$Int_{MPI,Cl}$:

6	-3.211585000	-1.182496000	7.371901000
6	-3.941842000	-1.894034000	6.402381000
6	-4.393652000	-1.227365000	5.232805000
6	-4.143642000	0.116100000	5.001675000
6	-3.411323000	0.804538000	5.982258000
6	-2.951108000	0.166969000	7.146476000
6	-4.376133000	-3.235251000	6.294536000
6	-5.033550000	-3.377055000	5.025803000
7	-5.058487000	-2.167714000	4.418973000
1	-2.862044000	-1.678254000	8.271934000
1	-4.486681000	0.621897000	4.106197000
1	-3.194930000	1.857344000	5.830372000
1	-2.385963000	0.736133000	7.877448000
1	-4.166355000	-4.054090000	6.965471000
6	-5.494161000	-4.632074000	4.441146000
6	-6.063282000	-5.614372000	5.277421000
6	-5.310241000	-4.918035000	3.072188000
6	-6.459760000	-6.838653000	4.749053000
6	-5.701413000	-6.149805000	2.554874000
6	-6.281906000	-7.109854000	3.388536000
1	-6.227873000	-5.385570000	6.325024000
1	-4.821376000	-4.197884000	2.425017000
1	-6.912236000	-7.582350000	5.398058000
1	-5.543919000	-6.363636000	1.502174000
1	-6.589405000	-8.068240000	2.980510000
6	-5.803215000	-1.771811000	3.224711000
1	-5.118009000	-1.524393000	2.409321000
1	-6.456515000	-2.586279000	2.918369000
1	-6.412141000	-0.903557000	3.483690000
17	-7.019666000	-3.048788000	7.714144000
8	-7.059409000	-0.402195000	5.959621000
1	-7.058990000	-1.223657000	6.526796000

$ptTS_{MPI,Cl}$:

6	-2.661796000	0.178817000	6.522802000
6	-3.676862000	-0.698529000	6.161853000
6	-4.781532000	-0.230459000	5.439891000
6	-4.905182000	1.095024000	5.038568000
6	-3.871026000	1.967111000	5.399724000
6	-2.765176000	1.519420000	6.130994000
6	-3.875257000	-2.152490000	6.411288000
6	-5.151488000	-2.466306000	5.662625000
7	-5.669630000	-1.312294000	5.195467000
1	-1.808362000	-0.166907000	7.098261000
1	-5.751724000	1.448696000	4.460510000
1	-3.931431000	3.008863000	5.099692000
1	-1.977020000	2.218032000	6.394423000
1	-3.108296000	-2.933361000	6.084709000
6	-5.520367000	-3.803431000	5.345653000
6	-4.883536000	-4.863346000	6.069744000
6	-6.333511000	-4.146894000	4.220748000
6	-5.162593000	-6.202138000	5.727487000
6	-6.544978000	-5.462927000	3.875363000
6	-5.967016000	-6.499825000	4.645627000
1	-4.431690000	-4.660520000	7.024544000
1	-6.748562000	-3.373569000	3.588514000
1	-4.728753000	-6.998100000	6.325030000
1	-7.150875000	-5.703482000	3.007357000
1	-6.166346000	-7.535754000	4.384324000
17	-4.071108000	-2.452121000	8.243306000
8	-2.616291000	-4.487316000	5.555082000
1	-2.253381000	-4.819973000	6.395189000
6	-6.966889000	-1.100470000	4.557562000
1	-6.876837000	-1.089111000	3.467110000
1	-7.656711000	-1.883938000	4.867725000
1	-7.357564000	-0.137733000	4.892339000

$Pro_{MPI,Cl}$:

6	-3.337855000	-1.108164000	7.977513000
6	-3.703956000	-1.969079000	6.928378000
6	-3.841494000	-1.454796000	5.608642000
6	-3.627091000	-0.098259000	5.325735000
6	-3.269895000	0.733633000	6.381276000
6	-3.124710000	0.234520000	7.694287000
6	-4.001617000	-3.359738000	6.826200000
6	-4.293115000	-3.668726000	5.508992000
7	-4.199137000	-2.490679000	4.768283000
1	-3.226366000	-1.491245000	8.987998000
1	-3.725372000	0.292426000	4.317681000
1	-3.094373000	1.788486000	6.189601000
1	-2.841340000	0.913933000	8.493342000
1	-1.004240000	-3.346822000	3.251562000
6	-4.615982000	-4.974297000	4.906030000
6	-5.614117000	-5.786323000	5.471985000
6	-3.919822000	-5.443398000	3.776725000
6	-5.907948000	-7.035047000	4.924701000
6	-4.221142000	-6.690842000	3.229840000
6	-5.213957000	-7.490770000	3.801124000
1	-6.166178000	-5.429590000	6.335688000
1	-3.133192000	-4.836370000	3.336673000
1	-6.684860000	-7.648601000	5.372391000
1	-3.673406000	-7.041109000	2.359232000
1	-5.445201000	-8.462455000	3.373608000
6	-4.518243000	-2.321942000	3.354570000
1	-3.614602000	-2.365411000	2.738829000
1	-5.201585000	-3.111334000	3.040446000
1	-5.014545000	-1.358537000	3.214037000
17	-3.906318000	-4.488793000	8.155125000
8	-1.312966000	-3.337329000	2.331912000
1	-1.037614000	-4.204402000	1.994535000

$Re_{MPI,Br}$:

6	-4.095656000	-0.720788000	7.099768000
6	-4.449806000	-1.647413000	6.102614000
6	-4.372885000	-1.253798000	4.735670000
6	-3.968261000	0.035691000	4.354217000
6	-3.638305000	0.935695000	5.369593000
6	-3.701287000	0.559868000	6.727467000
6	-4.908243000	-3.001264000	6.106686000
6	-5.093337000	-3.387374000	4.794246000
7	-4.758663000	-2.322213000	3.951865000
1	-4.140052000	-1.000856000	8.149199000
1	-3.872560000	0.315103000	3.308568000
1	-3.313396000	1.938050000	5.105476000
1	-3.433056000	1.284017000	7.491617000
1	-5.039547000	-3.641757000	6.968293000

6	-5.514847000	-4.710541000	4.306352000
6	-6.558352000	-5.389192000	4.961714000
6	-4.868342000	-5.351513000	3.233090000
6	-6.944069000	-6.666154000	4.556260000
6	-5.261912000	-6.625923000	2.824296000
6	-6.300682000	-7.288171000	3.482971000
1	-7.073534000	-4.901079000	5.783943000
1	-4.037259000	-4.862106000	2.733660000
1	-7.754685000	-7.172069000	5.073579000
1	-4.747778000	-7.105517000	1.995896000
1	-6.605139000	-8.280932000	3.163658000
6	-5.061336000	-2.186740000	2.531687000
1	-4.145130000	-2.069376000	1.943453000
1	-5.589684000	-3.073486000	2.185255000
1	-5.701809000	-1.312507000	2.376625000
35	-7.989843000	-0.328114000	4.545160000
8	-6.883722000	0.833420000	3.592963000
1	-6.037737000	0.771456000	4.090519000

$TS_{MPI,Br}$:

6	-3.673094000	-0.425165000	6.701352000
6	-4.252229000	-1.354729000	5.833581000
6	-4.049565000	-1.241913000	4.444809000
6	-3.301559000	-0.218519000	3.880517000
6	-2.730380000	0.709226000	4.764049000
6	-2.904679000	0.603946000	6.151555000
6	-5.127619000	-2.482499000	6.021181000
6	-5.276897000	-3.114602000	4.740780000
7	-4.688231000	-2.333316000	3.812000000
1	-3.824608000	-0.496632000	7.773988000
1	-3.152867000	-0.132588000	2.809943000
1	-2.133576000	1.521370000	4.360354000
1	-2.439616000	1.335202000	6.805241000
1	-5.305601000	-2.994525000	6.954415000
6	-5.899334000	-4.413054000	4.482621000
6	-7.024438000	-4.812960000	5.229239000
6	-5.342154000	-5.313629000	3.552289000
6	-7.586314000	-6.071828000	5.032921000
6	-5.907751000	-6.572497000	3.365576000
6	-7.033061000	-6.954587000	4.101219000
1	-7.471715000	-4.116600000	5.930612000
1	-4.444631000	-5.043348000	3.005087000
1	-8.461037000	-6.363043000	5.607089000
1	-5.462010000	-7.259669000	2.652550000
1	-7.472015000	-7.937018000	3.953480000
6	-4.937329000	-2.302685000	2.380194000
1	-3.994900000	-2.233036000	1.831980000
1	-5.477188000	-3.195299000	2.073563000
1	-5.566909000	-1.415296000	2.228593000
35	-7.391929000	-1.325277000	6.205473000
8	-6.710785000	-0.461575000	3.730869000
1	-6.029194000	0.164220000	4.034201000

$Int_{MPI,Br}$:

6	-3.035451000	-1.119712000	7.326244000
6	-3.821064000	-1.819685000	6.399885000
6	-4.316850000	-1.149697000	5.255109000
6	-4.063284000	0.196030000	5.010478000
6	-3.276111000	0.874810000	5.948150000
6	-2.766447000	0.227132000	7.085958000
6	-4.294970000	-3.165019000	6.318819000
6	-4.996780000	-3.293505000	5.081277000
7	-5.027222000	-2.079769000	4.473707000
1	-2.650004000	-1.616872000	8.211066000
1	-4.445949000	0.702313000	4.131216000
1	-3.053171000	1.924982000	5.786584000
1	-2.156126000	0.786398000	7.788317000
1	-3.999714000	-4.000395000	6.934921000
6	-5.507079000	-4.535517000	4.494683000
6	-6.176520000	-5.472274000	5.305029000
6	-5.271352000	-4.844645000	3.140226000
6	-6.607785000	-6.681842000	4.766487000
6	-5.702059000	-6.059333000	2.611310000
6	-6.373252000	-6.979423000	3.420866000
1	-6.387683000	-5.219707000	6.338842000
1	-4.717695000	-4.152789000	2.513427000
1	-7.134737000	-7.391647000	5.397464000
1	-5.504758000	-6.290312000	1.568762000
1	-6.710123000	-7.924821000	3.005498000
6	-5.809024000	-1.680839000	3.305533000
1	-5.147517000	-1.385995000	2.486145000
1	-6.433329000	-2.512594000	2.985599000
1	-6.445995000	-0.842130000	3.595110000
35	-6.611120000	-2.948466000	7.944570000
8	-6.970403000	-0.184055000	6.038122000
1	-6.925389000	-0.986970000	6.621920000

$ptTS_{MPI,Br}$:

6	-2.651900000	0.180953000	6.486226000
6	-3.672589000	-0.698024000	6.142725000
6	-4.800253000	-0.226554000	5.457596000
6	-4.941796000	1.103322000	5.076867000
6	-3.901292000	1.975526000	5.418098000
6	-2.771992000	1.524213000	6.111628000
6	-3.855963000	-2.151811000	6.373546000
6	-5.144791000	-2.465701000	5.664672000
7	-5.685064000	-1.311346000	5.221968000
1	-1.784162000	-0.167755000	7.037997000
1	-5.807304000	1.459698000	4.529175000
1	-3.974717000	3.020595000	5.132872000
1	-1.980078000	2.223918000	6.360534000
1	-3.092847000	-2.944611000	6.075915000
6	-5.513505000	-3.802406000	5.339050000
6	-4.884688000	-4.867596000	6.060720000
6	-6.323955000	-4.134079000	4.210093000
6	-5.163890000	-6.203113000	5.706436000
6	-6.537497000	-5.447410000	3.854138000
6	-5.964717000	-6.490940000	4.618970000
1	-4.445251000	-4.667868000	7.023005000
1	-6.732330000	-3.353365000	3.581875000
1	-4.734578000	-7.004325000	6.300248000
1	-7.140253000	-5.680316000	2.981845000
1	-6.165165000	-7.524521000	4.349297000
35	-4.067778000	-2.454947000	8.379005000
8	-2.611084000	-4.507023000	5.570361000
1	-2.267797000	-4.828527000	6.422959000
6	-7.005974000	-1.103022000	4.633624000
1	-6.950513000	-1.048780000	3.542196000
1	-7.669199000	-1.912819000	4.933837000
1	-7.405299000	-0.161822000	5.016906000

$Pro_{MPI,Br}$:

6	-2.755740000	-1.005129000	7.236325000
6	-3.563695000	-1.763451000	6.373747000
6	-4.477915000	-1.111066000	5.502208000
6	-4.590363000	0.285599000	5.472598000

6	-3.777764000	1.016005000	6.333895000
6	-2.870612000	0.378519000	7.208530000
6	-3.723050000	-3.154859000	6.115496000
6	-4.689497000	-3.334964000	5.144401000
7	-5.149075000	-2.072572000	4.769720000
1	-2.057955000	-1.501640000	7.904858000
1	-5.289772000	0.783511000	4.808148000
1	-3.845661000	2.100233000	6.334817000
1	-2.253926000	0.982854000	7.867896000
1	-0.814559000	-4.104899000	3.824542000
6	-5.213643000	-4.591377000	4.579161000
6	-6.593740000	-4.861242000	4.570463000
6	-4.328916000	-5.557229000	4.067845000
6	-7.077635000	-6.064880000	4.058069000
6	-4.818206000	-6.760987000	3.560316000
6	-6.191199000	-7.018908000	3.552497000
1	-7.287015000	-4.133809000	4.982948000
1	-3.261242000	-5.358386000	4.050211000
1	-8.146468000	-6.259355000	4.061419000
1	-4.124112000	-7.496784000	3.163515000
1	-6.568294000	-7.956931000	3.154643000
6	-6.079589000	-1.761202000	3.692430000
1	-5.656085000	-0.977719000	3.057218000
1	-6.245113000	-2.651014000	3.086608000
1	-7.041069000	-1.416759000	4.088274000
35	-2.806686000	-4.522475000	7.049403000
8	-0.732150000	-4.973136000	4.249590000
1	-1.158124000	-4.835437000	5.113527000

17.2. Explicit solvent model calculations with 57 water molecules

$Re_{2PI,Cl}$:

6	-4.548506000	-0.814684000	7.212232000
6	-4.779822000	-1.753242000	6.188598000
6	-4.514927000	-1.385181000	4.835471000
6	-4.024593000	-0.119653000	4.490479000
6	-3.797540000	0.783473000	5.523032000
6	-4.057452000	0.440452000	6.871564000
6	-5.245740000	-3.100885000	6.157315000
6	-5.257825000	-3.512011000	4.834122000
7	-4.813030000	-2.464073000	4.038634000
1	-4.740702000	-1.072449000	8.250670000
1	-3.823938000	0.142585000	3.457473000
1	-3.389648000	1.763613000	5.300605000
1	-3.848686000	1.172686000	7.646251000
1	-5.530375000	-3.696280000	7.013166000
6	-5.640394000	-4.815834000	4.288258000
6	-6.231623000	-5.785738000	5.127934000
6	-5.422764000	-5.159257000	2.939908000
6	-6.596831000	-7.036776000	4.637099000
6	-5.777963000	-6.423072000	2.452098000
6	-6.371984000	-7.366274000	3.295232000
1	-6.401602000	-5.556302000	6.175276000
1	-4.964265000	-4.449537000	2.258462000
1	-7.044383000	-7.764485000	5.308764000
1	-5.544204000	-6.677505000	1.420074000
1	-6.645362000	-8.345902000	2.917352000
17	-8.623744000	-1.222034000	1.417468000
8	-7.673632000	0.238656000	1.183471000
1	-7.280941000	0.522079000	2.052052000
1	-4.720469000	-2.446990000	3.017696000
8	-10.599567000	-0.656599000	-1.708386000
1	-9.994813000	0.069673000	-1.438180000
1	-10.051073000	-1.476973000	-1.703175000
8	-8.934288000	1.554852000	-0.915887000
1	-8.049548000	1.696707000	-1.329070000
1	-8.685639000	1.281677000	-0.006990000
8	-12.106196000	-1.941861000	0.136584000
1	-11.632646000	-1.327031000	-0.489509000
1	-12.408452000	-1.387651000	0.895084000
8	-5.668825000	-0.522976000	-0.528581000
1	-6.392058000	-0.318816000	0.113377000
1	-6.071543000	-1.168496000	-1.169258000
8	-8.482151000	-7.054613000	0.437696000
1	-9.437754000	-6.802458000	0.557342000
1	-7.997866000	-6.507059000	1.078363000
8	-9.347077000	-3.141379000	-1.487036000
1	-8.437032000	-2.928156000	-1.809775000
1	-9.276975000	-3.250984000	-0.513607000
8	-9.949011000	-3.171210000	1.220452000
1	-10.779727000	-2.722797000	0.901515000
1	-10.229276000	-3.701132000	2.012685000
8	-11.774605000	-4.494677000	-1.085649000
1	-12.110212000	-3.682903000	-0.646052000
1	-10.926453000	-4.191923000	-1.476414000
8	-11.132605000	-4.693257000	3.110404000
1	-10.522303000	-5.026172000	3.816420000
1	-11.240690000	-5.395061000	2.424914000
8	-11.080721000	-6.255974000	0.828764000
1	-11.695614000	-6.998896000	0.715235000
1	-11.338464000	-5.590137000	0.119376000
8	-6.195203000	1.975839000	-1.435700000
1	-5.840705000	2.219675000	-2.306042000
1	-5.862934000	1.054303000	-1.254735000
8	-4.196736000	-2.103523000	1.220399000
1	-4.686939000	-1.482438000	0.624842000
1	-3.269289000	-1.743462000	1.284276000
8	-5.669779000	-4.591497000	-2.456998000
1	-5.067625000	-4.559334000	-1.669584000
1	-6.447845000	-5.161540000	-2.202354000
8	-6.860704000	-2.257932000	-2.288855000
1	-6.935980000	-1.845386000	-3.164734000
1	-6.334370000	-3.108878000	-2.430012000
8	-7.623329000	-6.432365000	-2.174588000
1	-7.866617000	-6.680235000	-1.254424000
1	-6.898710000	-7.044640000	-2.447495000
1	-12.284629000	4.521572000	1.728365000
1	-13.496304000	3.564820000	1.613527000
1	-13.510157000	-0.219952000	2.603926000
1	-12.056881000	0.354060000	2.508460000
1	-10.793558000	4.971942000	0.050771000
1	-11.644989000	6.250171000	0.367954000
1	-9.274497000	5.738139000	1.383708000
1	-8.611961000	4.427103000	1.889079000
1	-12.238641000	-2.297448000	5.929326000
1	-13.323170000	-2.544440000	7.032265000
1	-10.380572000	2.098376000	2.705361000
1	-11.881737000	2.593205000	2.707849000
1	-12.053618000	-1.604393000	3.679679000
1	-11.723126000	-3.138696000	3.854871000
1	-9.526989000	3.278313000	-0.929403000
1	-8.929693000	4.573739000	-0.336284000
1	-10.970314000	1.368204000	4.631125000

1	-9.924652000	1.864299000	5.656551000
1	-7.959950000	2.531214000	2.720117000
1	-8.825691000	3.253127000	3.787888000
1	-12.044369000	-0.602280000	7.483081000
1	-11.446302000	0.686601000	6.844291000
1	-9.080421000	4.125539000	5.993600000
1	-7.886805000	3.121598000	5.985516000
8	-12.387323000	-2.315050000	6.916173000
8	-12.592772000	-0.487823000	2.433193000
8	-11.705605000	-2.272837000	4.323070000
8	-11.804310000	0.328531000	7.702087000
8	-8.766230000	3.308898000	5.573724000
8	-10.525924000	1.111225000	5.472293000
8	-12.867276000	3.920303000	2.261104000
8	-11.302509000	1.806829000	2.912130000
8	-11.185531000	5.510843000	0.797197000
8	-9.610290000	4.260599000	-0.974081000
8	-8.431710000	5.240514000	1.340567000
8	-8.821546000	3.015802000	2.827717000
1	-5.869995000	2.162981000	2.016599000
1	-6.012411000	2.269369000	3.560047000
1	-5.703046000	3.848802000	5.286872000
1	-4.478587000	3.982272000	4.273516000
1	-5.300470000	3.940004000	1.107058000
1	-5.397631000	2.816673000	-0.036125000
1	-6.025924000	1.908790000	6.663651000
1	-6.921492000	2.601667000	7.762059000
1	-5.898739000	4.880832000	2.969458000
1	-6.772556000	5.534939000	1.835199000
8	-5.455400000	3.783629000	4.347506000
8	-6.380811000	2.779577000	6.926506000
8	-6.384229000	1.785128000	2.783196000
8	-4.999597000	3.032534000	0.842281000
8	-5.837741000	5.335227000	2.094158000
1	-4.804371000	3.527406000	7.605039000
1	-3.804844000	3.509992000	8.785870000
8	-3.937703000	3.884764000	7.901601000
1	-9.205335000	-4.010210000	5.126922000
1	-8.696175000	-5.501965000	5.166362000
1	-8.869570000	-2.169290000	5.987709000
1	-9.915420000	-2.130698000	4.795088000
1	-10.199827000	-4.631784000	6.960642000
1	-11.249004000	-3.628360000	7.508186000
1	-10.407166000	0.232633000	8.518040000
1	-9.690733000	-0.093858000	9.883203000
1	-9.279426000	-2.926704000	7.882414000
1	-8.936382000	-1.411979000	8.152526000
1	-8.488323000	1.563957000	8.967698000
1	-7.355421000	2.186487000	9.827720000
8	-10.512231000	-4.215778000	7.795493000
8	-9.505353000	-4.968559000	5.246148000
8	-8.991436000	-2.382436000	5.021422000
8	-8.611521000	-2.219989000	7.694023000
8	-9.498137000	0.126145000	8.957224000
8	-7.908628000	2.367090000	9.051474000
1	-2.650482000	3.973611000	3.020781000
1	-3.074369000	5.424919000	3.493020000
1	-1.113455000	-2.085145000	1.208738000
1	-1.325785000	-0.559606000	0.859410000
1	0.453065000	-3.700364000	0.419705000
1	-0.815323000	-3.446105000	-0.474734000
1	-1.203741000	1.730646000	0.391849000
1	-0.222893000	1.070849000	1.312617000
1	-4.535313000	6.493617000	2.433316000
1	-3.165315000	7.023394000	1.947532000
1	-3.283085000	2.771958000	1.351631000
1	-2.125031000	2.109321000	2.240558000
1	0.518450000	1.728682000	5.632442000
1	-0.918891000	2.254719000	5.893748000
1	-1.271011000	0.006765000	2.920255000
1	-0.821765000	1.062217000	4.037465000
1	-2.072327000	4.074758000	5.336842000
1	-2.491054000	3.713507000	6.795558000
8	-1.626512000	-1.281014000	1.465954000
8	-0.517137000	1.032742000	0.378182000
8	-0.515228000	-3.642277000	0.461506000
8	-1.045189000	0.946085000	3.073436000
8	-2.354809000	2.883210000	1.668310000
8	-3.687099000	6.889957000	2.754860000
8	-2.917368000	4.499112000	3.822035000
8	-1.715435000	3.730373000	6.188681000
8	-0.404050000	1.429827000	5.664454000
8	-4.844246000	-9.772880000	0.721703000
1	-4.445347000	-9.728535000	1.623666000
1	-5.819905000	-9.716238000	0.858116000
8	-1.697536000	-3.192014000	-1.891733000
1	-2.558465000	-3.514000000	-1.508525000
1	-1.443403000	-3.881103000	-2.526369000
8	-5.180147000	-7.383903000	-3.080219000
1	-5.105619000	-6.417072000	-3.206176000
1	-4.756576000	-7.503839000	-2.190506000
8	-4.271945000	-7.460112000	-0.526355000
1	-4.410483000	-8.328326000	-0.053971000
1	-3.387301000	-7.133368000	-0.274863000
8	-2.100359000	-5.949319000	0.612558000
1	-1.391104000	-5.263804000	0.610827000
1	-2.220373000	-6.215013000	1.551585000
8	-7.619729000	-9.624496000	1.075956000
1	-7.983194000	-8.746028000	0.808404000
1	-8.008160000	-10.255425000	0.450038000
8	-3.551036000	-9.590028000	3.182885000
1	-3.200978000	-8.671314000	3.204431000
1	-2.762495000	-10.141210000	3.056403000
8	-2.636772000	-6.908596000	3.192645000
1	-1.976187000	-6.715949000	3.879381000
1	-3.426136000	-6.394385000	3.445466000
8	-3.829817000	-4.155766000	-0.509951000
1	-4.000648000	-3.431512000	0.153076000
1	-3.340384000	-4.868256000	-0.023352000

TS$_{2P1,CI}$:

6	-5.602787000	-0.274158000	6.113922000
6	-5.532949000	-1.309314000	5.157244000
6	-4.476319000	-1.314371000	4.202004000
6	-3.529689000	-0.301294000	4.137492000
6	-3.627919000	0.716814000	5.091483000
6	-4.633390000	0.718106000	6.080001000
6	-6.316466000	-2.452296000	4.902382000
6	-5.661644000	-3.185087000	3.849941000
7	-4.585641000	-2.472421000	3.443879000
1	-6.394273000	-0.255861000	6.856878000
1	-2.739941000	-0.294218000	3.396285000
1	-2.910827000	1.530646000	5.072153000
1	-4.649756000	1.520642000	6.808220000
1	-7.186079000	-2.792571000	5.450869000
6	-5.975093000	-4.525070000	3.390328000

6	-7.024336000	-5.235098000	4.010650000
6	-5.236987000	-5.154044000	2.363196000
6	-7.297921000	-6.549795000	3.641039000
6	-5.521435000	-6.465947000	1.996179000
6	-6.540811000	-7.175477000	2.648197000
1	-7.621962000	-4.762623000	4.782355000
1	-4.454230000	-4.615609000	1.838788000
1	-8.102559000	-7.086207000	4.135312000
1	-4.954417000	-6.948799000	1.205924000
1	-6.745623000	-8.204256000	2.371979000
17	-8.094201000	-2.124142000	2.742492000
8	-6.623834000	-0.138232000	2.209945000
1	-6.191918000	0.272467000	2.976072000
1	-4.030268000	-2.626088000	2.578764000
8	-10.159685000	-0.627455000	-0.814301000
1	-9.488127000	-0.132001000	-0.282134000
1	-9.770531000	-1.521737000	-0.962458000
8	-8.320251000	0.963044000	0.514140000
1	-7.625972000	1.115197000	-0.165971000
1	-7.790322000	0.602586000	1.294574000
8	-11.908593000	-1.836694000	0.799690000
1	-11.333347000	-1.249126000	0.228427000
1	-12.150417000	-1.298552000	1.590355000
8	-5.238083000	-0.816414000	0.155222000
1	-5.741805000	-0.605550000	1.031893000
1	-5.827002000	-1.412427000	-0.371742000
8	-8.753136000	-6.740681000	0.303377000
1	-9.726938000	-6.603455000	0.453793000
1	-8.322807000	-6.269899000	1.035519000
8	-9.293383000	-3.287615000	-1.031444000
1	-8.362836000	-3.071794000	-1.284277000
1	-9.273479000	-3.524735000	-0.076785000
8	-9.941268000	-3.522274000	1.602068000
1	-10.667530000	-2.864640000	1.425111000
1	-10.266638000	-4.076764000	2.350130000
8	-11.967789000	-4.168673000	-0.779617000
1	-12.162725000	-3.389878000	-0.211439000
1	-11.084377000	-3.954218000	-1.147367000
8	-11.439427000	-4.967718000	3.378408000
1	-11.169553000	-5.509964000	4.156286000
1	-11.604899000	-5.564012000	2.611689000
8	-11.395608000	-6.210499000	0.880378000
1	-11.994569000	-6.932568000	0.629634000
1	-11.635753000	-5.445415000	0.273403000
8	-5.979398000	1.486925000	-0.894783000
1	-5.695462000	1.687504000	-1.800023000
1	-5.547197000	0.621376000	-0.631476000
8	-3.324910000	-2.447068000	1.009379000
1	-3.961282000	-1.768516000	0.636476000
1	-2.436081000	-2.000994000	1.092745000
8	-5.473497000	-4.437670000	-2.466922000
1	-4.760284000	-4.526058000	-1.785325000
1	-6.210664000	-5.053749000	-2.202850000
8	-6.781979000	-2.303527000	-1.607537000
1	-6.927845000	-1.669136000	-2.328384000
1	-6.222187000	-3.039738000	-2.005648000
8	-7.395121000	-6.311768000	-2.149530000
1	-7.820596000	-6.455011000	-1.277146000
1	-6.663096000	-6.965219000	-2.237963000
1	-12.328735000	4.215001000	1.408177000
1	-13.370084000	3.079532000	1.262707000
1	-13.131853000	-0.342428000	3.464328000
1	-11.813894000	0.460886000	3.219050000
1	-10.454875000	4.363907000	0.174481000
1	-11.431140000	5.513518000	-0.258220000
1	-9.411947000	5.807966000	1.318206000
1	-8.773435000	4.934351000	2.432919000
1	-12.406093000	-2.298632000	6.044847000
1	-13.841725000	-2.130440000	6.652192000
1	-10.502863000	2.473196000	3.362896000
1	-12.003439000	2.658986000	2.915204000
1	-11.555237000	-1.748133000	4.152884000
1	-11.616523000	-3.323977000	4.181745000
1	-8.891636000	2.639589000	0.260339000
1	-8.522459000	4.145997000	0.434000000
1	-11.729216000	2.260459000	5.223871000
1	-10.976119000	2.968675000	6.364819000
1	-8.215948000	3.180855000	3.524652000
1	-9.286887000	3.907488000	4.403703000
1	-12.366865000	-0.464954000	7.466320000
1	-11.905518000	1.040668000	7.216248000
1	-9.459094000	4.952515000	6.508371000
1	-8.549982000	3.678058000	6.412902000
8	-12.902314000	-2.128349000	6.896433000
8	-12.215802000	-0.455325000	3.163161000
8	-11.396350000	-2.537357000	4.730973000
8	-12.007725000	0.336435000	7.911077000
8	-9.441800000	4.044254000	6.167480000
8	-11.785032000	2.441771000	6.192276000
8	-12.976317000	3.669298000	1.924968000
8	-11.405040000	2.071708000	3.455767000
8	-11.075147000	5.067325000	0.526998000
8	-9.012765000	3.530040000	-0.151598000
8	-8.544514000	5.583006000	1.714428000
8	-9.122468000	3.596955000	3.477646000
1	-6.262272000	2.399946000	2.841075000
1	-6.040788000	3.447527000	3.936167000
1	-4.936243000	4.911206000	5.133288000
1	-4.131509000	4.678530000	3.799486000
1	-5.399599000	3.800252000	1.322203000
1	-5.514176000	2.459907000	0.485323000
1	-6.887480000	2.433237000	5.702036000
1	-7.197863000	2.193952000	7.248436000
1	-5.707115000	5.495470000	2.651170000
1	-6.753149000	5.735450000	1.519940000
8	-5.048713000	4.836539000	4.155842000
8	-7.009800000	2.891739000	6.560484000
8	-6.602194000	2.650671000	3.733566000
8	-5.212813000	2.829201000	1.352208000
8	-5.795061000	5.557725000	1.670556000
1	-5.466918000	3.747180000	6.820984000
1	-4.644777000	4.880269000	7.519460000
8	-4.619220000	4.261399000	6.772306000
1	-10.056821000	-5.061594000	5.979531000
1	-10.325239000	-6.598528000	6.176996000
1	-9.243148000	-3.407537000	7.019966000
1	-9.784802000	-3.070101000	5.565191000
1	-11.677597000	-4.984922000	7.424046000
1	-12.420560000	-3.663548000	7.760156000
1	-10.592544000	-0.102916000	8.516640000
1	-9.894035000	-0.307668000	9.906579000
1	-10.458549000	-3.616413000	8.626388000
1	-9.687783000	-2.242075000	8.769320000
1	-8.374343000	0.623439000	8.644941000
1	-6.873604000	0.776618000	9.045984000
8	-11.977855000	-4.436377000	8.181982000

8	-10.763540000	-5.771842000	5.918832000
8	-9.151770000	-3.647624000	6.049678000
8	-9.553534000	-3.211439000	8.652027000
8	-9.734683000	-0.428052000	8.955944000
8	-7.593690000	1.235200000	8.587888000
1	-2.448085000	3.993969000	2.462955000
1	-2.689747000	5.551101000	2.570173000
1	-0.368304000	-2.149469000	0.997978000
1	-0.741490000	-0.640376000	0.637995000
1	0.972015000	-4.015699000	0.309507000
1	-0.243559000	-3.694514000	-0.628480000
1	-1.298050000	1.557140000	0.115636000
1	-0.054889000	1.268965000	0.902402000
1	-4.270841000	6.491553000	1.406894000
1	-2.958475000	6.577717000	0.597249000
1	-3.450341000	2.523114000	1.401311000
1	-2.090209000	1.951303000	2.034262000
1	0.664754000	2.153878000	5.422221000
1	-0.833742000	2.536428000	5.549962000
1	-0.631981000	0.068613000	2.677600000
1	-0.471015000	1.239464000	3.754018000
1	-2.043519000	4.291455000	4.801854000
1	-2.763435000	3.879892000	6.121087000
8	-0.876771000	-1.360465000	1.304190000
8	-0.453301000	1.066431000	0.032500000
8	0.017547000	-3.837571000	0.328462000
8	-0.688626000	1.036254000	2.805077000
8	-2.462237000	2.607360000	1.399153000
8	-3.330719000	6.788344000	1.468429000
8	-2.557210000	4.733643000	3.118498000
8	-1.882260000	3.917551000	5.698082000
8	-0.224514000	1.767163000	5.392090000
8	-4.713437000	-9.463679000	0.389146000
1	-4.488043000	-9.492977000	1.348725000
1	-5.682610000	-9.302110000	0.329057000
8	-1.020543000	-3.541895000	-2.127593000
1	-1.915832000	-3.837549000	-1.809374000
1	-0.712116000	-4.269419000	-2.691360000
8	-4.955825000	-7.306090000	-2.950175000
1	-4.863789000	-6.350872000	-3.122462000
1	-4.229784000	-7.507696000	-2.303233000
8	-3.002515000	-8.062088000	-1.190834000
1	-3.588307000	-8.542687000	-0.551011000
1	-2.485125000	-7.425230000	-0.659274000
8	-1.663384000	-6.121618000	0.480021000
1	-0.959018000	-5.432026000	0.521617000
1	-1.880664000	-6.361131000	1.408430000
8	-7.511932000	-9.241417000	0.197294000
1	-8.005087000	-8.388501000	0.240852000
1	-7.738409000	-9.610886000	-0.670301000
8	-3.845450000	-9.482351000	3.029436000
1	-3.304925000	-8.661847000	3.058866000
1	-3.192400000	-10.199990000	3.043691000
8	-2.452812000	-7.008074000	3.036859000
1	-1.752329000	-6.961910000	3.709594000
1	-3.146357000	-6.403006000	3.354081000
8	-3.276960000	-4.383455000	-0.854657000
1	-3.316639000	-3.649572000	-0.180276000
1	-2.846278000	-5.130373000	-0.365906000

Int$_{2PI,Cl}$:

6	-3.572813000	-0.691504000	7.591032000
6	-3.761287000	-2.065161000	7.641309000
6	-3.771585000	-2.720198000	8.874428000
6	-3.624845000	-2.070762000	10.091303000
6	-3.443262000	-0.684743000	10.033710000
6	-3.410285000	-0.009383000	8.806872000
6	-4.000437000	-3.093195000	6.568993000
6	-4.046843000	-4.403511000	7.349866000
7	-3.949466000	-4.104245000	8.629764000
1	-3.516547000	-0.155110000	6.648224000
1	-3.645443000	-2.605578000	11.034542000
1	-3.317569000	-0.128455000	10.956945000
1	-3.248377000	1.063992000	8.792606000
1	-4.973778000	-2.958110000	6.067030000
6	-4.212103000	-5.734606000	6.823155000
6	-4.000386000	-6.870627000	7.638603000
6	-4.588143000	-5.915347000	5.472343000
6	-4.170018000	-8.145301000	7.118460000
6	-4.752405000	-7.197532000	4.958211000
6	-4.545462000	-8.310727000	5.780134000
1	-3.675309000	-6.765122000	8.668568000
1	-4.764432000	-5.057551000	4.834626000
1	-3.999613000	-9.010981000	7.749810000
1	-5.019954000	-7.336513000	3.914927000
1	-4.668915000	-9.310367000	5.374611000
17	-2.724203000	-3.100586000	5.278034000
8	-7.815510000	0.025386000	2.702038000
1	-7.378368000	0.742993000	3.194266000
1	-4.013264000	-4.785998000	9.377925000
8	-10.120390000	-0.226161000	-1.147972000
1	-9.318150000	0.149183000	-0.693188000
1	-9.760336000	-0.552463000	-2.025820000
8	-7.996216000	0.838523000	0.112485000
1	-7.116055000	0.498284000	-0.191343000
1	-8.021743000	0.629960000	1.087264000
8	-10.238304000	-2.367028000	0.451756000
1	-10.235448000	-1.621977000	-0.219155000
1	-10.462109000	-1.910850000	1.305130000
8	-5.963026000	-1.644219000	1.638821000
1	-7.087646000	-0.614715000	2.474059000
1	-6.621187000	-2.307130000	1.306851000
8	-8.261308000	-5.589731000	-0.817019000
1	-9.040878000	-5.948654000	-0.327438000
1	-7.919475000	-4.883304000	-0.206490000
8	-8.895557000	-1.236911000	-3.306686000
1	-7.951737000	-0.952047000	-3.157611000
1	-8.856834000	-2.203955000	-3.142642000
8	-7.810725000	-3.536102000	0.889271000
1	-8.660177000	-3.055622000	0.681499000
1	-8.026188000	-4.028133000	1.721622000
8	-12.035266000	-4.345325000	0.089115000
1	-11.425925000	-3.559482000	0.177759000
1	-12.119839000	-4.478328000	-0.868519000
8	-8.921959000	-4.923415000	2.941419000
1	-8.609184000	-5.450859000	3.716653000
1	-9.522840000	-5.480962000	2.388923000
8	-10.312186000	-6.185101000	0.942501000
1	-10.730213000	-7.057379000	1.018386000
1	-11.033139000	-5.554187000	0.638890000
8	-5.518610000	-0.058800000	-0.334887000
1	-4.569274000	-0.140562000	-0.554838000
1	-5.752996000	-1.032008000	0.821470000
8	-3.831232000	-3.212297000	1.443396000
1	-4.627687000	-2.640706000	1.660582000
1	-3.028620000	-2.647209000	1.604111000

8	-5.585308000	-3.430860000	-3.192942000
1	-5.042061000	-3.521679000	-2.371401000
1	-6.517344000	-3.667627000	-2.964801000
8	-6.250112000	-0.674510000	-2.906487000
1	-6.032294000	-0.475319000	-1.951811000
1	-5.915475000	-1.586977000	-3.057114000
8	-8.299985000	-3.990516000	-2.947561000
1	-8.456668000	-4.603140000	-2.174469000
1	-8.472411000	-4.526726000	-3.737826000
1	-11.358603000	2.698864000	-0.467001000
1	-11.237301000	1.146848000	-0.643998000
1	-11.645689000	-1.156876000	3.095724000
1	-10.643827000	-0.044931000	2.617475000
1	-9.597579000	3.807719000	-1.168729000
1	-10.794198000	4.675157000	-1.696238000
1	-9.233184000	5.279562000	0.261268000
1	-8.611868000	4.719089000	1.572158000
1	-10.506945000	-2.841809000	5.394187000
1	-12.012855000	-3.255636000	5.525432000
1	-10.025912000	2.148828000	2.406589000
1	-11.225975000	1.707999000	1.458228000
1	-9.564029000	-1.883821000	3.780978000
1	-9.259210000	-3.428711000	3.757894000
1	-8.015593000	2.415176000	-0.607098000
1	-7.808543000	3.937794000	-0.406184000
1	-11.383177000	1.675275000	4.148714000
1	-10.655376000	2.333321000	5.340522000
1	-7.881142000	2.996337000	2.777493000
1	-8.998440000	3.608398000	3.658274000
1	-11.456455000	-1.189070000	6.527775000
1	-11.362926000	0.362436000	6.181892000
1	-9.316739000	4.292856000	5.915310000
1	-8.304039000	3.107951000	5.734725000
8	-11.258993000	-2.941735000	6.050729000
8	-10.739317000	-1.033513000	2.771677000
8	-9.232982000	-2.628221000	4.343841000
8	-11.385607000	-0.292311000	6.927818000
8	-9.188263000	3.459776000	5.435086000
8	-11.431317000	1.770573000	5.125432000
8	-11.741315000	1.843913000	-0.161544000
8	-10.835148000	1.579345000	2.371164000
8	-10.453262000	4.248437000	-0.893707000
8	-7.979711000	3.272372000	-1.103645000
8	-8.429659000	5.340683000	0.820580000
8	-8.790310000	3.392943000	2.714812000
1	-5.817377000	2.082483000	2.437821000
1	-5.856855000	3.282019000	3.407920000
1	-5.037588000	4.917349000	4.459840000
1	-4.080852000	4.567475000	3.246045000
1	-5.299541000	2.915692000	0.608606000
1	-5.258878000	1.279525000	0.404337000
1	-6.473769000	2.228019000	5.064790000
1	-6.692469000	1.688989000	6.533292000
1	-5.619210000	4.981309000	1.815516000
1	-6.574091000	5.299481000	0.642460000
8	-5.033109000	4.735293000	3.488710000
8	-6.696176000	2.523659000	5.977703000
8	-6.309710000	2.403466000	3.250676000
8	-4.978857000	2.077969000	1.002407000
8	-5.670074000	4.962323000	0.832473000
1	-5.476401000	3.740058000	6.253138000
1	-5.067377000	5.132445000	6.858769000
8	-4.824504000	4.488980000	6.174852000
1	-7.611737000	-4.908584000	5.576393000
1	-7.538651000	-6.480693000	5.592449000
1	-7.346024000	-3.182505000	6.744086000
1	-7.645277000	-2.873444000	5.232580000
1	-9.403194000	-5.377384000	6.775731000
1	-10.523829000	-4.330179000	7.005084000
1	-9.985514000	-0.398109000	7.725172000
1	-9.334934000	-0.207661000	9.152346000
1	-8.777303000	-3.796805000	8.104392000
1	-8.523480000	-2.258692000	8.306365000
1	-7.627335000	0.004049000	7.650885000
1	-6.147149000	0.235465000	8.081616000
8	-9.950253000	-4.971714000	7.484815000
8	-8.144926000	-5.743776000	5.414675000
8	-7.003571000	-3.346654000	5.814098000
8	-8.062028000	-3.128422000	8.268120000
8	-9.141035000	-0.549965000	8.265672000
8	-6.721610000	0.212276000	7.300534000
1	-2.325340000	3.759932000	2.064219000
1	-2.610645000	5.299814000	2.013036000
1	-0.977694000	-2.186844000	1.053034000
1	-2.000256000	-1.029859000	0.820260000
1	0.724382000	-3.297570000	-0.352093000
1	-0.580611000	-2.766513000	-1.043010000
1	-2.322078000	0.878207000	-0.204438000
1	-2.155715000	-0.372072000	-1.208688000
1	-4.168119000	5.939461000	0.623068000
1	-2.799674000	6.059738000	-0.085802000
1	-3.241624000	2.165009000	1.118435000
1	-1.829531000	1.714685000	1.730115000
1	-1.466949000	0.964123000	5.886090000
1	-2.180567000	2.213054000	5.303895000
1	-1.096283000	-0.161678000	2.524840000
1	-1.404531000	0.853765000	3.688786000
1	-2.158966000	4.236113000	4.468442000
1	-3.042781000	4.142939000	5.749145000
8	-1.633604000	-1.626013000	1.530949000
8	-2.635845000	-0.029546000	-0.423015000
8	-0.242006000	-3.281130000	-0.262369000
8	-0.936631000	0.761828000	2.821965000
8	-2.259821000	2.303283000	1.043336000
8	-3.265445000	6.333303000	0.720406000
8	-2.469640000	4.549900000	2.648980000
8	-2.155440000	3.911949000	5.395905000
8	-2.159903000	1.213936000	5.254451000
8	-4.719206000	-8.717225000	-1.149391000
1	-4.701140000	-8.445690000	-0.204065000
1	-5.534899000	-8.289552000	-1.524444000
8	-1.669134000	-1.912307000	-2.104496000
1	-2.481737000	-2.458047000	-1.889016000
1	-1.529571000	-2.001200000	-3.060686000
8	-5.049183000	-6.099413000	-3.994734000
1	-5.144499000	-5.140961000	-3.796135000
1	-4.235723000	-6.396698000	-3.508079000
8	-2.959184000	-7.217998000	-2.606448000
1	-3.535676000	-7.793907000	-2.034949000
1	-2.502848000	-6.626738000	-1.972415000
8	-1.889882000	-5.494914000	-0.633139000
1	-1.158436000	-4.843898000	-0.511499000
1	-2.227228000	-5.681629000	0.276984000
8	-6.805755000	-7.443719000	-2.368623000
1	-7.220287000	-6.795174000	-1.758660000
1	-6.241464000	-6.903142000	-2.982856000

8	-4.613712000	-8.057439000	1.604841000
1	-4.100193000	-7.216521000	1.677465000
1	-3.999342000	-8.734781000	1.930513000
8	-3.106768000	-5.740798000	1.836501000
1	-2.508138000	-5.760270000	2.600022000
1	-3.490676000	-4.816815000	1.817036000
8	-3.661542000	-3.514042000	-1.255222000
1	-3.831289000	-3.293406000	-0.298294000
1	-3.121518000	-4.344674000	-1.181471000

$Re_{2PI,Br}$:

6	-4.494670000	-0.833446000	7.266367000
6	-4.737543000	-1.766082000	6.239916000
6	-4.498340000	-1.386372000	4.885136000
6	-4.021412000	-0.115487000	4.541403000
6	-3.783682000	0.782081000	5.576550000
6	-4.018694000	0.427660000	6.926713000
6	-5.199032000	-3.115353000	6.206161000
6	-5.234249000	-3.515447000	4.880072000
7	-4.806035000	-2.460428000	4.085102000
1	-4.668309000	-1.099666000	8.305941000
1	-3.844210000	0.157352000	3.507016000
1	-3.387078000	1.766967000	5.353903000
1	-3.804278000	1.156482000	7.703113000
1	-5.463341000	-3.720321000	7.061881000
6	-5.625614000	-4.815092000	4.330301000
6	-6.224563000	-5.783243000	5.166578000
6	-5.411249000	-5.155038000	2.980521000
6	-6.597983000	-7.030335000	4.671242000
6	-5.776247000	-6.414138000	2.487940000
6	-6.375954000	-7.356446000	3.327982000
1	-6.396196000	-5.554885000	6.213854000
1	-4.947832000	-4.445543000	2.302230000
1	-7.051764000	-7.756689000	5.340168000
1	-5.546351000	-6.665736000	1.454344000
1	-6.656859000	-8.332645000	2.946578000
35	-8.822565000	-1.300504000	1.398827000
8	-7.755957000	0.295616000	1.216317000
1	-7.370310000	0.536216000	2.091984000
1	-4.737475000	-2.434301000	3.062152000
8	-10.622270000	-0.651187000	-1.719080000
1	-10.027330000	0.067005000	-1.405965000
1	-10.067962000	-1.466249000	-1.737964000
8	-8.995803000	1.558901000	-0.877595000
1	-8.114163000	1.696249000	-1.297813000
1	-8.735895000	1.280919000	0.029961000
8	-12.169671000	-1.979634000	0.054160000
1	-11.674156000	-1.361897000	-0.555174000
1	-12.464314000	-1.425569000	0.816890000
8	-5.773497000	-0.532804000	-0.447532000
1	-6.511251000	-0.332686000	0.184908000
1	-6.156815000	-1.172982000	-1.103333000
8	-8.481386000	-7.061571000	0.450359000
1	-9.440098000	-6.816453000	0.556976000
1	-8.008928000	-6.506618000	1.093414000
8	-9.363976000	-3.144294000	-1.561624000
1	-8.458115000	-2.915914000	-1.890606000
1	-9.275613000	-3.239891000	-0.592341000
8	-10.084524000	-3.210704000	1.164721000
1	-10.923468000	-2.788145000	0.810530000
1	-10.350036000	-3.713035000	1.987972000
8	-11.773363000	-4.555658000	-1.165783000
1	-12.151421000	-3.748950000	-0.755866000
1	-10.927003000	-4.231975000	-1.543228000
8	-11.158849000	-4.711244000	3.074990000
1	-10.516245000	-5.016307000	3.767173000
1	-11.262656000	-5.423995000	2.399530000
8	-11.094176000	-6.282750000	0.798812000
1	-11.698790000	-7.034024000	0.684796000
1	-11.345716000	-5.630898000	0.076239000
8	-6.248434000	1.955790000	-1.392572000
1	-5.873041000	2.160679000	-2.264243000
1	-5.946125000	1.030007000	-1.178132000
8	-4.250239000	-2.093092000	1.260166000
1	-4.762640000	-1.474571000	0.679247000
1	-3.326133000	-1.723146000	1.308347000
8	-5.684137000	-4.578450000	-2.441438000
1	-5.088815000	-4.541731000	-1.648952000
1	-6.461770000	-5.150866000	-2.190770000
8	-6.879201000	-2.245721000	-2.299754000
1	-6.909480000	-1.816066000	-3.170015000
1	-6.348127000	-3.095642000	-2.429251000
8	-7.626593000	-6.432329000	-2.161884000
1	-7.864783000	-6.682097000	-1.240966000
1	-6.897129000	-7.038299000	-2.436291000
1	-12.243121000	4.468563000	1.809804000
1	-13.452580000	3.507602000	1.704696000
1	-13.476025000	-0.192434000	2.544713000
1	-11.992981000	0.291402000	2.429074000
1	-10.810372000	4.965513000	0.109191000
1	-11.663226000	6.232814000	0.467679000
1	-9.259723000	5.716679000	1.411615000
1	-8.580291000	4.401453000	1.881312000
1	-12.206346000	-2.264105000	5.882194000
1	-13.291540000	-2.530289000	6.979083000
1	-10.291591000	2.017836000	2.678809000
1	-11.793240000	2.509406000	2.706097000
1	-12.051019000	-1.609963000	3.616521000
1	-11.736837000	-3.139536000	3.830062000
1	-9.578003000	3.277815000	-0.899359000
1	-8.959473000	4.573939000	-0.332728000
1	-10.882797000	1.304795000	4.601395000
1	-9.870768000	1.843830000	5.640933000
1	-7.901189000	2.512272000	2.738336000
1	-8.789691000	3.238576000	3.783371000
1	-12.032055000	-0.579075000	7.455259000
1	-11.417051000	0.697677000	6.815169000
1	-9.059062000	4.114528000	5.996168000
1	-7.854756000	3.123421000	5.986828000
8	-12.357952000	-2.289311000	6.868339000
8	-12.580384000	-0.515249000	2.354001000
8	-11.685403000	-2.261019000	4.268609000
8	-11.793159000	0.352796000	7.670944000
8	-8.734546000	3.303587000	5.573200000
8	-10.455474000	1.076925000	5.460103000
8	-12.801081000	3.842644000	2.340841000
8	-11.211926000	1.716642000	2.878476000
8	-11.184935000	5.492239000	0.873518000
8	-9.658537000	4.260667000	-0.950049000
8	-8.416767000	5.222755000	1.339031000
8	-8.768650000	2.991757000	2.826190000
1	-5.842039000	2.112406000	2.059576000
1	-5.970962000	2.286483000	3.594492000
1	-5.670674000	3.871784000	5.304714000
1	-4.452348000	4.011025000	4.283458000
1	-5.305148000	3.912638000	1.126975000

1	-5.417386000	2.789061000	-0.012330000
1	-5.991321000	1.915697000	6.674685000
1	-6.891288000	2.612712000	7.767417000
1	-5.883848000	4.887850000	2.977216000
1	-6.761175000	5.525420000	1.835882000
8	-5.428635000	3.812543000	4.363524000
8	-6.346131000	2.787465000	6.934181000
8	-6.336605000	1.755337000	2.846317000
8	-5.003119000	3.007057000	0.858080000
8	-5.825614000	5.328931000	2.095008000
1	-4.768245000	3.532349000	7.610469000
1	-3.760917000	3.511763000	8.784757000
8	-3.900117000	3.889628000	7.902805000
1	-9.164368000	-3.970569000	5.021020000
1	-8.661896000	-5.465325000	5.080918000
1	-8.837956000	-2.119736000	5.854005000
1	-9.862628000	-2.098314000	4.646496000
1	-10.155305000	-4.591074000	6.867867000
1	-11.206728000	-3.600795000	7.436575000
1	-10.394634000	0.254646000	8.488331000
1	-9.657381000	-0.114588000	9.832046000
1	-9.245882000	-2.874474000	7.764607000
1	-8.918039000	-1.358314000	8.040568000
1	-8.473389000	1.582879000	8.954185000
1	-7.349302000	2.200269000	9.829741000
8	-10.459346000	-4.181704000	7.709205000
8	-9.468171000	-4.926230000	5.155029000
8	-8.942627000	-2.347484000	4.888943000
8	-8.594085000	-2.156199000	7.564531000
8	-9.479652000	0.144319000	8.913227000
8	-7.892580000	2.384143000	9.047294000
1	-2.638630000	3.986211000	3.014982000
1	-3.053251000	5.441939000	3.479641000
1	-1.163766000	-2.067823000	1.219099000
1	-1.374101000	-0.544759000	0.863559000
1	0.418836000	-3.667670000	0.427873000
1	-0.853333000	-3.420947000	-0.463791000
1	-1.222221000	1.738008000	0.395873000
1	-0.236642000	1.064857000	1.301550000
1	-4.523162000	6.492818000	2.413299000
1	-3.157297000	7.021673000	1.914853000
1	-3.286730000	2.771318000	1.354487000
1	-2.125142000	2.116847000	2.244735000
1	0.537263000	1.735000000	5.615050000
1	-0.896308000	2.266736000	5.885344000
1	-1.289500000	0.015842000	2.921491000
1	-0.819704000	1.066356000	4.036131000
1	-2.046040000	4.091051000	5.328325000
1	-2.460549000	3.727643000	6.788217000
8	-1.677214000	-1.262881000	1.472883000
8	-0.543853000	1.031946000	0.371248000
8	-0.549851000	-3.618182000	0.471133000
8	-1.049887000	0.952030000	3.073591000
8	-2.357447000	2.886960000	1.667970000
8	-3.674964000	6.896344000	2.726117000
8	-2.893900000	4.519015000	3.815301000
8	-1.687038000	3.745255000	6.178642000
8	-0.385580000	1.438621000	5.658291000
8	-4.839809000	-9.765044000	0.741115000
1	-4.437749000	-9.720184000	1.641665000
1	-5.815104000	-9.712738000	0.881952000
8	-1.745099000	-3.161751000	-1.873477000
1	-2.602585000	-3.484813000	-1.483422000

1	-1.496828000	-3.849017000	-2.512392000
8	-5.176627000	-7.367839000	-3.062866000
1	-5.105819000	-6.400305000	-3.185943000
1	-4.759730000	-7.487918000	-2.169884000
8	-4.288940000	-7.444271000	-0.502650000
1	-4.419813000	-8.314953000	-0.032625000
1	-3.406706000	-7.111008000	-0.250645000
8	-2.127210000	-5.925597000	0.632834000
1	-1.418137000	-5.239851000	0.625459000
1	-2.235736000	-6.196124000	1.571840000
8	-7.613206000	-9.623956000	1.109730000
1	-7.978162000	-8.748934000	0.833222000
1	-8.006564000	-10.263144000	0.495381000
8	-3.541689000	-9.582556000	3.199909000
1	-3.192101000	-8.663682000	3.222230000
1	-2.752939000	-10.133167000	3.072325000
8	-2.628793000	-6.900909000	3.214181000
1	-1.958092000	-6.712396000	3.892204000
1	-3.414408000	-6.388538000	3.481884000
8	-3.865288000	-4.132908000	-0.479423000
1	-4.044638000	-3.412057000	0.185379000
1	-3.372280000	-4.843703000	0.005816000

$TS_{2PI,Br}$:

6	-4.497075000	-0.282451000	6.099630000
6	-4.598925000	-1.295804000	5.119795000
6	-3.567645000	-1.448849000	4.149735000
6	-2.448501000	-0.630762000	4.120773000
6	-2.373191000	0.366083000	5.100249000
6	-3.379610000	0.537964000	6.077335000
6	-5.583997000	-2.252659000	4.821068000
6	-5.101262000	-3.021231000	3.700380000
7	-3.905927000	-2.511668000	3.322822000
1	-5.285701000	-0.154421000	6.835171000
1	-1.671886000	-0.744894000	3.371216000
1	-1.507172000	1.019117000	5.118673000
1	-3.272929000	1.331970000	6.810003000
1	-6.500582000	-2.448945000	5.362658000
6	-5.663319000	-4.243212000	3.152103000
6	-6.802629000	-4.811624000	3.756313000
6	-5.064174000	-4.913508000	2.062369000
6	-7.290666000	-6.040950000	3.320189000
6	-5.561126000	-6.140809000	1.629463000
6	-6.663634000	-6.722184000	2.275048000
1	-7.297580000	-4.300561000	4.574162000
1	-4.226418000	-4.465657000	1.536735000
1	-8.162268000	-6.468602000	3.805660000
1	-5.095175000	-6.639370000	0.782334000
1	-7.031823000	-7.692298000	1.955347000
35	-7.250407000	-1.329405000	2.630050000
8	-5.165134000	0.148711000	2.443132000
1	-5.835333000	0.827952000	2.645851000
8	-10.427452000	-0.479339000	-1.451061000
1	-9.775759000	0.038004000	-0.923219000
1	-9.980563000	-1.356113000	-1.555165000
8	-8.460462000	0.860914000	0.021345000
1	-7.682788000	0.867806000	-0.585785000
1	-8.182341000	0.335828000	0.798188000
8	-11.978256000	-1.639493000	0.407334000
1	-11.521773000	-1.060619000	-0.266726000
1	-12.206238000	-1.063923000	1.175961000
8	-4.907424000	-0.653228000	-0.002753000
1	-5.140329000	-0.337943000	0.945814000

1	-5.596137000	-1.300192000	-0.280171000
8	-8.416457000	-6.602019000	-0.450002000
1	-9.333072000	-6.399735000	-0.118783000
1	-7.835376000	-6.089293000	0.137114000
8	-9.223749000	-2.980204000	-1.316303000
1	-8.295255000	-2.757972000	-1.571882000
1	-9.211895000	-2.947190000	-0.322564000
8	-9.563741000	-2.573468000	1.313547000
1	-10.470014000	-2.223249000	1.114799000
1	-9.707356000	-3.293820000	1.967338000
8	-11.761904000	-4.056476000	-1.004972000
1	-12.024296000	-3.285951000	-0.452049000
1	-10.901704000	-3.774395000	-1.387446000
8	-10.638176000	-4.499138000	2.979860000
1	-10.405818000	-5.025582000	3.778126000
1	-10.843503000	-5.121929000	2.242593000
8	-10.864471000	-5.949497000	0.626715000
1	-11.480236000	-6.699663000	0.591663000
1	-11.259911000	-5.250541000	0.015726000
8	-6.151356000	1.166746000	-1.467966000
1	-5.889229000	1.252081000	-2.398130000
1	-5.502033000	0.544876000	-1.023372000
8	-3.071197000	-2.521952000	0.693495000
1	-3.623858000	-1.741999000	0.414733000
1	-2.122653000	-2.206495000	0.820403000
8	-5.433173000	-3.976823000	-2.994753000
1	-4.708343000	-4.184683000	-2.353184000
1	-6.180510000	-4.610784000	-2.824099000
8	-6.670918000	-2.036672000	-1.649239000
1	-6.771107000	-1.223081000	-2.170755000
1	-6.139566000	-2.667803000	-2.221993000
8	-7.453415000	-5.800783000	-2.990139000
1	-7.800456000	-6.122498000	-2.129722000
1	-6.759994000	-6.442140000	-3.274583000
1	-12.348251000	4.232102000	0.553395000
1	-13.366592000	3.072130000	0.451295000
1	-13.041996000	-0.233536000	3.171472000
1	-11.886971000	0.738610000	2.764756000
1	-10.348147000	4.331975000	-0.563715000
1	-11.302632000	5.446959000	-1.116450000
1	-9.521153000	5.827015000	0.665849000
1	-8.922015000	5.088599000	1.900243000
1	-12.101066000	-2.028060000	5.534631000
1	-13.617610000	-2.042512000	5.931887000
1	-10.718549000	2.755565000	2.845136000
1	-12.188737000	2.887285000	2.283153000
1	-11.216929000	-1.362887000	3.730899000
1	-11.048607000	-2.925759000	3.781091000
1	-8.795368000	2.659141000	-0.220888000
1	-8.455855000	4.172703000	-0.019562000
1	-11.923144000	2.572506000	4.707410000
1	-11.013999000	3.102922000	5.833968000
1	-8.430016000	3.225726000	2.879285000
1	-9.319391000	3.979301000	3.920886000
1	-12.444512000	-0.229654000	6.910162000
1	-12.084950000	1.305553000	6.671640000
1	-9.161569000	4.733623000	6.140940000
1	-8.504506000	3.346093000	5.818160000
8	-12.729403000	-1.934263000	6.308456000
8	-12.164566000	-0.226476000	2.755877000
8	-10.949935000	-2.109342000	4.325150000
8	-12.194654000	0.604799000	7.369332000
8	-9.326508000	3.892758000	5.685830000
8	-11.904063000	2.718607000	5.682437000
8	-13.046747000	3.751042000	1.066318000
8	-11.629842000	2.369133000	2.926031000
8	-11.018742000	5.020839000	-0.291755000
8	-8.823031000	3.534030000	-0.667863000
8	-8.678452000	5.690895000	1.148818000
8	-9.275695000	3.747110000	2.958488000
1	-6.564686000	2.547770000	1.898628000
1	-6.291823000	3.303332000	3.206572000
1	-5.338358000	4.753803000	4.562259000
1	-4.440291000	4.481727000	3.308027000
1	-5.701155000	3.969987000	0.485270000
1	-5.879067000	2.614732000	-0.378586000
1	-7.006665000	1.986432000	4.906479000
1	-7.214378000	1.649092000	6.455219000
1	-5.858612000	5.492905000	2.057220000
1	-6.887276000	5.867342000	0.946627000
8	-5.379107000	4.691815000	3.577847000
8	-7.069470000	2.389272000	5.793831000
8	-6.844494000	2.550449000	2.851091000
8	-5.579233000	2.987405000	0.480639000
8	-5.937039000	5.644560000	1.084696000
1	-5.648929000	3.383160000	6.146754000
1	-5.244302000	4.661391000	6.946285000
8	-4.967074000	4.102305000	6.203238000
1	-9.504822000	-4.506872000	5.697721000
1	-9.641391000	-6.059181000	5.925425000
1	-8.955087000	-2.815476000	6.782198000
1	-9.321215000	-2.490400000	5.269444000
1	-11.297948000	-4.656677000	6.937273000
1	-12.199258000	-3.425445000	7.217792000
1	-10.788329000	0.276290000	8.052156000
1	-10.050149000	0.298028000	9.449088000
1	-10.330555000	-3.231174000	8.248449000
1	-9.737569000	-1.788277000	8.489289000
1	-8.364381000	0.404019000	7.954479000
1	-7.025546000	0.987124000	8.487959000
8	-11.730559000	-4.165098000	7.669921000
8	-10.117797000	-5.292733000	5.568035000
8	-8.721221000	-3.030743000	5.829943000
8	-9.481828000	-2.733167000	8.372187000
8	-9.936649000	-0.002159000	8.533330000
8	-7.414655000	0.565618000	7.705270000
1	-2.918877000	3.315268000	2.392299000
1	-2.859060000	4.859568000	1.997690000
1	-0.095928000	-2.363469000	0.566050000
1	-0.529277000	-0.865706000	0.995120000
1	1.156890000	-4.099669000	-0.685801000
1	-0.125261000	-3.577499000	-1.420233000
1	-1.657302000	1.133429000	1.065957000
1	-0.284134000	1.181183000	1.790369000
1	-4.201565000	6.036682000	0.690208000
1	-3.032632000	5.491204000	-0.152808000
1	-3.979850000	2.255976000	0.977189000
1	-3.848461000	1.168489000	2.067713000
1	0.600821000	3.321549000	5.947720000
1	-0.918177000	3.413962000	5.595710000
1	1.008209000	0.805111000	3.637353000
1	0.309134000	2.114915000	4.067005000
1	-2.359767000	4.471904000	4.464121000
1	-3.073898000	4.192980000	5.808242000
8	-0.588796000	-1.831447000	1.228115000
8	-0.717043000	0.862256000	0.962638000

8	0.219247000	-3.897091000	-0.536837000
8	0.636936000	1.621806000	3.270632000
8	-3.301453000	1.840999000	1.563660000
8	-3.218867000	5.972470000	0.669025000
8	-2.861407000	4.234257000	2.769662000
8	-2.215960000	4.488538000	5.438752000
8	-0.140275000	2.787864000	5.621686000
8	-4.834008000	-9.583375000	-0.304338000
1	-4.421560000	-9.590642000	0.592772000
1	-5.797273000	-9.435286000	-0.147956000
8	-1.031735000	-3.138182000	-2.791426000
1	-1.899279000	-3.461955000	-2.423003000
1	-0.814729000	-3.776438000	-3.489770000
8	-5.054460000	-6.835627000	-3.893660000
1	-4.898855000	-5.876686000	-3.976840000
1	-4.610177000	-7.057584000	-3.033124000
8	-4.020497000	-7.281586000	-1.417575000
1	-4.260858000	-8.167868000	-1.023630000
1	-3.069692000	-7.143958000	-1.252684000
8	-1.562575000	-6.095989000	-0.439945000
1	-0.825270000	-5.439522000	-0.424215000
1	-1.766974000	-6.297482000	0.499251000
8	-7.558750000	-9.177481000	0.154703000
1	-7.903614000	-8.301580000	-0.144664000
1	-8.086666000	-9.834649000	-0.324635000
8	-3.509587000	-9.539983000	2.144417000
1	-3.055345000	-8.667850000	2.148931000
1	-2.791702000	-10.180115000	2.016606000
8	-2.322958000	-6.964411000	2.150165000
1	-1.614318000	-6.905293000	2.813120000
1	-3.040115000	-6.406632000	2.501202000
8	-3.178120000	-4.127780000	-1.463404000
1	-3.183787000	-3.516064000	-0.676099000
1	-2.748292000	-4.948291000	-1.111815000
1	-3.478969000	-2.661056000	2.388366000

$Int_{2PI,Br}$:

6	-5.744762000	-0.363556000	8.809536000
6	-5.356547000	-1.334799000	7.895428000
6	-4.054619000	-1.845400000	7.940173000
6	-3.096107000	-1.427774000	8.850810000
6	-3.493292000	-0.438685000	9.757883000
6	-4.794132000	0.077674000	9.741313000
6	-6.081702000	-2.027453000	6.784972000
6	-5.080608000	-3.053552000	6.293629000
7	-3.955301000	-2.860687000	6.960446000
1	-6.750786000	0.045908000	8.819113000
1	-2.096322000	-1.847848000	8.866013000
1	-2.780112000	-0.076205000	10.491303000
1	-5.076585000	0.833611000	10.467325000
1	-7.083388000	-2.427046000	6.978956000
6	-5.308180000	-4.107719000	5.340010000
6	-4.300092000	-5.053684000	5.033667000
6	-6.580672000	-4.238421000	4.738309000
6	-4.563160000	-6.093604000	4.156028000
6	-6.832124000	-5.285868000	3.859948000
6	-5.827981000	-6.211033000	3.563083000
1	-3.314349000	-4.988254000	5.483067000
1	-7.368877000	-3.534580000	4.973590000
1	-3.792748000	-6.819304000	3.920303000
1	-7.813580000	-5.384531000	3.407555000
1	-6.036954000	-7.014130000	2.862288000
35	-6.417794000	-0.727598000	5.274874000
8	-8.763425000	-0.455898000	2.596606000
1	-8.327582000	0.170486000	3.204349000
1	-3.109230000	-3.403771000	6.819667000
8	-11.186646000	0.086811000	-1.025445000
1	-10.321539000	0.340029000	-0.596426000
1	-10.933315000	-0.039322000	-1.980810000
8	-8.891021000	0.793405000	0.169075000
1	-8.052433000	0.406275000	-0.218054000
1	-8.913742000	0.431177000	1.096820000
8	-11.339032000	-2.203193000	0.407011000
1	-11.297915000	-1.451074000	-0.248219000
1	-11.600083000	-1.750751000	1.251459000
8	-6.855427000	-2.092460000	1.454102000
1	-8.034931000	-1.075707000	2.333541000
1	-7.607586000	-2.673178000	1.181242000
8	-9.282361000	-6.116909000	-0.003774000
1	-10.172101000	-6.308899000	0.379406000
1	-9.119145000	-5.187050000	0.308642000
8	-10.094290000	-0.410753000	-3.460227000
1	-9.347870000	-0.897652000	-2.998326000
1	-10.607580000	-1.118572000	-3.881092000
8	-9.111568000	-3.631832000	1.083834000
1	-9.866400000	-3.066605000	0.762200000
1	-9.373662000	-3.901551000	1.993860000
8	-13.030170000	-4.283944000	0.036254000
1	-12.456654000	-3.472693000	0.117843000
1	-12.940591000	-4.549576000	-0.892820000
8	-10.304879000	-4.690228000	3.376240000
1	-10.187021000	-5.137664000	4.250513000
1	-10.906001000	-5.251447000	2.825545000
8	-11.644127000	-6.092349000	1.430479000
1	-12.208140000	-6.859454000	1.616945000
1	-12.218532000	-5.443153000	0.922411000
8	-6.608106000	-0.350960000	-0.416584000
1	-5.737626000	-0.352665000	-0.856930000
1	-6.711619000	-1.425585000	0.671384000
8	-4.729925000	-3.591445000	0.816735000
1	-5.517944000	-3.096486000	1.189098000
1	-3.946337000	-2.985749000	0.917464000
8	-7.039915000	-3.910480000	-3.369121000
1	-6.265587000	-3.952969000	-2.755694000
1	-7.738726000	-4.503685000	-2.985142000
8	-8.278028000	-1.771990000	-2.052972000
1	-7.612901000	-1.253881000	-1.519556000
1	-7.770400000	-2.423625000	-2.589820000
8	-8.885200000	-5.833284000	-2.751445000
1	-9.014205000	-6.031924000	-1.795781000
1	-8.170171000	-6.430989000	-3.075004000
1	-11.995112000	3.077827000	-0.191319000
1	-12.111624000	1.531947000	-0.415097000
1	-12.924655000	-0.889921000	2.907805000
1	-11.761384000	0.127442000	2.619736000
1	-10.057389000	3.974987000	-0.838373000
1	-11.141499000	5.033566000	-1.252684000
1	-9.611785000	5.202909000	0.781820000
1	-9.024590000	4.414653000	1.994989000
1	-12.256074000	-2.346341000	5.538099000
1	-13.818968000	-2.492587000	5.587276000
1	-10.708754000	2.109346000	2.612134000
1	-11.992960000	2.016963000	1.662666000
1	-11.073740000	-1.646885000	3.950723000
1	-10.781783000	-3.172510000	4.120001000
1	-8.670701000	2.408166000	-0.373322000

1 -8.268996000 3.870752000 -0.063780000
1 -12.210897000 2.095270000 4.271693000
1 -11.441859000 2.652833000 5.491638000
1 -8.417057000 2.451778000 3.070250000
1 -9.422086000 3.267924000 3.939676000
1 -13.021299000 -0.543548000 6.649719000
1 -12.681677000 0.974922000 6.314925000
1 -9.731374000 4.333441000 5.954180000
1 -8.963984000 2.975991000 6.091679000
8 -13.051546000 -2.295172000 6.148058000
8 -11.978976000 -0.853886000 2.692007000
8 -10.864141000 -2.330809000 4.637713000
8 -12.930468000 0.352411000 7.048750000
8 -9.741085000 3.412426000 5.648675000
8 -12.315243000 2.289944000 5.231192000
8 -12.511476000 2.284587000 0.084758000
8 -11.631362000 1.751125000 2.560774000
8 -10.867374000 4.465717000 -0.514650000
8 -8.501464000 3.283006000 -0.810473000
8 -8.818901000 5.147267000 1.357612000
8 -9.237903000 3.005312000 3.001136000
1 -6.537817000 1.364537000 2.615025000
1 -6.460104000 2.240816000 3.875650000
1 -5.536012000 3.973162000 4.905065000
1 -4.627522000 3.593710000 3.667437000
1 -6.041389000 2.475133000 0.891656000
1 -6.136912000 0.886985000 0.497251000
1 -7.345921000 1.421049000 6.377235000
1 -7.993662000 1.921933000 7.713797000
1 -6.112220000 4.292018000 2.364058000
1 -7.010671000 4.915041000 1.256753000
8 -5.574929000 3.706240000 3.954950000
8 -7.588556000 2.238170000 6.850614000
8 -6.987276000 1.483730000 3.500814000
8 -5.753876000 1.574184000 1.150433000
8 -6.150442000 4.455684000 1.390319000
1 -6.072975000 3.178557000 6.864700000
1 -5.329356000 4.508980000 7.220571000
8 -5.277186000 3.728721000 6.646515000
1 -9.604797000 -4.511899000 6.283752000
1 -9.609078000 -6.082326000 6.368088000
1 -9.472598000 -2.769526000 7.477503000
1 -9.560350000 -2.483206000 5.916519000
1 -11.565474000 -4.834978000 7.200642000
1 -12.619193000 -3.700107000 7.226798000
1 -11.759789000 0.195091000 8.136539000
1 -11.564507000 0.201911000 9.697967000
1 -11.069522000 -3.262885000 8.627399000
1 -10.683307000 -1.753725000 8.902748000
1 -9.587886000 0.882444000 8.943772000
1 -8.843141000 2.001867000 9.740154000
8 -12.187802000 -4.372318000 7.804353000
8 -10.129002000 -5.331668000 6.038624000
8 -9.024060000 -2.956080000 6.596300000
8 -10.321587000 -2.671012000 8.901379000
8 -11.084463000 0.015771000 8.874870000
8 -8.696880000 1.303511000 9.083024000
1 -2.954703000 3.034275000 2.302003000
1 -3.154751000 4.567736000 2.548023000
1 -1.924894000 -2.522016000 0.200865000
1 -2.947956000 -1.338019000 0.208914000
1 -0.288823000 -3.298178000 -1.470282000
1 -1.697517000 -2.824544000 -1.971685000
1 -3.308231000 0.634903000 -0.514408000
1 -3.299395000 -0.466807000 -1.687551000
1 -4.665900000 5.442348000 1.327738000
1 -3.305680000 5.702885000 0.636644000
1 -4.006393000 1.649292000 1.141764000
1 -2.575292000 1.108015000 1.584256000
1 -3.986391000 0.068988000 4.894819000
1 -2.902327000 1.114388000 5.266028000
1 -1.938358000 -0.841444000 2.085941000
1 -2.231583000 -0.017278000 3.405528000
1 -2.704459000 3.216216000 4.793309000
1 -3.554344000 3.086305000 6.090294000
8 -2.531198000 -2.024852000 0.799830000
8 -3.707475000 -0.204884000 -0.833728000
8 -1.239488000 -3.400646000 -1.303211000
8 -1.732649000 0.000846000 2.549372000
8 -3.032375000 1.799363000 1.020780000
8 -3.768855000 5.836229000 1.478928000
8 -3.030598000 3.707284000 3.029075000
8 -2.713223000 2.792912000 5.680123000
8 -3.024044000 0.168526000 4.980706000
8 -5.452610000 -8.936080000 -0.392768000
1 -4.786287000 -8.866007000 0.329268000
1 -6.242858000 -8.506218000 0.012613000
8 -2.945063000 -1.926020000 -2.788973000
1 -3.696656000 -2.539709000 -2.538038000
1 -2.916128000 -1.918064000 -3.758861000
8 -6.630189000 -6.736018000 -4.057143000
1 -6.526253000 -5.765028000 -4.093127000
1 -5.855925000 -7.054377000 -3.520068000
8 -4.501837000 -7.725849000 -2.655933000
1 -4.846346000 -8.110793000 -1.806659000
1 -3.854518000 -7.043589000 -2.387903000
8 -2.809862000 -5.673155000 -1.601119000
1 -2.078678000 -5.014217000 -1.652515000
1 -2.926429000 -5.807660000 -0.628003000
8 -7.482370000 -7.806928000 1.128711000
1 -8.136220000 -7.179323000 0.718922000
1 -8.020861000 -8.546817000 1.449125000
8 -3.770235000 -8.438024000 1.787922000
1 -3.602638000 -7.493704000 1.556211000
1 -2.900861000 -8.862805000 1.714146000
8 -3.283031000 -5.807822000 1.103888000
1 -2.515057000 -5.474332000 1.595654000
1 -3.958054000 -5.072268000 1.147886000
8 -4.725688000 -3.746393000 -1.895229000
1 -4.849384000 -3.595825000 -0.917664000
1 -4.126651000 -4.539638000 -1.911423000

$Re_{MPI,Br}$:

6 -3.234873000 -1.143869000 7.315440000
6 -3.951667000 -1.876313000 6.349158000
6 -4.404600000 -1.213214000 5.171636000
6 -4.152674000 0.147576000 4.941212000
6 -3.435173000 0.841986000 5.913351000
6 -2.985655000 0.202982000 7.091624000
6 -4.366831000 -3.236248000 6.231120000
6 -5.032876000 -3.366567000 5.025783000
7 -5.064241000 -2.131244000 4.381355000
1 -2.879087000 -1.630710000 8.220101000
1 -4.470778000 0.636095000 4.024966000
1 -3.180694000 1.883404000 5.747077000
1 -2.422732000 0.776925000 7.822726000

1	-4.154641000	-4.044286000	6.918185000
6	-5.562752000	-4.607257000	4.441818000
6	-6.182442000	-5.557001000	5.276375000
6	-5.404778000	-4.921375000	3.076623000
6	-6.625359000	-6.778013000	4.764985000
6	-5.850833000	-6.144667000	2.565708000
6	-6.466268000	-7.077683000	3.406991000
1	-6.324739000	-5.324323000	6.327380000
1	-4.908936000	-4.219287000	2.413060000
1	-7.091650000	-7.499820000	5.430682000
1	-5.668210000	-6.383298000	1.519923000
1	-6.814179000	-8.025310000	3.007785000
6	-5.831896000	-1.771496000	3.190316000
1	-5.220504000	-1.809272000	2.282168000
1	-6.695115000	-2.429066000	3.099554000
1	-6.206711000	-0.753469000	3.318621000
35	-8.531615000	-1.175943000	0.692510000
8	-7.467005000	0.422086000	0.496541000
1	-7.074732000	0.648995000	1.371528000
8	-10.437482000	-0.429400000	-2.331362000
1	-9.853306000	0.296968000	-2.016449000
1	-9.866924000	-1.231585000	-2.380574000
8	-8.862205000	1.786268000	-1.438934000
1	-8.007585000	1.993015000	-1.886053000
1	-8.546931000	1.455849000	-0.567391000
8	-11.920016000	-1.816682000	-0.557671000
1	-11.446774000	-1.171343000	-1.156335000
1	-12.229870000	-1.295638000	0.222600000
8	-5.610112000	-0.240749000	-1.403815000
1	-6.290513000	-0.136473000	-0.692544000
1	-5.988600000	-0.904597000	-2.037258000
8	-8.432593000	-6.718669000	0.111483000
1	-9.408958000	-6.527542000	0.064509000
1	-8.084330000	-6.069375000	0.744418000
8	-9.171285000	-2.926568000	-2.276977000
1	-8.274204000	-2.730783000	-2.648836000
1	-9.035226000	-3.061484000	-1.318899000
8	-9.818986000	-3.082541000	0.489407000
1	-10.656648000	-2.642794000	0.152760000
1	-10.090209000	-3.586678000	1.308019000
8	-11.611190000	-4.300369000	-1.899798000
1	-11.960556000	-3.498620000	-1.453692000
1	-10.750790000	-3.991747000	-2.258318000
8	-11.008017000	-4.580409000	2.345346000
1	-10.467817000	-5.014527000	3.054282000
1	-11.209845000	-5.247600000	1.645857000
8	-11.083984000	-6.073878000	0.049247000
1	-11.670601000	-6.828764000	-0.118743000
1	-11.273784000	-5.413010000	-0.685780000
8	-6.163143000	2.317871000	-2.028976000
1	-5.832886000	2.636814000	-2.884330000
1	-5.849812000	1.373534000	-1.951225000
8	-4.041513000	-1.832839000	0.245474000
1	-4.468183000	-1.185002000	-0.363542000
1	-3.153475000	-1.464361000	0.488328000
8	-5.506400000	-4.485944000	-3.016505000
1	-4.864998000	-4.405150000	-2.261472000
1	-6.286950000	-5.005884000	-2.680185000
8	-6.680039000	-2.127639000	-3.120489000
1	-6.698746000	-1.810032000	-4.037954000
1	-6.151924000	-2.988510000	-3.140780000
8	-7.497091000	-6.248288000	-2.504173000
1	-7.741096000	-6.416931000	-1.566672000

1	-6.788086000	-6.900000000	-2.721185000
1	-12.098859000	4.517029000	1.530100000
1	-13.308125000	3.552648000	1.457669000
1	-13.291831000	-0.130454000	1.952313000
1	-11.801127000	0.342890000	1.893354000
1	-10.748685000	5.094422000	-0.218410000
1	-11.587823000	6.340970000	0.233694000
1	-9.165873000	5.788788000	1.063417000
1	-8.433930000	4.465643000	1.421013000
1	-12.077234000	-2.297355000	5.317337000
1	-13.150456000	-2.519036000	6.436804000
1	-10.087243000	2.033957000	2.167103000
1	-11.584817000	2.526713000	2.301596000
1	-11.908573000	-1.572092000	3.058915000
1	-11.614308000	-3.110773000	3.203939000
1	-9.521145000	3.477208000	-1.351132000
1	-8.911012000	4.762584000	-0.758015000
1	-10.603907000	1.218225000	4.082955000
1	-9.562458000	1.727091000	5.104177000
1	-7.682605000	2.533321000	2.160955000
1	-8.545175000	3.198259000	3.265240000
1	-11.745370000	-0.668428000	6.926283000
1	-11.074402000	0.583902000	6.293598000
1	-8.828618000	4.001502000	5.494550000
1	-7.594468000	3.047131000	5.475535000
8	-12.207432000	-2.331082000	6.305948000
8	-12.392746000	-0.457684000	1.786970000
8	-11.580206000	-2.257169000	3.694814000
8	-11.438043000	0.239806000	7.155290000
8	-8.472464000	3.214759000	5.051640000
8	-10.145125000	0.959930000	4.916484000
8	-12.619620000	3.864119000	2.066250000
8	-10.996759000	1.725731000	2.399189000
8	-11.094308000	5.582330000	0.584225000
8	-9.629946000	4.458431000	-1.356609000
8	-8.314099000	5.323333000	0.927538000
8	-8.549044000	2.995544000	2.296973000
1	-5.582809000	2.225521000	1.464163000
1	-5.758167000	2.397075000	2.997095000
1	-5.503842000	3.877307000	4.794010000
1	-4.293611000	4.090077000	3.773394000
1	-5.186617000	4.094799000	0.592533000
1	-5.298885000	3.026985000	-0.598688000
1	-5.678937000	1.913109000	6.123512000
1	-6.605939000	2.519358000	7.246040000
1	-5.750019000	4.981903000	2.505716000
1	-6.660502000	5.641402000	1.405059000
8	-5.264387000	3.857827000	3.850198000
8	-6.083960000	2.753609000	6.412384000
8	-6.047559000	1.819110000	2.246934000
8	-4.861760000	3.215790000	0.267996000
8	-5.716269000	5.453061000	1.638357000
1	-4.559965000	3.605052000	7.122585000
1	-3.505758000	3.449620000	8.235454000
8	-3.735549000	4.007698000	7.476147000
1	-9.192343000	-4.179046000	4.437864000
1	-8.761131000	-5.691650000	4.505179000
1	-8.701369000	-2.359212000	5.272653000
1	-9.775070000	-2.234436000	4.102393000
1	-10.153029000	-4.746762000	6.278613000
1	-11.109346000	-3.682854000	6.873206000
1	-10.021917000	0.050891000	7.924978000
1	-9.270269000	-0.368557000	9.244124000

1	-9.069134000	-3.110884000	7.164896000
1	-8.619309000	-1.627955000	7.441026000
1	-8.112156000	1.353890000	8.411056000
1	-6.973745000	1.946995000	9.284871000
8	-10.392529000	-4.309382000	7.126672000
8	-9.547690000	-5.119922000	4.540543000
8	-8.876607000	-2.576062000	4.315073000
8	-8.363827000	-2.446354000	6.957359000
8	-9.101696000	-0.099304000	8.326373000
8	-7.553192000	2.165975000	8.538181000
1	-2.507410000	4.155782000	2.515374000
1	-2.952741000	5.596261000	3.004845000
1	-1.026556000	-1.902781000	0.831261000
1	-1.112495000	-0.369265000	0.458408000
1	0.538539000	-3.645374000	0.355896000
1	-0.571278000	-3.413202000	-0.733803000
1	-0.933611000	1.911509000	-0.006585000
1	-0.124761000	1.258173000	1.071427000
1	-4.442150000	6.640857000	1.971965000
1	-3.089432000	7.204918000	1.476181000
1	-3.150831000	2.987417000	0.794642000
1	-2.085236000	2.288693000	1.757167000
1	0.681887000	2.043479000	5.015756000
1	-0.731426000	2.540141000	5.423266000
1	-1.294237000	0.176324000	2.533648000
1	-0.803168000	1.249862000	3.632332000
1	-1.922383000	4.306979000	4.852628000
1	-2.309972000	3.994870000	6.333118000
8	-1.529179000	-1.072734000	1.014026000
8	-0.242702000	1.226588000	0.096125000
8	-0.419459000	-3.527719000	0.251000000
8	-1.100202000	1.122164000	2.690499000
8	-2.240007000	3.066216000	1.166393000
8	-3.600436000	7.055682000	2.287645000
8	-2.769474000	4.672181000	3.324919000
8	-1.542736000	4.015498000	5.714685000
8	-0.217265000	1.718597000	5.181802000
8	-4.947320000	-9.502903000	0.653082000
1	-4.523430000	-9.448605000	1.543196000
1	-5.916959000	-9.418426000	0.818394000
8	-1.206821000	-3.311588000	-2.290864000
1	-2.132072000	-3.563802000	-2.014567000
1	-0.883729000	-4.082456000	-2.784404000
8	-5.069256000	-7.364153000	-3.230626000
1	-4.955737000	-6.425779000	-3.480435000
1	-4.729716000	-7.375289000	-2.296647000
8	-4.400104000	-7.187137000	-0.601467000
1	-4.542178000	-8.055718000	-0.128940000
1	-3.494445000	-6.896401000	-0.382016000
8	-2.145831000	-5.731413000	0.418608000
1	-1.411514000	-5.073039000	0.419466000
1	-2.314721000	-5.945359000	1.362475000
8	-7.689553000	-9.215229000	1.084397000
1	-7.994017000	-8.353158000	0.709500000
1	-8.148061000	-9.890212000	0.560062000
8	-3.605224000	-9.300519000	3.086534000
1	-3.234124000	-8.389689000	3.081804000
1	-2.831679000	-9.872449000	2.959599000
8	-2.640616000	-6.640422000	3.035722000
1	-1.906750000	-6.478650000	3.652360000
1	-3.388540000	-6.122066000	3.386511000
8	-3.571523000	-4.010867000	-1.186183000
1	-3.781026000	-3.193941000	-0.638810000
1	-3.247356000	-4.683785000	-0.537813000

$TS_{MPI,Br}$:

6	-6.670724000	-0.754576000	6.671064000
6	-6.015480000	-1.475867000	5.669808000
6	-4.663347000	-1.207584000	5.374017000
6	-3.944521000	-0.203633000	6.007627000
6	-4.620004000	0.518029000	7.003906000
6	-5.951927000	0.235652000	7.346104000
6	-6.407208000	-2.570377000	4.829865000
6	-5.235592000	-3.000015000	4.128470000
7	-4.227304000	-2.158576000	4.424053000
1	-7.703348000	-0.971140000	6.919800000
1	-2.911046000	0.024314000	5.761893000
1	-4.093357000	1.312812000	7.523834000
1	-6.434473000	0.793415000	8.142360000
1	-7.318725000	-3.135644000	4.943877000
6	-5.174799000	-4.150627000	3.224164000
6	-5.779890000	-5.354890000	3.632518000
6	-4.587250000	-4.071425000	1.950263000
6	-5.760951000	-6.472185000	2.799877000
6	-4.584843000	-5.188875000	1.115539000
6	-5.155679000	-6.393906000	1.540593000
1	-6.241110000	-5.420004000	4.613485000
1	-4.170120000	-3.142274000	1.580084000
1	-6.215658000	-7.403635000	3.123213000
1	-4.153885000	-5.117117000	0.122730000
1	-5.115448000	-7.262922000	0.893350000
6	-2.937389000	-2.042100000	3.772396000
1	-2.648642000	-2.999801000	3.341564000
1	-3.046315000	-1.282039000	2.984261000
1	-2.191221000	-1.725722000	4.502301000
35	-7.519109000	-1.613947000	2.702639000
8	-5.108150000	-0.583723000	2.353246000
1	-5.501141000	0.276941000	2.609903000
8	-10.518242000	-0.740477000	-1.651233000
1	-9.957304000	-0.178577000	-1.065632000
1	-10.039758000	-1.606531000	-1.652089000
8	-8.890010000	0.761008000	0.057105000
1	-8.023257000	0.951316000	-0.389541000
1	-8.662803000	0.238912000	0.847772000
8	-12.271272000	-1.848252000	0.070768000
1	-11.744330000	-1.320924000	-0.594637000
1	-12.522690000	-1.212681000	0.780416000
8	-5.275519000	-0.728350000	-0.155365000
1	-5.316551000	-0.700072000	0.907115000
1	-5.880080000	-1.427397000	-0.489862000
8	-8.215916000	-6.832078000	0.252730000
1	-9.162762000	-6.613688000	0.465706000
1	-7.691739000	-6.208396000	0.782612000
8	-9.288642000	-3.178406000	-1.211556000
1	-8.350632000	-3.021298000	-1.485594000
1	-9.317770000	-3.024825000	-0.230322000
8	-9.906352000	-2.524232000	1.315744000
1	-10.808501000	-2.285147000	0.985911000
1	-10.059151000	-3.186982000	2.025371000
8	-11.694151000	-4.450830000	-0.950916000
1	-12.071914000	-3.655033000	-0.516126000
1	-10.825135000	-4.126353000	-1.283513000
8	-10.990932000	-4.397766000	3.075268000
1	-10.790436000	-4.837865000	3.934143000
1	-11.058021000	-5.102161000	2.387641000
8	-10.807151000	-6.125568000	0.916705000

1	-11.386180000	-6.905282000	0.909922000
1	-11.177723000	-5.514876000	0.204007000
8	-6.481532000	1.383153000	-1.083436000
1	-6.472189000	1.377285000	-2.054133000
1	-5.950284000	0.566716000	-0.787282000
8	-3.096106000	-2.313710000	-0.288933000
1	-3.805874000	-1.622006000	-0.312147000
1	-2.230897000	-1.863218000	-0.125392000
8	-5.627396000	-4.396188000	-3.116267000
1	-4.734190000	-4.463138000	-2.691949000
1	-6.236334000	-5.039209000	-2.665457000
8	-6.782186000	-2.352728000	-1.863371000
1	-6.980611000	-1.657053000	-2.510838000
1	-6.279215000	-3.053593000	-2.379586000
8	-7.502667000	-6.227017000	-2.425953000
1	-7.683626000	-6.514188000	-1.504485000
1	-6.906659000	-6.896417000	-2.834069000
1	-11.976214000	4.792580000	1.507840000
1	-13.308853000	4.016259000	1.639481000
1	-13.343059000	0.108952000	2.578464000
1	-11.900957000	0.625836000	2.268198000
1	-10.563177000	4.574981000	-0.151262000
1	-11.255372000	5.968617000	-0.336407000
1	-8.848121000	5.616928000	0.653439000
1	-8.217729000	4.439735000	1.446219000
1	-12.586718000	-1.776987000	5.561967000
1	-13.991620000	-1.635796000	6.243971000
1	-10.127944000	2.326324000	2.404444000
1	-11.613394000	2.878410000	2.429156000
1	-11.844427000	-1.283212000	3.517008000
1	-11.635451000	-2.828844000	3.732188000
1	-9.550003000	2.488744000	-0.308487000
1	-8.787312000	3.817997000	-0.491850000
1	-10.876091000	1.811874000	4.343728000
1	-10.154268000	2.649156000	5.414306000
1	-7.716697000	2.698118000	2.608766000
1	-8.690568000	3.610188000	3.455903000
1	-12.528210000	0.063224000	6.973200000
1	-11.707644000	1.305300000	6.474714000
1	-9.221310000	5.018088000	5.314696000
1	-8.223738000	3.864286000	5.692777000
8	-13.040900000	-1.617776000	6.438475000
8	-12.469877000	-0.195431000	2.282848000
8	-11.614290000	-1.962324000	4.201304000
8	-12.217085000	0.954952000	7.255463000
8	-9.009631000	4.089915000	5.128409000
8	-10.576658000	1.773943000	5.282977000
8	-12.540279000	4.300975000	2.159102000
8	-11.066280000	2.058164000	2.576119000
8	-10.835314000	5.405189000	0.333274000
8	-9.575304000	3.328094000	-0.817488000
8	-8.029701000	5.080774000	0.702772000
8	-8.575863000	3.208630000	2.558259000
1	-5.719886000	2.264776000	1.824825000
1	-5.668002000	2.767450000	3.265252000
1	-4.781113000	4.427742000	4.643889000
1	-3.899997000	4.191340000	3.364859000
1	-5.032509000	3.847810000	0.574813000
1	-5.421550000	2.542735000	-0.279794000
1	-6.851663000	2.282841000	6.272725000
1	-7.457952000	2.968311000	7.529862000
1	-5.389745000	5.163460000	2.233679000
1	-6.356816000	5.549448000	1.047083000
8	-4.845274000	4.263715000	3.672448000
8	-6.978868000	3.162493000	6.670219000
8	-6.131860000	2.088064000	2.710225000
8	-4.834620000	2.885270000	0.437735000
8	-5.401754000	5.436437000	1.282868000
1	-5.307585000	3.797486000	6.639906000
1	-4.339851000	4.996743000	6.882893000
8	-4.429105000	4.172798000	6.377975000
1	-10.058009000	-4.203449000	5.774787000
1	-10.048973000	-5.755819000	6.024620000
1	-9.542036000	-2.400897000	6.690209000
1	-10.034074000	-2.206780000	5.183412000
1	-11.637995000	-4.318202000	7.225126000
1	-12.498263000	-3.046686000	7.423483000
1	-10.989909000	0.689880000	8.272337000
1	-10.561640000	0.543413000	9.778343000
1	-10.614558000	-2.744874000	8.370442000
1	-9.984454000	-1.302277000	8.569876000
1	-9.011935000	1.812161000	8.822329000
1	-7.863291000	2.420308000	9.681327000
8	-12.025722000	-3.750250000	7.927081000
8	-10.631127000	-5.030438000	5.745986000
8	-9.391889000	-2.693868000	5.746755000
8	-9.762098000	-2.238718000	8.362009000
8	-10.198904000	0.491737000	8.878618000
8	-8.383568000	2.579156000	8.878827000
1	-2.166447000	3.682121000	2.058413000
1	-2.381349000	5.223573000	2.343046000
1	-0.113447000	-2.138174000	-0.190203000
1	-0.322482000	-0.558981000	-0.216611000
1	1.252653000	-3.795278000	-1.263929000
1	-0.039871000	-3.411357000	-2.065346000
1	-0.711222000	1.726665000	-0.323648000
1	0.272996000	1.167496000	0.656201000
1	-3.866177000	6.367164000	1.250696000
1	-2.508610000	6.534003000	0.533212000
1	-3.120454000	2.473699000	0.590738000
1	-1.964706000	1.683989000	1.369562000
1	-0.166431000	0.781079000	5.383215000
1	-1.323084000	1.784723000	5.086479000
1	-0.786660000	-0.355856000	1.904518000
1	-0.851887000	0.576504000	3.209799000
1	-1.863957000	3.820689000	4.477585000
1	-2.673956000	3.501177000	5.764673000
8	-0.593156000	-1.391907000	0.240645000
8	0.132387000	1.225658000	-0.310303000
8	0.297137000	-3.660993000	-1.155478000
8	-0.851905000	0.567360000	2.218364000
8	-2.154774000	2.474478000	0.806742000
8	-2.926116000	6.628809000	1.404066000
8	-2.287659000	4.343485000	2.793079000
8	-1.784673000	3.405163000	5.367273000
8	-1.017420000	0.846585000	4.922325000
8	-4.528200000	-9.524661000	-0.170566000
1	-3.908510000	-9.536626000	0.599036000
1	-5.427506000	-9.424733000	0.224655000
8	-0.911479000	-3.139014000	-3.497355000
1	-1.780452000	-3.490832000	-3.160999000
1	-0.615270000	-3.803207000	-4.140255000
8	-5.395270000	-7.285741000	-3.883463000
1	-5.345704000	-6.332631000	-4.085239000
1	-4.760666000	-7.373329000	-3.125210000
8	-3.898290000	-7.321279000	-1.618942000

1	-4.070407000	-8.150673000	-1.095199000
1	-2.938790000	-7.158559000	-1.596104000
8	-1.339744000	-5.967296000	-1.025956000
1	-0.639428000	-5.273354000	-1.047523000
1	-1.459198000	-6.178851000	-0.074044000
8	-7.032294000	-9.225595000	1.047793000
1	-7.528632000	-8.429750000	0.739428000
1	-7.592472000	-9.977366000	0.799396000
8	-2.657656000	-9.443717000	1.884625000
1	-2.312260000	-8.524561000	1.822628000
1	-1.930719000	-9.992715000	1.550447000
8	-1.804054000	-6.761698000	1.644690000
1	-1.108145000	-6.509158000	2.274789000
1	-2.585284000	-6.240008000	1.906390000
8	-3.069528000	-4.144714000	-2.185710000
1	-3.109965000	-3.419223000	-1.488603000
1	-2.584482000	-4.883047000	-1.738546000

$Int_{MPI,Br}$:

6	-5.290150000	-0.137411000	8.336018000
6	-4.648486000	-0.924626000	7.388242000
6	-3.274080000	-1.151077000	7.488563000
6	-2.483923000	-0.613018000	8.494051000
6	-3.138539000	0.181468000	9.441677000
6	-4.516994000	0.411659000	9.366842000
6	-5.143594000	-1.672804000	6.196299000
6	-3.899186000	-2.335023000	5.655171000
7	-2.876525000	-2.022889000	6.425909000
1	-6.353207000	0.068980000	8.271018000
1	-1.414575000	-0.778146000	8.540353000
1	-2.562426000	0.627152000	10.246219000
1	-4.993185000	1.032968000	10.118854000
1	-5.986835000	-2.362911000	6.358391000
6	-3.851745000	-3.184618000	4.472996000
6	-4.863263000	-4.149542000	4.282270000
6	-2.844344000	-3.024342000	3.500913000
6	-4.824638000	-4.982159000	3.168463000
6	-2.850610000	-3.825820000	2.359787000
6	-3.824501000	-4.818610000	2.202716000
1	-5.656752000	-4.261537000	5.014580000
1	-2.097305000	-2.240244000	3.599634000
1	-5.574087000	-5.755955000	3.040068000
1	-2.113628000	-3.643025000	1.582796000
1	-3.815848000	-5.453852000	1.320086000
6	-1.501296000	-2.526912000	6.336638000
1	-1.470168000	-3.372277000	5.653694000
1	-0.856829000	-1.718467000	5.988914000
1	-1.197259000	-2.845284000	7.335419000
35	-5.827233000	-0.441370000	4.777473000
8	-4.406608000	-0.545901000	1.553987000
1	-5.047646000	0.031216000	2.021909000
8	-8.392768000	-1.118257000	-1.343409000
1	-8.701116000	-0.200579000	-1.570986000
1	-7.502761000	-0.960368000	-0.960573000
8	-8.896576000	1.554018000	-1.526366000
1	-7.869023000	1.650721000	-1.484840000
1	-9.225529000	1.551672000	-0.595013000
8	-9.628909000	-2.277061000	0.746090000
1	-9.271251000	-1.743192000	-0.016356000
1	-10.104753000	-1.658511000	1.340216000
8	-5.525644000	-0.649464000	-0.978445000
1	-4.827988000	-0.698769000	0.675596000
1	-5.690144000	-1.316838000	-1.681473000
8	-6.875624000	-7.114995000	0.025085000
1	-7.789370000	-7.128723000	0.401701000
1	-6.697026000	-6.144094000	-0.077292000
8	-6.901783000	-4.440378000	-0.346551000
1	-6.602324000	-3.843678000	-1.077252000
1	-6.902187000	-3.894858000	0.490927000
8	-7.253277000	-3.033054000	1.910597000
1	-8.106634000	-2.673373000	1.560933000
1	-7.545633000	-3.770030000	2.491999000
8	-9.641062000	-4.695724000	-0.561223000
1	-9.893741000	-3.884157000	-0.064214000
1	-8.656663000	-4.595578000	-0.633000000
8	-8.665608000	-4.997622000	3.291325000
1	-8.592436000	-5.477781000	4.151314000
1	-9.014341000	-5.634154000	2.617867000
8	-9.351384000	-6.628015000	1.197028000
1	-10.092375000	-7.253651000	1.229830000
1	-9.605870000	-5.920244000	0.521678000
8	-6.318793000	1.700008000	-1.559315000
1	-6.085018000	1.983851000	-2.456616000
1	-5.792983000	0.295814000	-1.325215000
8	-2.898039000	-1.426651000	-1.744241000
1	-3.709020000	-1.033793000	-1.354522000
1	-2.236944000	-1.511418000	-1.003749000
8	-5.327367000	-4.934115000	-3.645800000
1	-4.572410000	-4.741560000	-3.035881000
1	-5.887223000	-5.625104000	-3.202877000
8	-6.407448000	-2.776248000	-2.469392000
1	-7.325441000	-2.486347000	-2.623404000
1	-6.148271000	-3.470933000	-3.132832000
8	-6.723175000	-7.158133000	-2.797135000
1	-6.790524000	-7.270592000	-1.822109000
1	-5.886620000	-7.614254000	-3.055529000
1	-11.625430000	4.086366000	0.394268000
1	-12.550756000	3.009534000	-0.222285000
1	-11.432592000	-0.490302000	2.899178000
1	-10.347774000	0.319484000	2.084542000
1	-10.103366000	4.997399000	-0.871728000
1	-11.065278000	6.163041000	-0.440837000
1	-8.648869000	5.624096000	0.584734000
1	-7.992653000	4.369603000	1.209183000
1	-11.005324000	-2.856407000	5.269031000
1	-12.544129000	-3.120968000	5.407975000
1	-9.444143000	2.123395000	1.546074000
1	-10.876196000	2.100069000	0.887884000
1	-9.691062000	-1.966123000	3.695862000
1	-9.413913000	-3.527277000	3.820438000
1	-9.034692000	3.310810000	-1.843129000
1	-8.257970000	4.469535000	-1.166995000
1	-9.843750000	0.332977000	4.290969000
1	-9.823109000	1.718381000	5.006530000
1	-7.507281000	2.486098000	2.367627000
1	-8.568434000	3.317971000	3.225309000
1	-11.760842000	-1.192896000	6.518823000
1	-10.975970000	0.126235000	6.219551000
1	-9.620502000	4.255507000	5.204010000
1	-8.509505000	3.200191000	5.572563000
8	-11.754019000	-2.902522000	5.927843000
8	-10.476604000	-0.457491000	2.725387000
8	-9.603991000	-2.698606000	4.338195000
8	-11.625205000	-0.273872000	6.851196000
8	-9.174328000	3.456979000	4.881179000
8	-9.688423000	0.757247000	5.164831000

8	-12.126162000	3.257960000	0.614143000
8	-10.070605000	1.530661000	1.040980000
8	-10.568737000	5.409109000	-0.084118000
8	-8.928624000	4.296079000	-1.864663000
8	-7.807168000	5.124116000	0.587319000
8	-8.307814000	3.082331000	2.300905000
1	-5.668622000	1.954790000	1.513637000
1	-5.638327000	2.209818000	3.031892000
1	-5.084921000	3.573420000	4.730264000
1	-4.035188000	3.500104000	3.559775000
1	-5.024362000	3.659916000	0.484227000
1	-5.463014000	2.427697000	-0.556290000
1	-7.914110000	1.292664000	6.042238000
1	-7.943485000	1.956835000	7.462306000
1	-5.348659000	4.781398000	2.382204000
1	-6.143908000	5.453587000	1.193857000
8	-5.004291000	3.642258000	3.747220000
8	-7.566396000	2.061882000	6.541318000
8	-6.067466000	1.604087000	2.367956000
8	-4.866054000	2.718526000	0.243203000
8	-5.239005000	5.199917000	1.495296000
1	-5.859265000	2.470618000	6.465233000
1	-5.019612000	3.646995000	7.044746000
8	-4.988806000	2.940866000	6.379681000
1	-8.167409000	-4.882668000	6.132749000
1	-8.025518000	-6.443962000	6.266371000
1	-8.166194000	-3.094202000	7.227045000
1	-8.297515000	-2.846182000	5.657283000
1	-10.097078000	-5.346634000	7.037394000
1	-11.204232000	-4.263113000	7.020157000
1	-10.727997000	-0.464179000	8.179694000
1	-10.700202000	-0.572003000	9.752962000
1	-9.673400000	-3.742994000	8.430152000
1	-9.400804000	-2.234764000	8.805879000
1	-9.152278000	0.868619000	9.014350000
1	-7.991818000	1.649936000	9.703981000
8	-10.749909000	-4.898988000	7.619976000
8	-8.615272000	-5.754374000	5.921416000
8	-7.726317000	-3.271332000	6.338525000
8	-8.956345000	-3.106367000	8.686365000
8	-10.113354000	-0.576611000	8.979247000
8	-8.632596000	1.715124000	8.979664000
1	-2.451527000	2.548828000	2.449888000
1	-2.301714000	4.118463000	2.609330000
1	-0.343644000	-2.386304000	-0.451038000
1	-0.566676000	-1.055994000	0.384677000
1	1.410991000	-3.774311000	-1.704654000
1	0.070744000	-3.600385000	-2.520907000
1	-0.945057000	0.951554000	0.906206000
1	-0.092098000	0.206279000	1.954875000
1	-3.487283000	5.600337000	1.482236000
1	-2.177696000	5.314233000	0.720029000
1	-3.324890000	2.024059000	0.608515000
1	-3.187372000	0.662073000	1.322097000
1	0.305217000	1.010707000	6.227985000
1	-1.191657000	1.338597000	5.936521000
1	0.535917000	-1.152077000	3.656991000
1	-0.334940000	-0.089593000	4.383328000
1	-2.179556000	2.936518000	4.880123000
1	-3.125298000	2.550546000	6.053729000
8	-0.992066000	-1.912713000	0.112864000
8	-0.078981000	0.492741000	1.010510000
8	0.461522000	-3.607287000	-1.599566000
8	-0.274028000	-0.629146000	3.548974000
8	-2.658714000	1.481005000	1.108693000
8	-2.508043000	5.562129000	1.598126000
8	-2.406706000	3.278489000	3.128689000
8	-2.181386000	2.693705000	5.835034000
8	-0.524830000	0.591832000	5.951529000
8	-3.294758000	-9.332462000	0.536303000
1	-2.477795000	-9.249358000	1.083998000
1	-4.016447000	-8.990549000	1.118684000
8	-0.918904000	-3.770265000	-3.888887000
1	-1.777977000	-3.908906000	-3.403200000
1	-0.713635000	-4.639813000	-4.268107000
8	-4.153575000	-7.706792000	-3.664887000
1	-4.270243000	-6.801326000	-4.008983000
1	-3.770326000	-7.554491000	-2.757604000
8	-3.368202000	-7.181670000	-1.125063000
1	-3.278309000	-7.991787000	-0.552981000
1	-2.521396000	-6.695228000	-1.041432000
8	-1.336969000	-5.385259000	-0.556237000
1	-0.564786000	-4.880116000	-0.910667000
1	-1.109457000	-5.612225000	0.370818000
8	-5.325087000	-8.159437000	2.010627000
1	-5.878920000	-7.780695000	1.276906000
1	-5.897186000	-8.825029000	2.424083000
8	-0.967351000	-9.048947000	2.057541000
1	-0.841069000	-8.074354000	2.091629000
1	-0.262301000	-9.356008000	1.465862000
8	-0.676357000	-6.245101000	2.057402000
1	0.212842000	-5.960412000	2.328223000
1	-1.278395000	-5.824424000	2.696125000
8	-3.099231000	-4.034386000	-2.278378000
1	-3.140214000	-3.057508000	-2.042690000
1	-2.677584000	-4.479599000	-1.509476000
